# Structural Analysis of Molecular Materials Using the Pair Distribution Function

**DOI:** 10.1021/acs.chemrev.1c00237

**Published:** 2021-11-17

**Authors:** Maxwell W. Terban, Simon J. L. Billinge

**Affiliations:** † Max Planck Institute for Solid State Research, Heisenbergstraße 1, 70569 Stuttgart, Germany; ‡ Department of Applied Physics and Applied Mathematics, Columbia University, New York, New York 10027, United States; ¶ Condensed Matter Physics and Materials Science Department, Brookhaven National Laboratory, Upton, New York 11973, United States

## Abstract

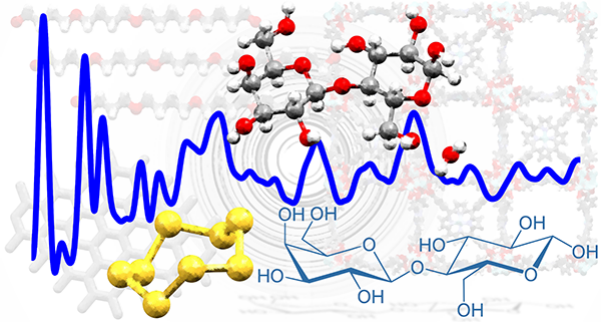

This is a review of atomic pair distribution function (PDF) analysis as applied to the study of molecular materials. The PDF method is a powerful approach to study short- and intermediate-range order in materials on the nanoscale. It may be obtained from total scattering measurements using X-rays, neutrons, or electrons, and it provides structural details when defects, disorder, or structural ambiguities obscure their elucidation directly in reciprocal space. While its uses in the study of inorganic crystals, glasses, and nanomaterials have been recently highlighted, significant progress has also been made in its application to molecular materials such as carbons, pharmaceuticals, polymers, liquids, coordination compounds, composites, and more. Here, an overview of applications toward a wide variety of molecular compounds (organic and inorganic) and systems with molecular components is presented. We then present pedagogical descriptions and tips for further implementation. Successful utilization of the method requires an interdisciplinary consolidation of material preparation, high quality scattering experimentation, data processing, model formulation, and attentive scrutiny of the results. It is hoped that this article will provide a useful reference to practitioners for PDF applications in a wide realm of molecular sciences, and help new practitioners to get started with this technique.

## 1 Introduction

Molecular materials are profoundly useful, with applications from packaging food, to curing disease, to producing and storing energy. Indeed, as living organisms, we are ourselves largely made up of them. Many exciting developments over the past few decades have centered on engineering the molecular entities themselves (e.g., from fullerenes to proteins and catalytic complexes), their arrangements (e.g., amorphous polymer-pharmaceutical blends and cocrystals), and even the spaces between them (e.g., metal– and covalent–organic frameworks). The possibilities increase when considering composite systems where molecular entities can interact with a host or with each other within finite dimensions. In addition to being useful, molecular materials are also structurally complex: a boon for their interesting properties and tunability, but often a challenge to detailed and conclusive atomic-scale structural understanding of their behavior.

Scattering and diffraction of photons, neutrons, and electrons are used for understanding the effects of atomic structuring on material function. When molecular entities can be made to arrange in a periodic fashion, the bonding, conformations, and intermolecular packing or network connectivity can be determined using sophisticated, accessible, and continually developed crystallographic tools for single crystals and polycrystalline materials,^1−3^ and new tools and approaches on the horizon are advancing rapidly.^4−6^ However, structural imperfections and disordered states are ubiquitous and often underlie useful properties. The atomic pair distribution function (PDF) analysis of diffraction data can fill the gap when the samples contain defects, disorder, and/or discrete material structures. In recent years, PDF analysis has been under intense development in this regard and is now also becoming a powerful method to tackle such problems.^7−10^

The PDF of a material may be obtained experimentally by Fourier transformation of a scattering pattern,^11^ revealing direct-space insights into any long-range ordered structure from the Bragg scattering (i.e., diffraction) and short-range structural correlations from the diffuse scattering intensity present. PDF analysis is not a new technique; its origins lie alongside early developments in the field of X-ray crystallography,^12−14^ and it has been an important technique used extensively in the fields of inorganic amorphous materials, including liquids and glasses, for many decades.^15−20^ However, there is currently a revolutionary acceleration in accessibility of the method to general users in materials chemistry communities due to substantial development of dedicated instrumentation and data analysis packages. It is an increasingly important tool within contemporary inorganic materials communities, for instance in studying nanoparticles,^21−23^ magnetic structures,^24^ strongly correlated electron systems,^25^ cultural heritage objects,^26^ and other functional materials.^27−29^

These methods of local structure characterization have also been extended to the characterization of molecular materials, though along different trajectories within various communities of researchers. In this review, we seek to consolidate the advances and methodologies in these areas by covering a broad selection of molecular material classifications benefiting from PDF analysis. Some discussion of historical development is given for relevant material classes, but applications within the past few decades are the main focus. Prominent areas of research highlighted will include nongraphitic carbons, synthetic and natural polymers, small molecules such as pharmaceuticals and industrial liquids, and microporous materials including but not limited to metal–organic frameworks (MOFs) and covalent–organic frameworks (COFs). However, the science of these materials in general will not be comprehensively reviewed. This review is meant as a comprehensive roadmap for the application of PDF analysis to molecular materials and, additionally we hope, will help provide useful insights for people new to the approach to optimize experiments and intuition specifically for molecular materials. We also attempt to provide descriptions that will help users to connect attributes of the measured signal to the structural properties that they encode.

## 2 Distribution Functions

The atomic pair distribution function [PDF, *G*(*r*)] is a simple 1D function that contains information about structural correlations in a material. Why is this function useful? First, it is easy to measure experimentally. Second, it is straightforward to calculate from a known structure. And third, it gives a kind of intuitive local-view of the structure, as if you are sitting on an atom and looking out at your neighborhood. Strictly, it is directly derived from the autocorrelation function of the atomic density,^18^ but intuitively, it yields the probability of finding pairs of atoms separated by some distance, *r*. The structural signal measured corresponds to an average over all local states within the measurement time and sample volume. There are a variety of different forms and normalizations of the PDF, used by various scientific communities, which are discussed in detail elsewhere.^30,31^

### 2.1 The Radial Distribution Function

**Figure 1:**
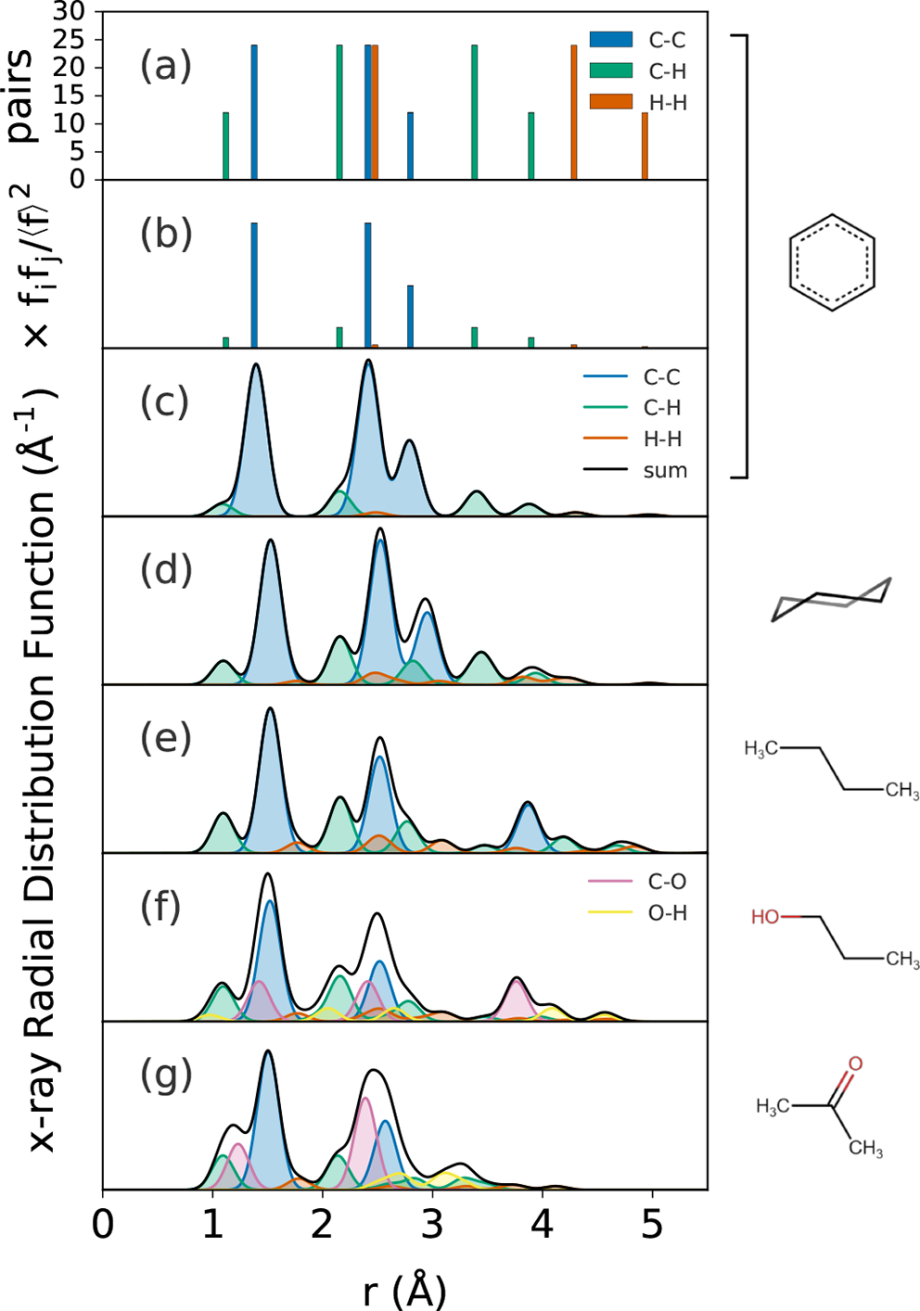
Pair distributions for simple molecules: (a) Histogram of interatomic pair distances for a single molecule of benzene, (b) weighted by the relative X-ray scattering powers of the atom pairs, and (c–g) the RDFs simulated from individual molecules of benzene, cyclohexane, butane, propanol, and acetone, allowing for some thermal motion of the atoms that broadens the histogram into a continuous function. Only the single conformations shown were considered.

Starting in real space (also known as physical space or direct space), we introduce the radial distribution function [RDF, *R*(*r*)]. This function is a scaled histogram of every interatomic distance in the material. It is made by sitting on each atom *i* in turn, measuring the distance from this atom to every other atom *j* in the material, and adding a “count” to the bin at each distance *r*_*ij*_ on a histogram plot, as shown for a molecule of benzene in Figure 1a.

For multielement materials, every “count” has a weight that is determined by the relative scattering power of each pair of atoms for a given scattering probe (i.e., X-rays, neutrons, or electrons) (see section 7.1). Representing this weight as *b*, we get
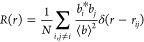
1where ⟨*b*⟩ is the stoichiometric average of the weights, and the asterisk denotes the complex conjugate of the weight. The properties of a delta function are that ∫_below_^above^δ(*r*)d*r* = 1. Thus, for a particular coordination shell, the (scattering power weighted) average number of neighboring atoms can be obtained by integrating *R*(*r*) from below to above the coordination shell according to
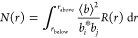
2

By way of example, in Figure 1b we plot this histogram using the weights appropriate for an X-ray experiment. The so-called form factors for X-rays are generally written as *f* (explicitly *f*(*Q*)), and are heavily damped at increasing *Q* values. Assuming that this *Q*-dependence is perfectly removed, the weights in real space for X-rays are then the number of electrons on each atom in the pair. In practice, removing the dependence is often not perfect, and involves the use of varying assumptions that can have an effect on the final data—see Secion 7.1.1 for further discussion. We see that it is relatively easy to see C–C pairs, difficult to see C–H pairs, and nearly impossible to see H–H pairs in this case. Hydrogen scatters neutrons relatively more strongly, making them the probe of choice for its investigation. Finally, in a real system, the “counts” in each bin are replaced by Gaussian functions due to the fluctuations of atom-pair distances by thermal motion, to approximate the real RDF, Figure 1c.

Benzene is an aromatic hexagon with just three unique interatomic (non-hydrogen) distances: the C–C nearest neighbor (NN) distance at *r* = 1.395 Å, the second neighbor at *r* = 2.416 Å, and the longest C–C distance across the diameter of the ring at *r* = 2.790 Å, just where the peaks appear in the function *R*(*r*). In addition to the weighting of the scattering power, the size of the peaks (the integrated area) is determined by the number of neighbors, or multiplicity at that length. In benzene, every C atom has two nearest neighbors (NN) and two second NN carbon atoms, so these distances show peaks with the same size. There is only one-third NN giving a peak that is half as big. For cyclohexane, Figure 1d, the number of C–C neighbors in each coordination shell is the same, but the longer, single C–C bonds in the chair conformation are extended to *r* = 1.526 Å, so shifts of all the peaks to slightly higher distances are observed.

In butane, Figure 1e, the diametric C–C distance of the cyclic molecules is replaced by a longer distance of *r* = 3.871 Å between the C atoms on either end of the antiperiplanar conformation. For the acyclic molecule, the average NN coordination is now 1.5 instead of 2, leading to a smaller first peak than in the previous two cases. To compute the average number of neighbors, count the total number of neighbors at a particular distance in the entire molecule, and divide by the number of atoms in the molecule. There are 6 C–C NN pairs (the first atom has one, the second has two, the third has two, and the fourth has one, totalling six) and there are four C atoms, so the average C–C coordination is 6/4 = 1.5. Applying the same approach to the second neighbor distance gives 4/4 = 1.0, so the average number of second neighbor correlations in butane is 1.0, and for the third neighbor it is 2/4 = 0.5, leading to relatively lower intensity in the second and third peaks compared to the cyclic molecules.

An oxygen atom is introduced in the propanol molecule, Figure 1f. The RDF becomes more complicated, because the interatomic distance and weighting of the C–O bond is similar to, but slightly different from, the C–C bond in butane. The C–O bond in propanol at *r* = 1.421 Å  instead of ⟨*r*⟩ = 1.520 Å for C–C, resulting in a shoulder on the short distance side of the first PDF peak. Oxygen is a slightly stronger X-ray scatterer than C (*Z* = 8 electrons instead of 6) but there are twice as many C–C bonds, and so the C–C peak still appears larger. The third peak in propanol comes only from C–O, rather than its C–C counterpart in butane, and is thus slightly shorter in distance and relatively larger in intensity. In practice, it is difficult to resolve distinct C–O and C–C peaks, however C=O as in acetone, Figure 1g, or, for example, in amides, can be resolved with high real-space resolution PDF measurements.^32^

**Figure 2:**
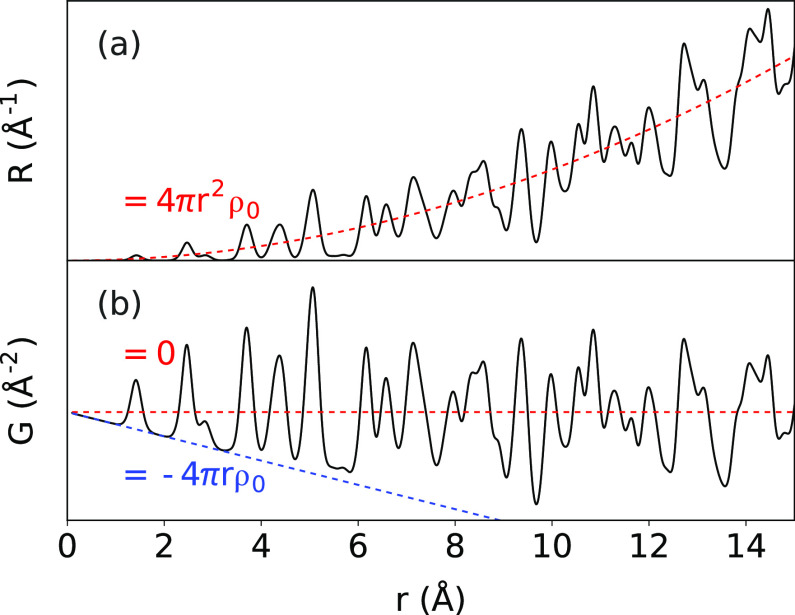
Asymptotic behavior of the RDF and reduced PDF: (a) The RDF (black) is shown with the asymptotic behavior shown in red, and (b) the associated PDF (black), with asymptotic behavior in red and negative sloping baseline in blue.

The RDF can become inconvenient when it is used to study bulk systems of continuous matter. As *r* increases, the number of interatomic distances that are found in the associated “bin” scales with the differential increase in the number of atoms associated with the spherical volume, ∝ 4*πr*^2^ρ_0_, as shown in Figure 2a, where ρ_0_ is average atom number density. As it turns out, this scaling is not so convenient for viewing structural features beyond the first few coordination shells. A more tenable function to deal with can be obtained by normalizing the RDF by the spherical area and ρ_0_ to get a *reduced* PDF by
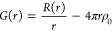
3

This happens to define the eponymous PDF. Other normalizations to deal with the divergence of *R*(*r*) are thoroughly discussed^30^ and clearly illustrated with various advantages,^31^ but this choice has two significant advantages. First, it also happens to be the function obtained from the direct Fourier transformation of the properly normalized powder diffraction pattern and, second, errors in this function propagated from the measurement uncertainties are roughly constant in *r*, so features in the *G*(*r*) function at different *r*-values carry equal weight and can be visually compared in a meaningful way. As will be discussed below, the more general form is given as
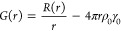
4where γ_0_ is the characteristic function describing the autocorrelation of the shape of the scattering domains or crystallites in the material.^33^ This form is important for nanoparticulate or nanostructured materials, but reduces to eq 3, γ = 1, for very large crystals.

### 2.2 Distribution Functions from Scattering

We confine our discussion to elastic scattering processes, where no energy is exchanged between the probe and the material. Then, the magnitude of the scattering vector can be defined as
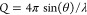
5where λ is the wavelength of the probe, and the angle formed between the incident and scattered directions is by convention 2θ (Note: the energy dependent 2θ is typically used as the independent variable for Rietveld refinements, in part because many abberations to the instrumental profile are specifically angle dependent, and in part out of habit). Physically, the magnitude of *Q* = 2π/*r* is a sinusoidally varying signal in the diffraction pattern corresponding to scatterers separated by a distance *r*. If scatterers are spaced periodically, extending over long distances, the scattering intensity in *Q* is in the form of sharp Bragg peaks. If the spatial relationship only exists over short distances, then the scattering intensity in *Q* is broad (diffuse scattering).

Without making any assumptions about the arrangement of atoms in a material, the isotropic, coherent scattering intensity for the collection of atoms is given by
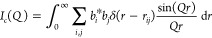
6which is a form of the Debye scattering equation.^34^ This is the objective function of any powder diffraction measurement (additional effects due to the geometry of the measurement, the shape and size of the sample, characteristics of the irradiating source, and removal of inelastic scattering, are all well documented).^2,35^ The total scattering structure function *S*(*Q*) is the interference function obtained after normalizing by the scattering factors and number of scatterers, given by the commonly used Faber-Ziman formalism^36^ as
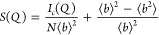
7Equation 6 shows the observed scattering results directly from the RDF of the material (and vice versa). The self-scattering part, when *i* = *j* or ⟨*b*^2^⟩/⟨*b*⟩^2^, is separated. This occurs when *r*_*ij*_ = 0, where sin(*Qr*)/(*Qr*) goes to 1, allowing it to be taken out of the integral. A combination of these definitions and some manipulation then gives
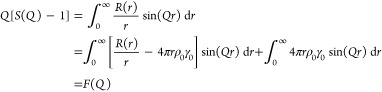
8

**Figure 3:**
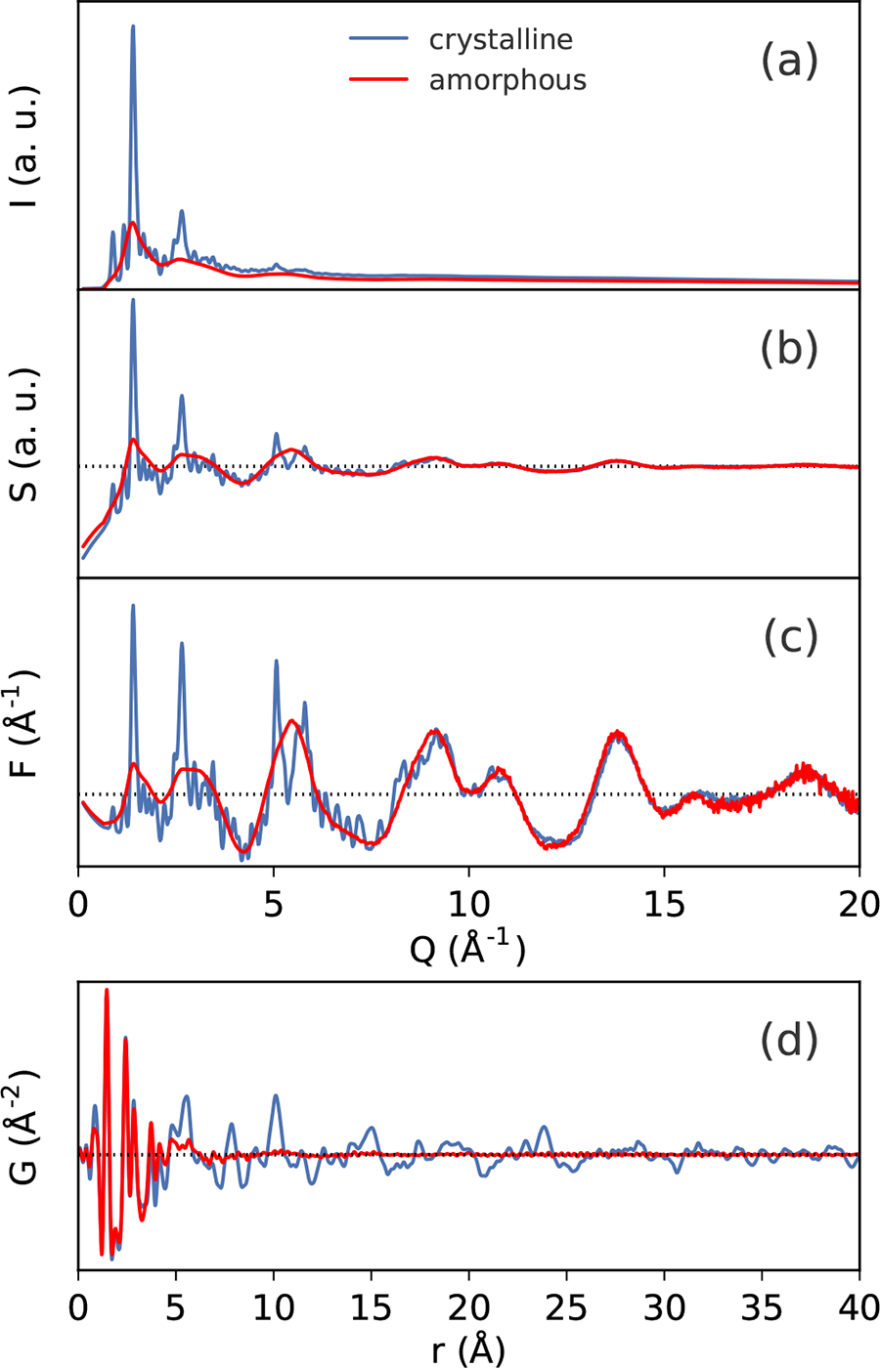
Examples of the various scattering functions and the PDFs of crystalline (blue) and amorphous (red) lactose: (a) background subtracted scattering intensities *I*(*Q*), (b) the total scattering structure function *S*(*Q*) (dashed line = 1), (c) the reduced structure function *F*(*Q*) (dashed line = 0), and (d) the PDF, *G*(*r*) (dashed line = 0). The data are from Terban et al.^38^

The integral in line two of eq 8 now contains the definition of *G*(*r*) given above. The integral in line three is generally neglected since its contribution is primarily in the small angle region, which is not measured in a typical experiment. The absence of this term in the experimental and simulated data does however have an effect on the shape of the baseline in *G*(*r*).^33,37^*F*(*Q*) is the reduced total scattering structure function. It is important to consider that these *structure functions* are different from the crystallographic *structure factor F*_*hkl*_ encountered in Bragg peak analyses, which is only summed over atoms in a unit cell. Finally, the functions *F*(*Q*) and *G*(*r*) are related by a sine Fourier transform. Thus, by inverting, we arrive at a functional relationship for obtaining a PDF from an experimental scattering pattern:
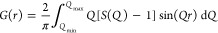
9where in a real experiment, *Q* can only be measured over a discrete range. For comparison, examples of the experimental X-ray scattering patterns *I*(*Q*), normalized structure functions *S*(*Q*) and *F*(*Q*), and PDFs *G*(*r*) are shown for crystalline and amorphous states of the same molecule, respectively, in Figure 3.

In the following sections, major areas dealing with molecular materials and components where PDF analysis has been playing an important role are highlighted. We will then continue in more detail with the methodological developments and tools available for chemical structure analysis.

## 3 Applications

### 3.1 Nongraphitic Carbons

Nongraphitic carbons are a large class of materials, often products of combustion or pyrolysis, including chars, blacks, cokes, soot, hard carbon, glassy carbon, and so on. These materials play large-scale industrial roles (e.g., in energy generation and storage, purification, agriculture, pigments, and automobile tires). This has motivated a long history of detailed structural characterization to help enhance strategies for improving property-bearing characteristics such as surface area, porosity, and crystallite size.^39,40^

Scattering methods have played a critical role in understanding disordered carbon structures, as reviewed by Burian et al.,^41^ often employing PDF analysis for making physical sense of complicated diffraction patterns. This goes all the way back to 1934: Bertram Warren’s use of “Fourier integral analysis” to identify that carbon black contains graphite-like layers.^42^ This sparked developments in the theory of 2D disorder in layered materials and the coining of turbostratic disorder, “unordered layers”, to distinguish the structure of carbon black from graphite crystallites.^43,44^

**Figure 4:**
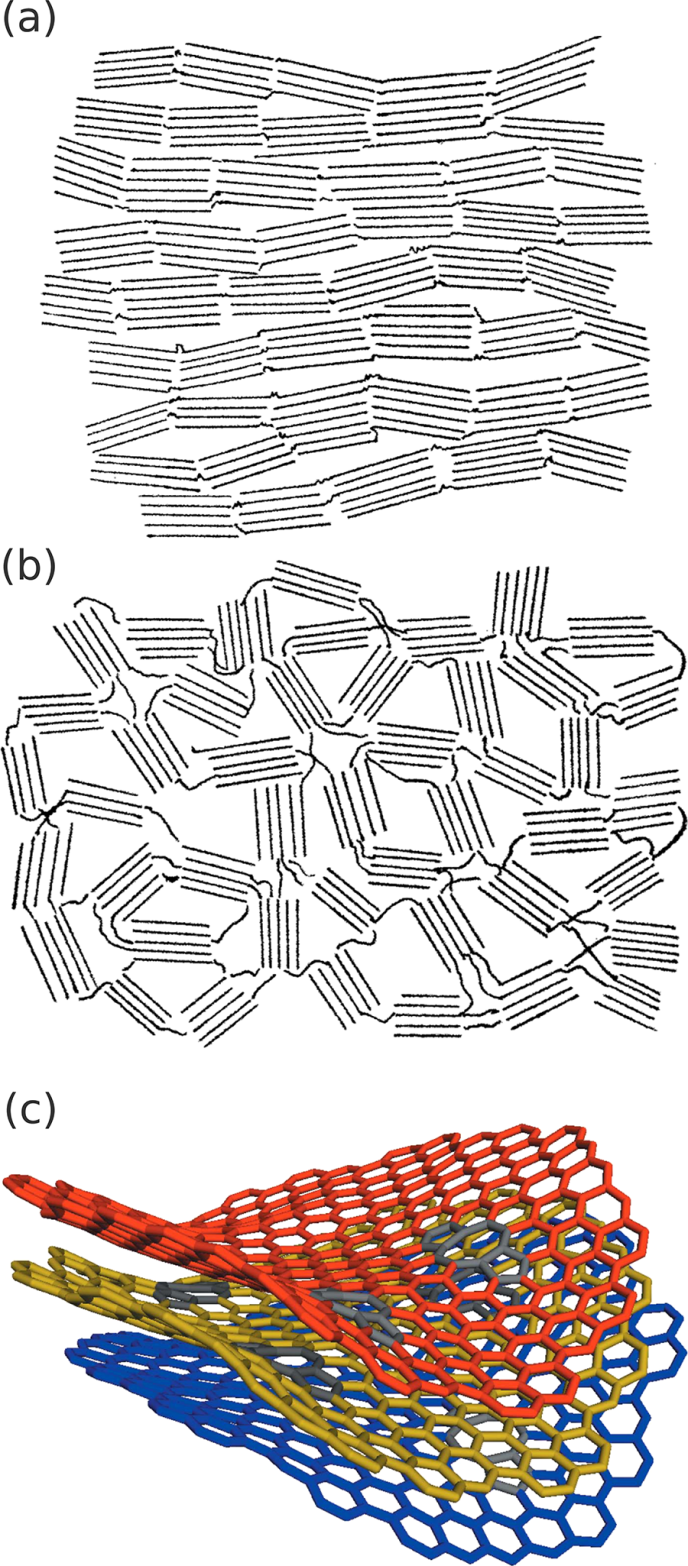
Carbon models, old and new: (a) graphitizing carbon and (b) nongraphitizing carbon, reproduced with permission from ref (47). Copyright 1934 Royal Society. (c) Nongraphitic carbons (with ring defects highlighted in gray) reproduced with permission from ref (49). Copyright 2015 International Union of Crystallography.

Prior to her work on DNA, Rosalind Franklin also made longstanding contributions to the field of carbons.^45^ In 1950, she published, up to that time, the most detailed, quantitative analysis of nongraphitic carbon using and advancing the Fourier method.^46^ She determined that pyrolyzed polyvinylidene chloride was primarily arranged in bi- or few-layer stacks of nonoriented, graphite-like layers, approximately 15 Å in diameter, with a mean separation of about 26 Å. She ascribed a minority fraction in more highly disordered states to the layer edges and noted, “the nature and extent of the non-organized part and the mutual disposition of the crystallites must be of supreme importance in determining the course of any subsequent structural changes induced by thermal or other treatment.”^46^ This led to the classifications of graphitizing and nongraphitizing carbons, depending on whether they can reorient and crystallize on heating.^47,48^ She proposed models in which nongraphitizing carbons consist of a system of strong cross-linking that unites the crystallites into a rigid, finely porous mass, while graphitizing carbons show lesser cross-linkage leading to a more compact structures with nearly parallel orientation, Figure 4a,b

Over the ensuing decades, with improvements in X-ray technologies and advances in neutron sources, the desire to more precisely elucidate the nature of the bonding in these noncrystalline carbons was coupled with a race to achieve higher real-space resolution and better quality data.^50−55^ Structures were found to be significantly affected by the presence of micropores and cavities and likely to contain distortions away from perfectly planar hexagonal layers. The similarity of real-space data to energetically favorable models of Schwarzite-type carbon structures^56^ and observations of nonplanar structures by high-resolution transmission electron microscopy,^57,58^ further indicated the likelihood for non-six-membered rings that could facilitate the curvature in the small graphite-like layers. Effects of both pentagonal and heptagonal defects and curvature were incorporated into modeling schemes to study the effect of nonplanarity on the PDFs, which led to fullerene-like fragments with improved agreement found for nanoporous carbons.^59^ Simple atomic models constructed for nanoporous carbons processed at different temperatures further demonstrated the turbostratic layer relationship. Lower temperatures result in structures with more defective layers due to distributions of higher-number rings and increased curvature that reduces atomic coherence, in the limit of more open structures that no longer resemble graphite-like moieties.^60^

Advanced simulation and modeling methods have helped to develop increasingly detailed models to describe the experimental data and capture particular physical insights into a variety of carbon nanostructures.^61−68^ For instance, microcrystalline, turbostratically disordered, and paracrystalline carbon models have been tested against activated carbons from saccharose with paracrystalline models of small coherently scattering domains, approximately 24 Å in diameter and only several layers, giving the best agreement. The results are still in broad agreement with Franklin’s models nearly 70 years earlier.^68^ The paracrystal model is visualized in Figure 4c.^49^ The addition of molecular dynamics has allowed for a more realistic incorporation of various defects including monovacancies (one atom missing, forming one nonagon and one pentagon), divacancies (two atoms missing, forming two pentagons and one octagon), and Stone-Thrower-Wales defects where 90° rotations of some C–C bonds form defects with two pentagons and two heptagons.^68^ Systematic measurements have allowed for more distinctly resolving pair distances corresponding to longer third NN distances in higher-order rings.^69^ Another model suggests the presence of oligomeric-type carbon matrices surrounding small islands of more perfect graphite-like flakes.^70^ Similar analyses continue to be of importance in characterizing the effect of different feedstock materials and processing conditions on carbon composite materials.^71^ More exotic molecular carbon structures have been investigated, including Buckminsterfullerene (C_60_),^72−74^ C_70_ fullerene,^75^ single- and multiwalled carbon nanotubes,^76−79^ nano-onions,^80^ nanohorns,^81,82^ and sp^3^ bonded, benzene-derived carbon nanothreads,^83^ as well as other nongraphitic, carbon-based, and related materials.^84−91^

### 3.2 Organic Macromolecular Polymers

Natural polymeric materials such as leathers and natural rubber have long been used, as early as the Neolithic period, while the development of modified natural and synthetic polymers (e.g., vulcanized rubber, polystyrene, and polyaniline) began around the mid-19th century.^92^ However, it was the development of thermosetting and thermoplastic polymers in the early 20th century that led to a boom in widespread commercial use, for example, Bakelite by Leo Baekeland in 1909, polyamide (Nylon) 6,6 in 1935 (Wallace Carothers at DuPont), and polyamide 6 in 1938 (Paul Schlack at IG Farben). Coincidence with the advancing techniques of X-ray crystallography meant that investigations into the structures of cellulose derivatives^93^ and synthetic linear polymers^94^ were already extensive by the late 1930s, with a strong caveat important in this endeavor—a lack of single crystals for characterizing high molecular weight polymers. This led also to an early adoption of real-space characterization for disordered structures in the polymer community.

#### 3.2.1 Synthetic Polymers

In 1936, Simard and Warren applied the Fourier integral technique to amorphous rubber.^95^ They demonstrated that the positions and numbers of the first few interatomic coordination shells were in good agreement with the intramolecular structure associated with the chain molecule picture of polyisoprene. They suggested that the first sharp diffraction peak and associated damped sine-wave-like oscillation in real space, “···is simply due to the fact that there is a fairly definite distance of closest approach of carbon atoms in different molecules,” and thus that little information about relative orientations of the chains or their conformations could be obtained. Bjørnhaug and colleagues, with the Norwegian Pulp and Paper Research Institute, extended real-space analysis to a wider array of synthetic and natural polymers including disordered cellulosic materials, polyvinyl acetate, poly(vinyl alcohol), polystyrene, polyhexamethylene adipamide, poly(methyl methacrylate), regenerated cellulose, and alginic acid–demonstrating that the real-space PDFs give distinct local structural fingerprints for different unoriented, weakly ordered, polymers.^96^ Real-space analyses would soon become a more refined tool to help understand the driving forces for local ordering in noncrystalline polymers. Kilian and Boueke^97^ demonstrated the importance and ubiquity of steric effects between phenyl substituents on the resulting macromolecular chain conformations for glassy polystyrene and related compounds. Wecker, Davidson, and Cohen^98^ determined that the molecular origins of features in the associated scattering patterns of atactic and isotactic polystyrene glasses correspond to interchain phenyl–phenyl and intrachain phenyl-to-main-chain correlations. Isotactic polystyrene was proposed to show wider experimental *d*-spacings, but higher density than atactic polystyrene, because more favorable packing of helically ordered phenyl group interactions led to an overall longer distance between the packing of main chains.^98^

Further work, typically with low real-space resolution, largely focused on identifying short-range ordering length-scales in various disordered, synthetic polymers (e.g., milled, molten, slow-cooled, or quenched disordered states),^99−103^ structure deterioration due to radiation-induced cross-linking,^104−106^ and copolymerization.^107,108^ Efforts to model structures based on the paracrystal theory of Rolf Hosemann^109^ were also developed,^100,103,110,111^ but while many of these studies yielded useful insights into processing effects on relative ordering states, many appear to have overinterpreted the medium-range ordering as a result of particular chain-packing orientations (e.g., short-range ordered parallel packing of linear segments), as shown by Mitchell, Lovell, and Windle,^112^ and confirming the earlier sentiment by Simard and Warren (see section 4.5). Studies using higher quality experimental data have further aided in accessing more accurate pictures of conformational states in the very local structures of noncrystalline polymers and polymer melts.^113−118^ These efforts have also benefitted from methods developed for fitting or comparing total scattering data in reciprocal space.^119−122^ A recent review further discusses the use of scattering for characterizing noncrystalline polymers.^121^

PDF studies were also applied to investigate charge transport processes in conductive polymers.^123^ Polyaniline (PANI) is a class of low density, easily processable, and relatively inexpensive conductive polymers that has potential applications in gas separations and organic electronics.^124,125^ The backbone is built from monomers in reduced and/or oxidized states, and each oxidation state can exist in the base or salt form by doping with protonic acids.^126^ Studies investigated the positions of bromine sites from doping into the emeraldine base form of PANI^127,128^ and local ordering in amorphous forms as a function of synthesis and processing (undoped–doped–dedoped samples).^129^ Decomposition of intra- and interchain contributions showed reversible changes in the intramolecular structure on doping/undoping from the as-cast base form while the interchain structure depends on its processing history, which correlates with enhanced selectivity in gas diffusion studies for the redoped PANI films.^130,131^

**Figure 5:**
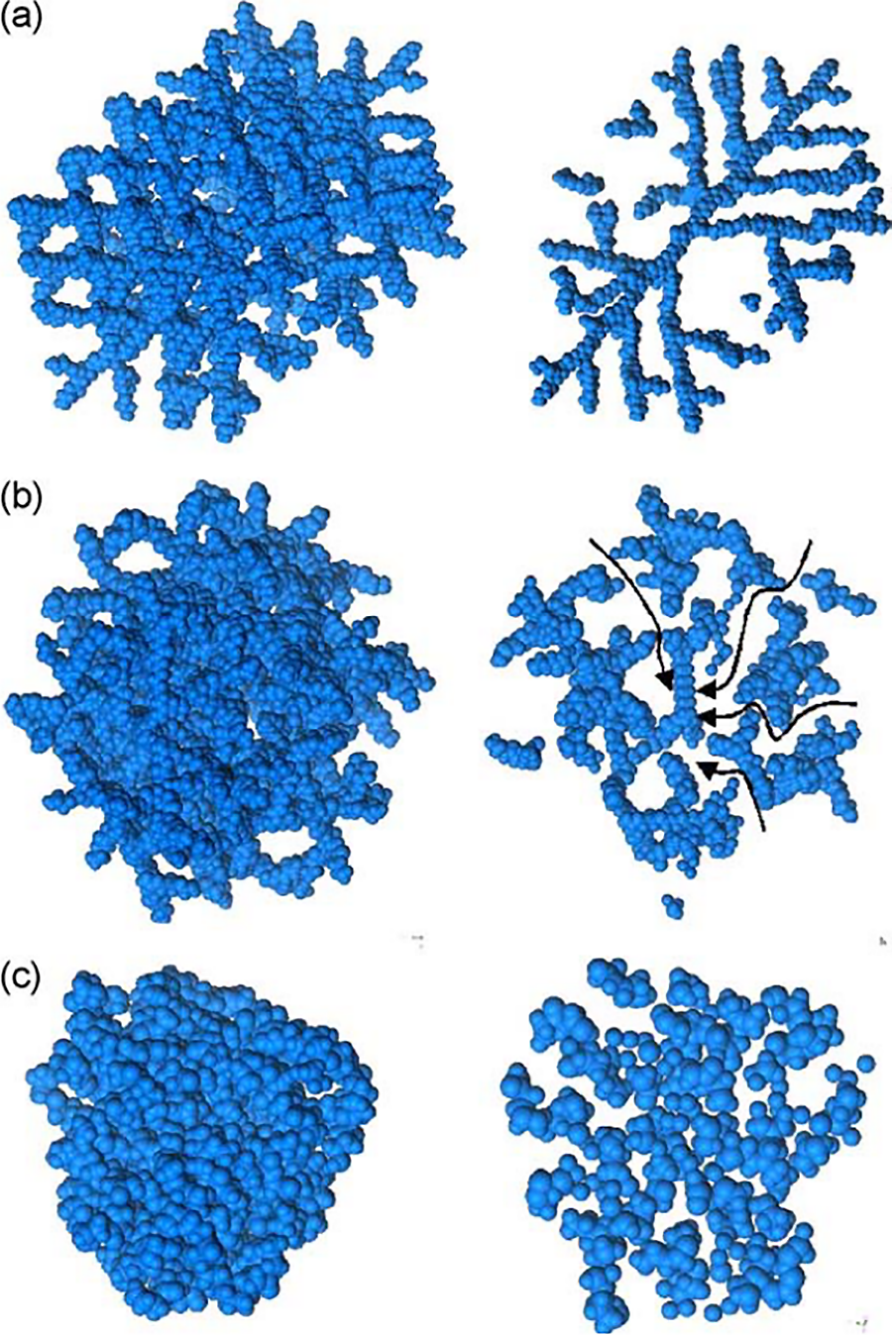
Complex hierarchical models generated from molecular simulations: different models proposed to interpret data from dendritic and hyperbranched poly(amidoamine) (PAMAM) macromolecules, reproduced with permission from ref (133). Copyright 2005 Elsevier.

The intermediate length-scale structuring of polymers that show nanoscale ordering can also be studied in real space, for example in the class of polymers called dendrimers. These are globular, monodisperse, repetitively branched macromolecules that radiate from a central core and have applications in drug delivery and as sensors.^132^ The local structures in dendritic and hyper-branched poly(amidoamine) (PAMAM) macromolecules were measured and compared to crystalline C_60_.^133^ Dendritic PAMAM showed increased atomic ordering of semiregular segments compared to more condensed, hyperbranched PAMAM. 3D atomic models built by successive attachment of amidoamine (CH_2_–CH_2_–CO–NH–CH_2_–CH_2_–NH_2_) monomer units to a diaminododecane core (C_12_H_28_N_2_) and subsequently relaxed using molecular dynamics (MD) force fields, Figure 5, could qualitatively reproduce the extent of ordering and some features of the experimental data. In cases such as this, a quantitative fit of the resulting models to the data could aid in further determining remaining inadequacies in the MD ensembles in describing both atomic and intermediate length-scale structuring.

Another important class of polymers is semicrystalline polymers, comprising partly ordered domains but with significant interfacial backfolding between ordered and amorphous domains that results in weakly crystalline, heterogeneous structures. In many cases, idealized crystal structures have been determined using fiber diffraction, but atomic-scale structural insights into unmodified and unoriented states are difficult by these methods. This information is, however, crucial for real-world industrial applications. A study of polyamide 6 (PA6) investigated samples produced through different synthetic procedures, including hydrolytic and anionic syntheses, and postprocessing treatments of annealing or foaming.^32^ Reliable measurements of atomic conformation details and lattice parameter values for nanocrystalline domains could be obtained from real-space refinement despite limited information available in *Q*-space for constraining Rietveld-style refinements. The interlayer spacing, domain size, and amorphous content were shown to be most affected by synthesis and treatment conditions, and the crystalline domains were shown to be significantly smaller than the typical spherulites observed by TEM. Scattering intensities often attributed to impurity γ PA6 were shown to arise from only short-range rather than long-range ordered structural states. These results suggest the utility for revisiting many semicrystalline polymers systems in the unoriented state using modern real-space methods. The effects of polymer chain translations, rotations, and individual atom adjustments on describing the structures within polytetrafluoroethylene (PTFE) and polychlorotrifluoroethylene (PCTFE) have also been investigated using empirical potential structure refinement (section 6.3).^134^

**Figure 6:**
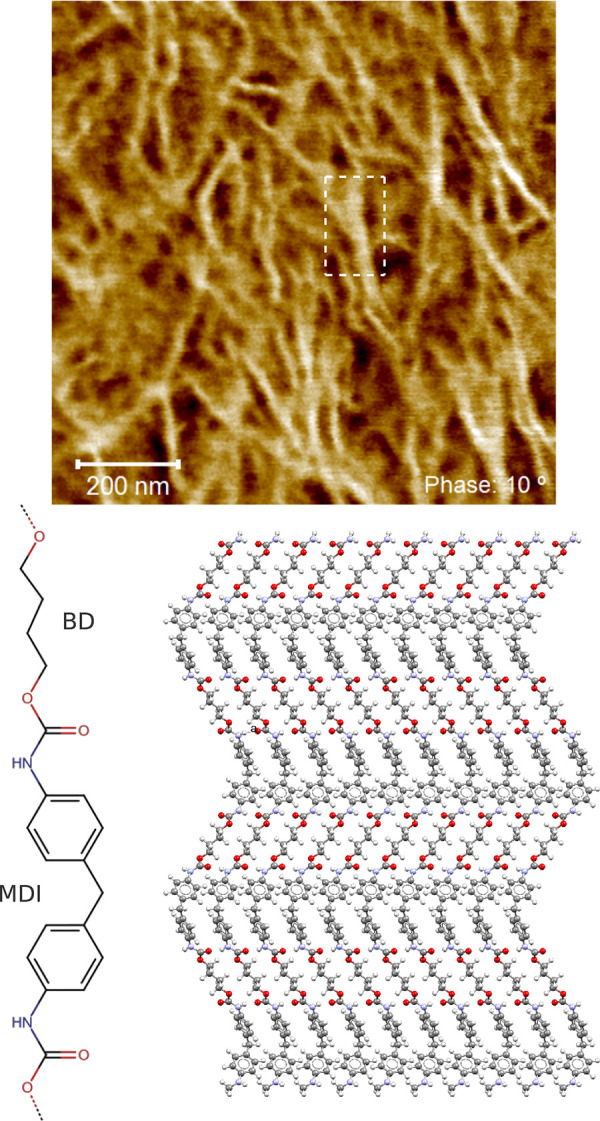
Atomic structure within nanosized domains: atomic force microscopy image^139^ of TPU hard phases (lighter regions) consisting of 4,4′-methylene diphenyl diisocyanate (MDI) and 1,4-butanediol (BD) within an ester-based soft segment matrix (darker regions). The MDI-BD hard segment is shown below along with a sequence of H-bonded chains from the model hard phase structure.^137^

Thermoplastic polyurethanes (TPUs) are a wide-ranging class of important, industrial elastomers. They are semicrystalline, block copolymers comprising microphase-separated soft and hard regions. Soft segments are composed of polyester or polyether chains that give the material its plasticity, while the hard segment domains are formed by the addition of a chain extending diol and a diisocyanate, and provide rigidity and elasticity through physical cross-linking.^135,136^ PDF investigations were applied to study the structural states of a series of TPU model hard phases with varying ratios of 4,4′-methylene diphenyl diisocyanate (MDI) and 1,4-butanediol (BD).^137^ At a ratio of 1:1 MDI:BD, the hard phase structure was amorphous. Two different nanocrystalline forms were observed upon increasing the ratio to values of 1:1.2, 1:1.5, and 1:2. A new model for the MDI-BD hard phase structure (Figure 6) was proposed with hydrogen bonding occurring along one direction perpendicular to the chain axis and the other packing direction stabilized by edge-to-face interactions between neighboring phenyl groups, in contrast to a previous model proposed by Blackwell et al.^138^ with H-bonding in both directions. At 1:2, the hard phase could be well described by the molecular packing motifs formed by unpolymerized MDI capped by butanol, which was supported by the lower molecular weight of the 1:2 polymer measured by gas phase chromatography.^137^ TPUs with different compositions of hard segments and soft segments (e.g., varying combinations of MDI-BD or HDI-BD and ether or ester components) form a wide range of nanocrystalline structural states and morphologies. The hard phase domains in a given sample are observed to exist in a distribution of both ordered and disordered states,^137,139^ which is likely related to the presence of a rigid amorphous fraction in semicrystalline thermoplastics,^140−142^ giving another target for further investigation.

#### 3.2.2 Biopolymers

Real space analyses have played a pivotal role in understanding biologically important macromolecular polymers as well. In addition to the measurements of cellulosic and alginic acid polymers mentioned above,^96^ early utility was demonstrated in the foundational work of Linus Pauling, Robert Corey, and Herman Branson in establishing the structure of the α-helix for polypeptides. Linus Pauling, Lawrence Brockway, and co-workers had already been using PDF analysis of electron diffraction data to build up libraries of accurate bond distances and angles for various molecular structures including those relating to amino acid building blocks of biopolymeric proteins.^143−146^ They would eventually demonstrate the agreement of the proposed 3.7-helix conformation^147,148^ with correlation functions extracted from the Patterson function of hemoglobin, in contrast to models proposed by William Bragg, John Kendrew, and Max Perutz.^149^ They suggested the likelihood that this polypeptide configuration is represented in other globular proteins.^148^ Further assessments investigated bovine serum albumin^150^ and the validity of such analysis for globular proteins in general.^151^

Benmore, Yarger, and co-workers investigated the structures of spider dragline silk fibers.^152^ Silks are extremely tough materials, typically considered to be a distribution of crystalline β-sheet-like structures within an amorphous matrix. Total scattering structure functions and corresponding differential PDFs were derived from slices along different directions of 2D diffraction patterns from fibers oriented both perpendicular and parallel to the incoming beam. The majority of real-space correlations consisted of weak ordering up to ∼1 nm, suggesting that the structure is dominated by orientated nanocrystalline and amorphous entities. Disordering of the molecular orientations of sheet or chain fragments along the fiber axis could be associated with the flexibility of the silk. The longer length-scale packing of β sheet crystals along the fibers was also investigated by pair distributions obtained from the small angle scattering (SAS) regime (see section 5.8).^153^

### 3.3 Small Molecules

#### 3.3.1 Pharmaceuticals

It it commonly noted that anywhere from 70 to 90% of drug candidates at various stages of development have low solubility.^154^ Extensive investigations into formulation engineering techniques have addressed these physical property limitations, leading to intense and widespread research thrusts into the use of salts, eutectics, cocrystals, nanoparticulates,^155^ and especially amorphous phases,^156,157^ for achieving suitable physical and pharmacokinetic properties. A large body of work has focused on the production and characterization of amorphous drug formulations.^158,159^ The noncrystalline nature creates many problems including the unique identification of the drug form for purposes of quality control and intellectual property litigation, quantitative analysis of the structure and/or phase composition, and stabilization for a suitable product shelf life.

Applications of PDF analysis toward amorphous pharmaceutical formulations have included pharmaceutical drugs prepared via milling,^160−165^ dehydration,^166^ compaction,^167^ and quench cooling.^168^ However, many of these studies neglected the lack of atomic-scale resolution and significant termination effects associated with the limited reciprocal space probed using low-energy Cu Kα radiation and low angular-range measurements,^169,170^ thus making it difficult to infer direct structural insights from the data. However, high quality data measured from both synchrotron X-ray, laboratory X-ray, and neutron total scattering experiments can and have been used to directly compare changes in intramolecular bond distances, intermolecular packing, and to track processing and aging induced changes of both amorphous and nanostructured pharmaceuticals.^171−174^ For instance, nanocrystalline domains of approximately 4.5 nm diameter of the antiepileptic drug carbamazepine form III could be identified in a melt-quenched formulation.^172^ PDF measurements also provide additional information for constraining accurate bond distances, intermolecular associations, and intramolecular thermal behavior in comparison to conventional methods for solution and refinement from powder diffraction.^175−177^ This may extend to solving structures in cases with limited crystallinity (see section 5.2 for further details) as well as obtaining information about molecular conformations and packing in the amorphous state.

Minority amorphous phases can be unintentionally generated during formulation processes, while minority crystalline phases can be left over from incomplete amorphization or partial recrystallization. Amorphous and crystalline components are also often intentionally mixed to modify the physical properties of the formulation,^178^ or for use in liquid suspension, making it important to identify and quantify these components. High flux scattering from synchrotron sources along with PDF analysis has been demonstrated to unambiguously identify pharmaceutical ingredient nanoparticles in aqueous suspension at concentrations as low as 0.25 wt %.^179^ This can be useful for characterizing solution-based drug products as used for instance in intravenous therapies or nebulizers. Further reduced concentrations may be assessed with sufficient particle crystallinity and suitable profile resolution.^180^ On the other hand, there are a variety of ways to quantify amorphous and crystalline phase content,^181^ but there is currently no broadly applicable standard technique. Phase quantification for amorphous and crystalline pharmaceuticals has been demonstrated using the PDF method for both single and multicomponent systems,^38,177,182−184^ which may extend particular benefit to characterizing materials containing multiple disordered phases (see section 5.3).

An issue with the use of amorphous formulations is that they are thermodynamically metastable and therefore tend to revert to thermodynamically stable forms (i.e., crystallize). This leads to unstable formulations that may not have a suitable shelf life for market product purposes. By mixing the active pharmaceutical ingredients with excipients, typically polymers or sugars, the combination of higher solubility and propensity to remain in a metastable state can be optimized. An important aspect of this process is in choosing the excipient material. The drug’s solubility within the excipient can determine whether the formulation remains suitably mixed or phase segregates. All the while, this processing must maintain (and sometimes enhance) the therapeutic action of the individual constituents and the intramolecular structural integrity of the drug. Numerous studies in the literature have focused on determining the degree of mixing in multicomponent dispersions,^185−193^ though again with limited data quality. The best examples of this application have been demonstrated by Benmore and co-workers in assessing the miscibility and interactions in drug–polymer mixtures. The antifungal drug itraconazole was studied in polyvinylpyrrolidone (PVP) or hypromellose phthalate (HPMCP) polymers,^194^ and amorphous solid dispersions of a breast-cancer treatment drug lapatinib in HPMCP and hypromellose (HPMC-E3) (see section 5.4 for more details).^195^ Other increasingly important applications for local characterization may include the loading of pharmaceutical agents in microporous carriers.^196^

Understanding crystallization,^197^ disordering mechanisms,^183^ and relaxation processes^198,199^ can further aid in understanding property changes associated with different processing and formulation procedures. Such analyses can be useful for determining issues related to compound stability during processing and comparing the viability of different processing methods. For example, the extent of crystallite structural disordering could be shown to correlate with the specific conditions of different milling and compaction procedures for brittle versus soft active pharmaceutical ingredients as determined by nanoindentation.^200^ Another study showed that amorphous and mesomorphous calcium ketoprofen show similar levels of local ordering, despite a more ordered lamellar structure in the mesomorphous form, while disordering of the crystalline dihydrate occurs from water loss and structural collapse upon both heating and melt quenching.^171^ In the opposite sense, environmental conditions that drive different nucleation behaviors are also interesting to stabilize compounds or crystallize particular polymorphs. An in situ study of paracetamol nucleation showed that there is a propensity for different intermolecular packing interactions just pior to crystallization from methanol versus 1-propanol, driving the formation of either monoclinic form I or orthorhombic form II, respectively.^197^

#### 3.3.2 Saccharides

Besides their use as sweeteners, saccharides (also carbohydrates or sugars) play an important role in biological processes and are widespread filling and binding agents in food and pharmaceutical formulations.^201^ Because they can form different stereoisomeric forms called anomers, display a plethora of means to forming favorable hydrogen bonding interactions, and are often hygroscopic, they tend to form many crystalline polymorphs, hydrates, and often glasses.^202^ Varying properties impact choices during both product development and end-product performance such as caking, hardness, flowability, compaction, solubility, stability, and sweetness.^178^ Thus, it is important to understand the product formation and modifications under different synthesis, processing, and storage conditions.

In a similar manner to the studies addressing pharmaceutical agents, total scattering and real-space analysis have been applied toward understanding these processing pathway effects on sugar structure and stability. Applications of high-energy total scattering toward low-*Z* materials were discussed in a 2013 article by Petkov et al.^203^ They demonstrated that poorly crystalline and amorphous glucosides including cellulose and trehalose show distinct local structuring with characteristics comparable to certain bulk crystalline forms. This would suggest a means to understanding the molecular interactions governing different sugar forms.

**Figure 7:**
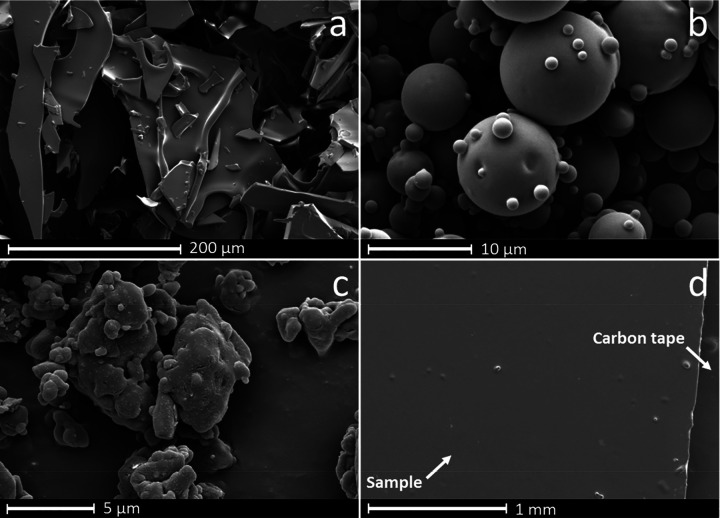
Differing morphologies for amorphous sucrose prepared through (a) freeze-drying, (b) spray drying, (c) ball milling, and (d) melt quenching. Figure reproduced with permission from ref (204). Copyright 2019 Elsevier.

Sugar compounds can be made into glasses through thermal-, mechanical-, and solution-based routes. However, not all forms are equal, displaying differences for example in morphology and thermal behavior. Notably, the latter leads to significant differences in the stability of the amorphous state against crystallization. Samples of amorphous lactose prepared by spray-drying, lyophilization, and melt-quenching were studied at increasing time spent under accelerated aging conditions.^38^ Substantial deviation in the local structure of the melt-quenched sample compared to spray-dried and lyophilized samples was observed, which correlated with their respective tendencies to remain stable or crystallize to the monohydrate crystalline form under aging. Though local structure differences were observed, and a lack of long-range ordering signal appeared to rule out significant differences in the presence of preformed crystal nuclei, other root causes of this effect such as differences in moisture content or impurities could not be ruled out. More detailed studies were undertaken on the physicochemical properties and local structures of sucrose as a function of amorphization pathway.^204^ Different processing mechanisms lead to differences in, for example, thermal behavior, and morphologies as shown in Figure 7. A local structural modification was distinctly observed for stable, melt-quenched sucrose. This was identified as coming from a modulation in the on-average intermolecular packing between cyclic structures. Despite some differences in moisture content, the modification correlated most with a higher concentration of thermal decomposition indicator compounds such as glucose, fructose, 5-(hydroxymethyl)furfural, and 1-kestose identified by high-performance liquid chromatography and anion-exchange chromatography with pulsed amperometric detection. Melt-quenched sucrose was postlyophilized and compared to mixed sucrose/glucose/fructose,^205^ and a followup study confirmed that degradation impurities modify the packing of sucrose molecules and contribute to inhibiting crystallization.

The amorphization mechanism was studied by Bordet et al.^183^ for β-trehalose using high energy milling. By tracking simultaneously the amorphous/crystalline phase fraction and the coherence length of the crystalline domains, they described amorphization as a two-part process through which amorphous layers form and thicken around crystalline cores, and there is a progressive increase of defect density that reduces the coherence length of the ordered domains within the core. Rigidity of the glucose units up to 4 Å and conformational disordering of the glycosidic linkage between ball-milled and melt-quenched β-trehalose were observed.^183^ Analysis of further molecular packing shells and composition analyses were not undertaken as with sucrose,^204,205^ though further experimental investigation is warranted regarding the development and effects of impurities on the stability of the thermally accessed versus mechanically accessed forms.

Because of their hygroscopic properties and functions in aqueous systems, the study of interactions between water and sugars is also a fundamental area concerning issues from the development of the glassy state from solution-based processes to the perceived taste of sugar. For example, neutron diffraction studies coupled with local structure analysis have shown that the strength of hydrogen-bonding interactions and amount of water in the sugar hydration shells affect how the molecules can interact with taste receptors, and therefore the sweetness.^206−208^ The sweetness favors stronger hydrogen-bonding interactions and increased hydration states, possibly indicating that water mediates binding with sugar-receptors. Related to the studies of sugar-water interactions is the role of sugars, notably trehalose, to bioprotection or the stabilization of biomacromolecules such as proteins, lipids, and DNA against environmental stressors in vivo. Studies have shown that trehalose forms relatively weak hydrogen bonds with water with a minor impact on the structure of solution water versus bulk water,^207,209,210^ therefore preferring trehalose–trehalose accumulation and vitrification,^210,211^ possibly around biomolecules, as implied by the protection mechanism.

#### 3.3.3 Water and Aqueous Solutions

Water plays a prominent role in almost all aspects of our lives. An understanding of the local arrangement and interactions of water molecules is critical to understand the complex phase behavior and properties of water and the various phases of ice. Total scattering measurements and PDF analysis are some ways of testing models of structural states and interaction potentials for liquid and amorphous water phases. Measurements as early as the 1930s by Katzoff^212^ and Morgan and Warren,^213^ already demonstrated a tetrahedral-like (though imperfectly so) nature of liquid water with primary O–O distances centered around ∼2.8 and 4.5 Å. Measurements of water were further involved in significant advances in experimental data collection, reduction, and interpretation, especially in the works of Alfred Narten^214−218^ and later Alan Soper and co-workers.^219−223^ A ground-breaking turn in this effort was the development of empirical potential structure refinement (EPSR), which allows complex atomistic models to be applied to study complex liquids such as water without overfitting by using a small number of empirical potential parameters as variables (see section 6.3 for more details).^224^

**Figure 8:**
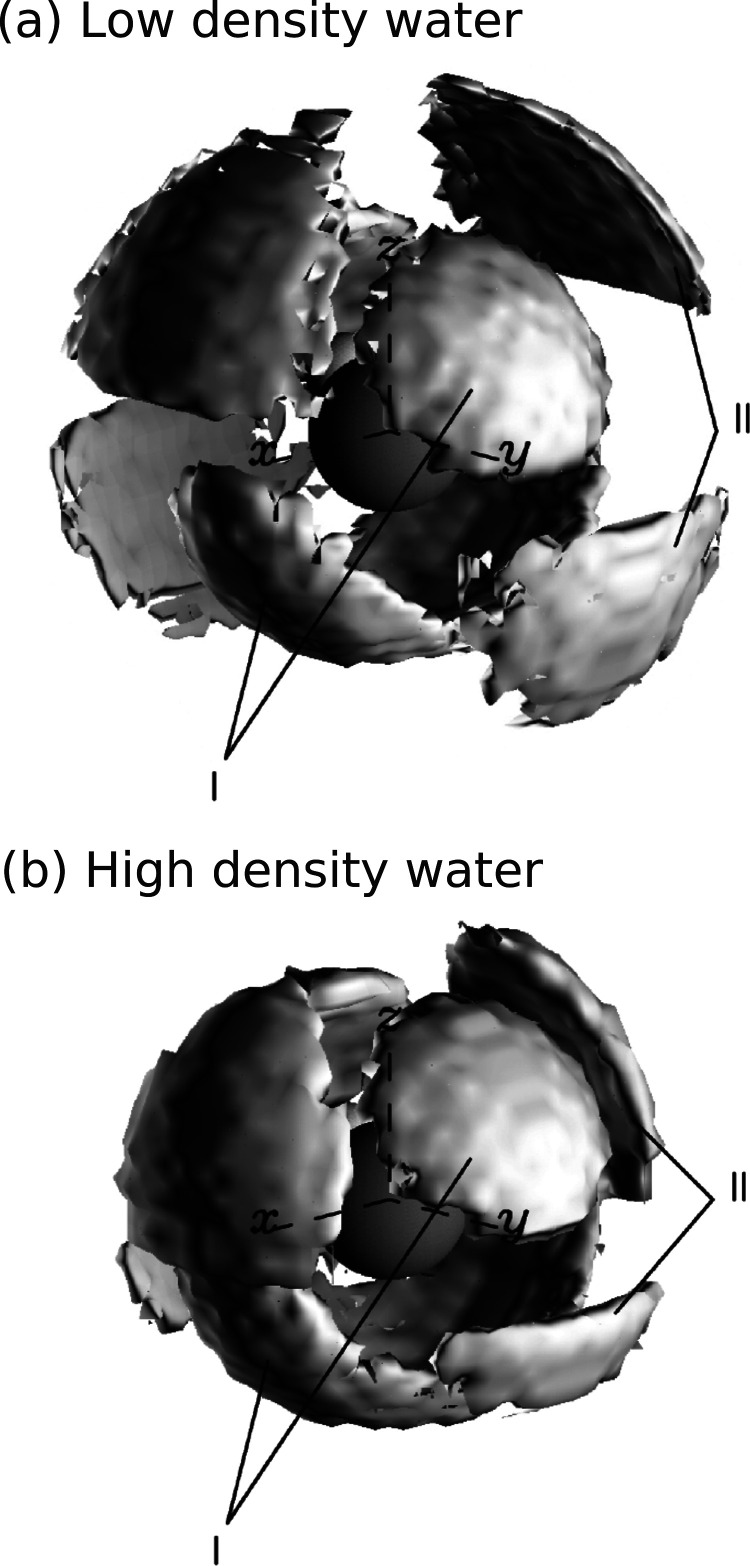
Water coordination: spatial density functions for (a) low and (b) high density water as determined from EPSR simulations, plotted in a 10 Å × 10 Å window, with a central water molecule lying in the *zy* plane. Lobes of density (I) are observed opposite each OH vector on the central molecule and in a broad band of density at right angles to these underneath the central molecule, corresponding to the first shell of approximately tetrahedrally bonded water molecules. A second shell (II), out of phase with the first shell, is observed to collapse going from low to high density water, merging with the first shell near the *x*-axis. Reproduced with permission from ref (238). Copyright 2000 American Physical Society.

The microscopic nature of water has been a very contentious topic in the literature with a variety of models proposed including heterogeneous mixtures of different local structures.^225−228^ Ambient water is considered to form a distributed hydrogen-bonding network with tetrahedral-like local coordinations.^229,230^ That said, O–O coordinations slightly higher than expected for perfect tetrahedral coordination (i.e., between 4–5 neighbors), is generally reported for liquid water. This may be thought of as being due to occupied interstitial positions, which helps explain the higher density of liquid water than crystalline ice.^231^ Variations in coordination shell numbers, O–O–O bond angles, and the extent of structural order are observed as a function of temperature^221,232−234^ and pressure,^235,236^ leading to low- and high-density liquid states^237−239^ (Figure 8) as well as different metastable forms of amorphous ice^240−247^

As already discussed for sugars, a detailed understanding of the structure of water is necessary as a foundation on which to understand the complex interactions provided by water when operating as a medium for other chemical species. The propensities for self- or intermolecular associations of organic molecules in aqueous and ionic solutions are fundamental to many physiological processes such as membrane permeability and protein folding, aggregation, denaturation, or stabilization. Using isotopic substitution neutron-scattering measurements (see section 7.1.2) combined with EPSR, some clues have been gleaned into how the incorporation of urea into water may affect the solution’s ability to dissolve alkanes, or urea’s role as a denaturant.^248^ Trimethylamine *N*-oxide was shown to interact directly with urea, but its role in counteracting denaturation at physiological concentrations may have more to do with the mediating effects of water.^249,250^ Similar studies have investigated interactions in aqueous solutions of dimethyl sulfoxide,^251^ proline and related peptides,^252^ glutamine and polyglutamine,^253,254^ glutathione,^255^ indole,^256^ aspartic acid,^257^ propylene glycol,^258^ phosphocholine,^259,260^ and 1,2-dioleoyl-*sn*-glycero-3-phosphoethanolamine (DOPE).^261^

Despite their miscibility, mixtures of water and alcohols over varying concentrations have been observed to prefer the formation of molecular clustering and bipercolating networks.^262−265^ These molecular-level heterogeneities were suggested as a possible origin of mixture properties deviating from ideality, favored over the competing model of the restructuring of water for instance. Such interactions could also play a role in the mediation of flavor-enhancing amphipathic molecules in the dilution of whisky,^266^ and may warrant neutron diffraction experiments on Scotch.

Beyond biological implications,^267^ ion solvation is important in topics such as electrochemistry or the physical properties of groundwaters.^268^ Various methods^269^ have been used to extract signals of ion solvation shell formation in different electrolyte solutions including, for example, a wide variety of alkali,^270−273^ alkaline-earth,^274^ transition metal,^270^ and lanthanide^275,276^ chlorides, and various potassium halides.^277^ With respect to their geophysical roles in both terrestrial and even Martian environments, molecular anions including sulfate and perchlorate solvation have been investigated.^278−280^ PDFs were also recently used to identify the formation of adsorbed and hydrated layers around nanoparticles in suspension.^281^ While not discussed further here, it it worth noting that an additionally large body of work has also focused on the molecular states in other liquids^15,282,283^ such as hydrocarbons,^284−292^ their derivatives,^293−298^ ionic liquids,^299−303^ and acids.^304,305^

### 3.4 Materials with Molecular Components

#### 3.4.1 Coordination Compounds and Metal–Organic Frameworks

Porous coordination compounds and metal–organic frameworks (MOFs) are a broad class of materials typically consisting of metal ions or clusters interconnected by strongly bonded associations with organic molecules, providing a plethora of topologies, geometries, and functionalities.^306−308^ They have become a major forefront in materials design and synthesis, especially since the 1990s and early 2000s, utilizing the concepts of coordination chemistry, framework solids, and reticular synthesis.^309−313^ This is an extremely broad classification, encompassing a rich variety of subcomponent chemistries and wide-ranging, potential applications in gas storage, gas/vapor separation, catalysis, magnets, sensors, drug delivery and more. Industrialization efforts have centered primarily on gas capture and release applications such as controlled fruit ripening, toxic gas storage, and water harvesting.^314,315^

MOFs have been typically characterized as a class of crystalline coordination compounds with ordered pore structures. However, added degrees of freedom granted by the building block occupancies, orientations, connectivity, and flexibility lead to complex local structural effects responsible for many intriguing macroscopic properties such as negative thermal expansion (NTE), significant structural flexibility (often termed breathing), and disordered intermediates during processing. The use of PDF specifically for MOFs has recently been reviewed by Castillo-Blas et al.^316^

**Figure 9:**
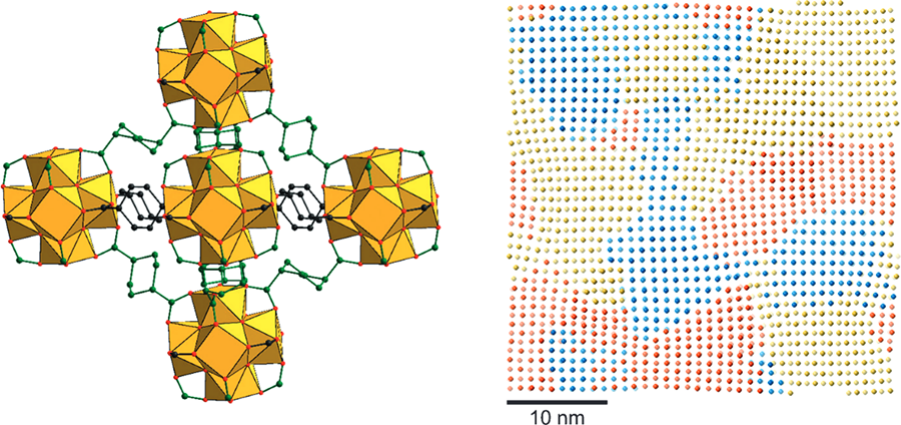
Local domain formation in metal–organic frameworks: evacuation-induced linker contractions lead to tetragonal nanodomains, reproduced with permission from ref (319). Copyright 2016 John Wiley and Sons.

Framework flexibility and defects have become a prominent topic of discussion and a prime target for local structure investigations. The presence of low temperature static disorder within zinc(II) isonicotinate was investigated to track translation, rotation, and distortion components of the bond angles within the ZnN_2_O_2_ tetrahedra and within the X–Zn–X (X ≡ ligand center-of-mass).^317^ The framework flexibility could be explained by large transverse displacements of the ligands governed by displacements of neighboring Zn sites and correlated bending of the intermediating linker. The rotations of phenyl groups were investigated in sodium *p*-chlorobenzenesulfonate, although the poor agreement factors suggest that the models could not give a good description to the local structure as tested.^318^ Conformational flexibility of linkers has been implicated in complex breathing behaviors, for example *trans*-1,4-cyclohexanedicarboxylic acid linkers in an aliphatic analogue of UiO-66(Zr).^319^ The removal of adsorbed content results in locally oriented nanodomains of tetragonal-type distortions of the nodes, driven by contraction of approximately one-third of the linkers, Figure 9. Framework flexibility has likewise been shown to aid in reversible bonding rearrangements induced by both hydration and dehydration mechanisms^320,321^

The presence of vacancies in many MOF systems has become well-known. Consistent Hf_6_ intercluster correlations on increasing terephthalate linker vacancies were identified in UiO-66(Hf), helping to elucidate the presence of Schottky-like linker–cluster vacancies that are correlated over ∼7 nm, Figure 9.^322^ Controlled incorporation of such defects has been subsequently demonstrated for tuning the magnitude of NTE observed.^323^ The observation of linker vacancies in the porphyrinic MOF PCN-221 was also observed to coincide with orientational disordering of Zr_6_ clusters. Such behavior could open a new level for controlling the amount and type of coordinatively unsaturated sites open for interaction with guest species.^324^ Localized distortions of the clusters themselves, separate from the overall framework symmetry, have been demonstrated for example in Hf and Zr oxide nodes induced by both rising temperature and site-isolated binding effects.^325,326^

Amorphous MOFs now make up an emerging subclass of the typically crystalline MOFs with potential applications in toxic/radioactive waste separation and containment. While amorphous MOFs had appeared as undesired products or intermediates, they have gained interest for applications in gas separation, guest trapping, ion transport, or sensors for instance by reversible changes in luminescent or electrical conductivity on amorphization and crystallization.^327^ Detailed structural investigations, due in large part to the works of Bennett, Cheetham, and co-workers, have shown that amorphous zeolitic imidazolate frameworks (ZIFs) can form continuous random networks analogous to amorphous silica.^328^ Further work has explored the structural states of, pathways to, and interrelationships between amorphous and liquid MOF states,^329−336^ opening the door to new composite materials and blends.^337−339^ Structural changes associated with interesting behaviors have been characterized including changes in insulating to semiconducting transitions,^321^ and polyamorphism of amorphous intermediate structures.^340^ Disordered metal–organic networks have also been shown to successfully incoporate Co_4_O_4_ cubane catalysts for the oxygen evolution reaction with improved stability over their use in solution.^341^ Investigations into the nucleation of MOFs from solution have also given insights into the prenucleation species and disordered intermediates involved in forming ultimately crystalline structures in ZIF-8^342^ and UiO-66(Zr).^343^

Besides the structural states of the frameworks and their components, real-space studies have flourished in helping to verify and elucidate the mechanisms of functionalization processes. PDFs are uniquely sensitive for identifying the presence, site-isolation, and structural states of species added into the pores and/or bound to coordinatively unsaturated sites on the clusters (see section 5.6 for more details), for instance by gas and liquid adsorption,^344−353^ atomic layer deposition,^326,354−356^ ligand exchange,^357^ nanocasting,^358^ and the incorporation or nucleation of larger complexes and nanoparticles.^356,359−365^ These processes will be especially important for enabling applications in gas loading/storage, remediation, catalysis, and more. Further effects on the structural state of the framework have been investigated due to electrochemical cycling for battery applications^366^ and degradation,^367,368^ and due to in situ loading and detoxification of chemical warfare agent simulants.^369^

#### 3.4.2 Layered frameworks

Layered framework materials constitute another large class of microporous materials with a similarly wide range of potential applications. Porosity can be manifested through tailored spacing of layered motifs using molecular pillars or through synthesis of molecularly precise layers that stack to form ordered channels running perpendicular to the layers. An example of the former includes so-called unconventional metal–organic frameworks (UMOFs), which can be pre- or postsynthetically functionalized and have applications as ion-exchange materials, proton conductors, catalysts, and sorbents.^370^ UMOFs comprise primarily layered zirconium, tin, or aluminum phosphonates, which form into pillared structures with porosity controlled through intermediating organic linkers. These compounds have been shown to form highly turbostratic (see section 4.6) nanocrystalline layers with weak stacking correlations.^371^ Open sites between the inorganic layers and coordinated by phosphate oxygens provide ion-exchange sites for separating lanthanide ions (L^3+^) from actinide ions (A^3+^) in spent nuclear fuel rods,^372^ by taking advantage of their affinity for more positively charged L^3+^ over oxidized actinide ions AO_2_^+^.

**Figure 10:**
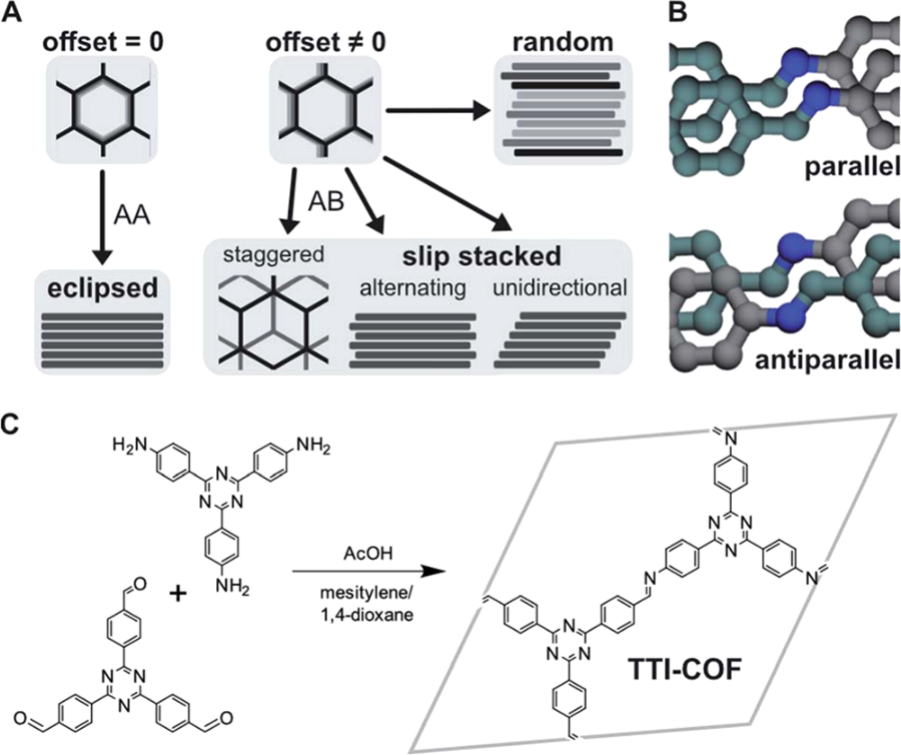
Different potential stacking modes in 2D COFs: (A) no offset, ordered offset, and random offset stacking, (B) parallel versus antiparallel directions of imine bonds, and (C) layer formation by condensation of aldehyde and amine components. Reproduced with permission from ref (376) under CC BY 3.0 license.

Covalent organic frameworks (COFs) are a class of porous organic polymers that can be synthetically controlled by polymerization of judiciously preselected building blocks.^373^ Though they can form both 3D and 2D structural motifs, the particular application of local structure analysis has gained recent interest for the 2D layered structures. Although energetic studies have shown that the stacking of polymerized layers should prefer slight offsets,^374,375^ a large number of studies have continued to identify hexagonal systems from diffraction, suggesting eclipsed stacking motifs. Pütz et al.^376^ used PDF analysis to show that the local stacking behavior in an apparent high-symmetry form of triphenyl triazine imine COF (TTI-COF) synthesized at room temperature actually shows local interlayer offsets of about 1.6 Å on average, similar to an ordered slip-stacked form and in agreement with the previous theoretical calculations. The apparent high symmetry results from an averaging of the randomized slipping direction from layer to layer. For example, different types of stacking are displayed in Figure 10. Similar behavior was also observed in layered poly(heptazine imide) (PHI) compounds, where water-coordinated potassium loading in the pores led to a structure-directing effect and more ordered stacking, while proton-exchange led to a more random ordering of the layers.^377^ The synthesis and subsequent local structure assessment of a silicate covalent organic framework (SiCOF) have suggested a predominantly eclipsed stacking motif.^378^ It is plausible that the twisted orientations of the linkers could stabilize more preferential interactions between the aromatic moieties, though the lack of distinct interlayer correlations still suggests the presence of disorder in the stacking direction, and further quantitative modeling of these materials would be useful to better determine the nature of these interactions. Though COFs are primarily defined by their crystalline porosity, similar to MOFs, it is clear that disorder can actually be a fundamental component of their makeup. In fact, disordered COFs have been demonstrated through the reduction of amine-linked rPI-3-COF with formic acid, which leads to conformational distortion and increased stacking offsets, drastically reducing pore accessibility.^379^ Crystalline COFs were also shown to form via disordered intermediate amide-bonding networks.^380^

The incorporation of catalytic species within COFs has also been investigated. The interactions and catalytic behavior of palladium complexes loaded onto defect sites within imine-based COFs synthesized from 1,3,5-tris(4-aminophenyl)benzene (TAPB) and 1,3,5-benzenetricarbaldehyde (BTCA) was studied.^381^ After the catalytic Suzuki–Miyaura reaction, Pd^0^ nanocrystals approximately 3–4 nm in diameter could be identified in addition to well-defined Pd^II^Br_2_ sites resulting from the exchange of labile Cl for Br during the catalytic cycle. Similar assessment was made for Pt^II^ complexes, indicating aggregation of Pt during catalysis of the complexes in solution, but site isolation within the COF.^382^

Many other layered or 2D materials show complex structures and significant disorder. One example is transition metal carbides/nitrides encompassing a class of materials called MXenes.^383^ PDF studies have aided in the determination of layer composition and stacking, the effects of surface functional groups such as hydroxyl groups that can influence the nature of interlayer packing and intercalation properties,^384−388^ and the formation of MXene–organic heterointerfaces using small organic molecules.^389^ Investigation of other complex layered structures have included turbostratic xerogel slabs^390^ with intercalated or nanocomposite variants.^391,392^

#### 3.4.3 Thiophosphate Electrolytes

Sulfur and phosphorus form complex structures with various discrete, chain, and layered motifs. Investigations using PDF techniques have aided in the characterization of elemental sulfur S_8_ in crystalline,^393−395^ plastic,^394−397^ and liquid^394−405^ states. Phosphorus is known to display a rich allotropic phase space and different liquid and glassy states, which have also been studied by PDF,^406−411^ and may be further bolstered by improved atomistic simulation methods.^412^

Of particular technological interest is the combination of sulfur and phosphorus within thiophosphate solid electrolytes for applications toward solid-state batteries.^413^ With this comes the need to understand ion conduction mechanisms and thus the effects of the molecular anion interactions on the cation diffusion pathways. PDF data have been used to support a predominance of planar arrangements of [P_2_S_6_]^4–^ in the local structure of Li_4_P_2_S_6_,^414^ as observed for the Na_4_P_2_S_6_ analogue.^415^ Features left over in the difference, and changes in the goodness-of-fit by only a few percent between different models, suggest that contributions from stacking faults (see section 4.6) are probably needed to resolve the disorder of the P_2_ dumbell sites observed in the average structure. The presence of local orientational distortions have been suggested of [PS_4_]^3–^ anions in argyrodite-type Li_6_PS_5_Cl^416^ and Na_3_PS_4_.^417^ A number of recent studies have begun investigating the local structures of glasses prepared by mechanical milling of (Li_2_S)_*x*_(P_2_S_5_)_1–*x*_^414,418,419^ and (Na_2_S)_*x*_(P_2_S_5_)_1–*x*_,^420^ and the subsequent crystallization behavior. It is interesting that the glassy materials in these different studies show significant structural similarities, despite the wide variation in molecular anion species, for example, [PS_4_]^3–^, [P_2_S_7_]^4–^, [P_2_S_6_]^4–^, and [PS_3_]^−^ varying with the ratio of input Li_2_S, as measured spectroscopically.^421^ The local structure of glassy KSnPS_4_ was also compared to its crystalline counterpart.^422^ An ongoing challenge is to better understand the nature of these disordered phases, including the distributions of, and possible structural relationships between, the different molecular species. It is worth noting that the models of the intramolecular peak widths often applied to these materials are inaccurate, and improved benchmarking and treatment of these features (see section 4.3) is necessary for a more accurate extraction of any disordered phases, identification of impurity anion species, or for example, more quantitative characterization of composite mixtures.^423^

New compositions on the phosphorus–sulfur phase diagram are still being discovered, for example, a recent amorphous compound with the composition P_2_S.^424^ This study used a wide range of spectroscopic techniques and an assessment of a large set of theoretical and experimental structure models against the experimental PDF to determine local structural properties of the compound, finding that it showed similarities to the interconnected rings and bicyclic structures observed in violet phosphorus and P_4_S_3_, but could not be described as a simple mixture of the two different phases.

#### 3.4.4 Hybrid Organic–Inorganic Perovskites

Hybrid perovskites with the formula ABX_3_ (with A, B, and X being organic or inorganic ions) are highly promising photovoltaic materials, which have seen rapidly rising power conversion efficiencies over the past years.^425,426^ There has been a massive effort to understand the favorable charge transport properties of these materials, in spite of their mechanical softness compared to other typical solar-cell materials. Most PDF studies of these materials have focused on identifying dynamic local distortions^427−430^ and formation pathways of the inorganic octahedra,^431^ mostly because it is difficult to see the organic components among heavily scattering elements such as Pb and I with X-rays. The use of neutrons, however, can give greater sensitivity to the organic ions.^432−434^ A study of methylammonium lead bromide showed that the local structure of the low temperature orthorhombic phase was essentially maintained on heating from 5–300 K, suggesting that the hydrogen bonding associations of the methylammonium ions (CH_3_NH_3_^+^) with the Br^–^ ions are maintained in the instantaneous local structure, whether static at low temperature or configurationally degenerate at higher temperatures.^433^ Likewise, the formation of a feature at ∼3.7 Å on cooling formamidinium lead iodide could be associated with hydrogen bonding N–I pairs, suggesting increased association of the formamidinium ion (CH[NH_2_]_2_^+^) and inorganic cage with the slowing of the molecular motions.^434^

## 4 Real-Space Structure Characteristics

We now switch from reviews of scientific applications of PDF methods for molecular systems, to more specific discussions of the PDF analysis and its methodology.

### 4.1 Structural Order and Domains

Structural order can be thought of in terms of the spatial relationship between separate regions of atomic density in a material. If we sit on an atom, can we predict where other atoms will be, and over what distance will our predictions remain accurate? In a perfect crystal, the location of every other atom is known exactly, while in disordered materials, there is some uncertainty that decreases the accuracy of our prediction with increasing distance.

Short-, medium-, and long-range order (SRO, MRO, LRO) are used to characterize both the length-scale over which distinct spatial relationships between atoms exist and the types of interactions that participate over those distances.^162,435^ In molecular systems, SRO can be thought of as atoms that are directly, or indirectly bonded within a small molecule or subunit of a larger molecule. MRO is associated with the conformation of larger, torsionally flexible molecules and the local interactions with their neighbors. LRO is then related to the ordering of molecules over longer distances, which may or may not resemble a periodic packing. Bates et al.^162^ neatly discussed how the symmetries present, along with the degree of order, can be used to define the material class, for instance by translational, orientational, and conformational symmetry operators. Crystals contain all three operators; mesophases such as liquid crystals, condis crystals, and plastic crystals have one or two of the long-range symmetry operators, and amorphous states have none. Different examples of disorder are demonstrated.

**Figure 11:**
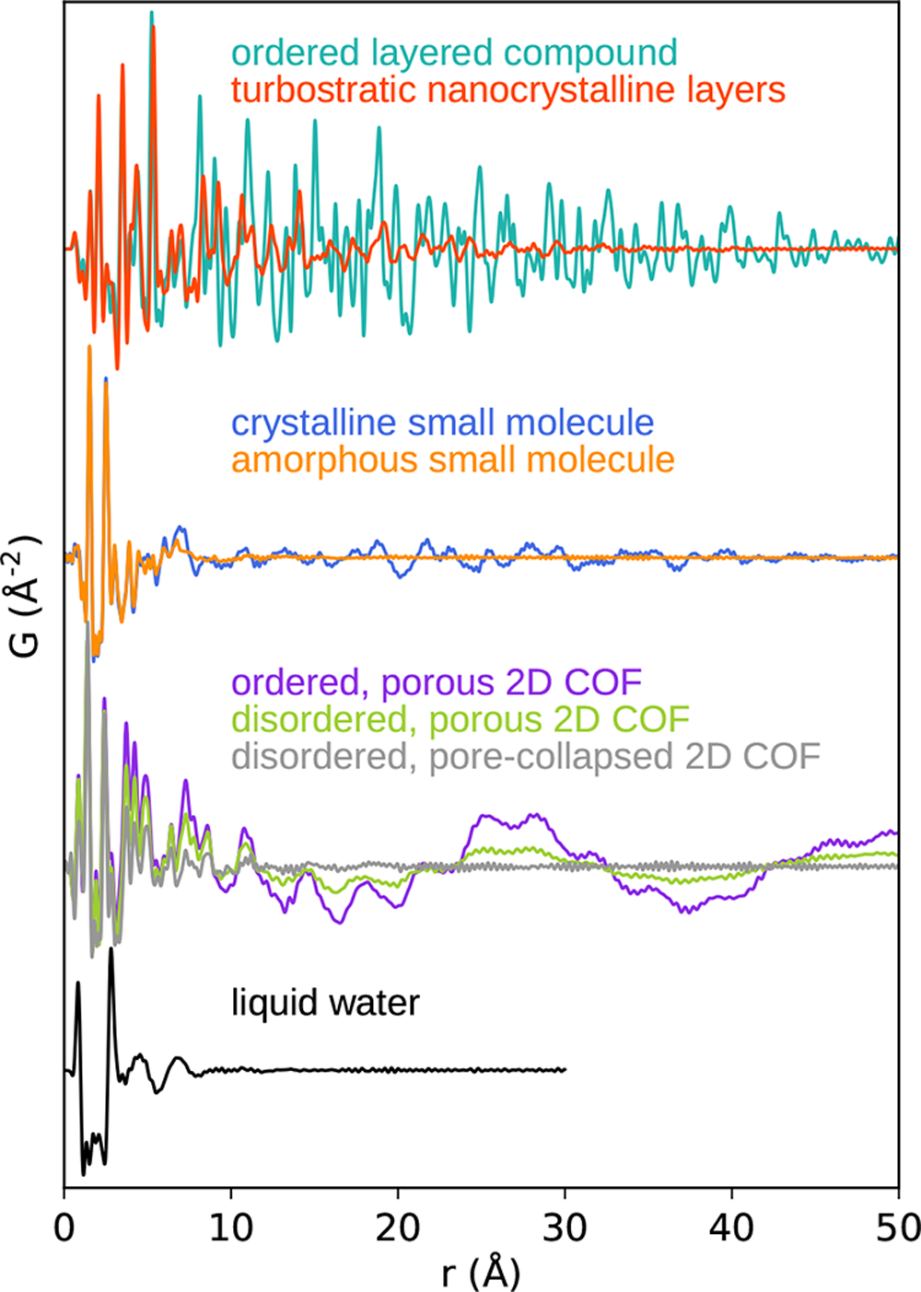
Examples of PDFs for ordered and disordered materials: (top) turbostratic, nanocrystalline layers of Zr-phosphonate-phosphate UMOF versus crystalline layered α-Zr-phosphate hydrate,^371^ (top, middle) crystalline versus amorphous small molecule HIV drug,^177^ and (bottom, middle) ordered slip-stacked TTI-COF and disordered TTI-COF with ordered porous channels^376^ versus disordered rPI-3-COF with collapsed pores,^379^ and (bottom) liquid water.^342^

The degree of structural order can be studied through the damping properties of the PDF signal. The simplest method is to determine the average structural coherence length (often referred to as crystallite or domain size). This is a measure of the maximum distance over which a distinct spatial relationship exists between atoms, after averaging over all domains in the sample. In the PDF, it can be observed as the distance where peaks in the signal damp to zero. The relative degree of order between samples can be studied by comparing damping behavior, and the presence or absence of associated changes, with things such as sample composition or processing conditions. Examples of PDFs for ordered and disordered versions of different materials are compared in Figure 11. A caveat is that when the order is long ranged, this distance may not be measurable, because the signal is also damped by effects due to the *Q*-resolution of the scattering experiment (see section 7.7).^32,139,177,436−439^ If the crystallite domain size, ξ ≫ 2π/Δ*Q*, where Δ*Q* is the *Q*-resolution of the measurement, the signal damping is dominated by resolution effects, and any quantitative estimate of the domain size becomes unreliable. It is important to consider this aspect when making such determinations. In a typical PDF experiment at a synchrotron using a 2D detector (rapid acquisition PDF (RAPDF) geometry,^437^ see section 7.2.1) this will apply to domains larger than 10 nm or so, and a separate experiment with higher *Q*-resolution should be performed for accurate domain size characterization. For smaller domains, quantitative information about the size and shape of the average domain can be extracted from the form of the envelope attenuating the signal. This envelope function is the spherically averaged autocorrelation of the domain and can be accounted for by multiplying a reference PDF simulated by a crystal structure model by a characteristic function γ(*r*)^33,440−442^ to damp the LRO signal,
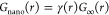
10reflecting the expected attenuation with increasing-*r*. The reference PDF may come from a measurement if that sample can be assumed as both relatively highly ordered and of the same atomic structure.^172^ It is often sufficient to assume spherically shaped domains for which the analytical expression is described by
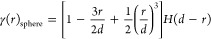
11with domain diameter *d*, and a step function *H*(*r*) with value 1 for *r* ≤ *d* and 0 beyond.^440,442−444^ Analytical expressions for γ(*r*) have been published for many other simple geometries including prolate and oblate spheroids,^445,446^ spherical shells,^442,446^ nanorods or nanotubes,^442,446^ nanosheets,^442^ and spherical distributions including log-normal spherical distributions.^444,447^ Usher et al.^448^ used a systematic parametrization procedure to develop numerical approximations for describing both simple and more complex shapes including spheres, octahedrons, cubes, tetrahedrons, rods, flat plates, and tetrapods.

Samples often do not consist of identically shaped or monodisperse particles. This can make it difficult to distinguish between different shape models by PDF modeling alone. However, if information is available from a complementary technique such as electron microscopy, a more suitable geometry may be chosen.^33,447,449,450^ If the small-angle scattering signal is measured, this can be used directly to obtain the proper form of the average domain shape envelope for the corresponding PDF.^37,451^

As particle size is reduced and surface-to-volume ratio increases, or in cases in which particles are highly anisotropic along certain crystallographic directions, these spherically averaged envelope functions may no longer be a suitable approximation. The approximation used in eq 10 assumes that the internal structure and the particle shape are separable. However, if the shape is strongly determined by the internal symmetries of the structure, then this is no longer true. For example, a sample may be rod-shaped specifically because of a preferential growth direction in the structure. In such cases, it becomes necessary to use structure models that simulate the whole cluster or domain to accurately describe the relationship between the atomic structure and morphology, which results in correlation between the damping properties and relative peak intensities in real space. This has been discussed extensively for inorganic nanoparticles^452−460^ and layered nanomaterials.^371,386,387,449,461,462^ To our knowledge, this has yet to be applied in a similar way to small molecule or organic nanocrystals. One benefit of decreased domain size, in the limit of very small domains or for amorphous matter, is that it becomes possible to directly observe the on-average size of the coherent structural order.^139,342,463^

### 4.2 Molecular Conformation

Different intermolecular packings (polymorphic or amorphous forms) can develop from the same molecule, and so it is useful to consider the effects of the individual molecule on the scattering and resulting PDF. Considering the coherent scattering intensities to come from either intramolecular or intermolecular interferences, the equation for the reduced total scattering structure function can be reformulated in terms of atoms *i* and *j* located within molecules α and β, as in
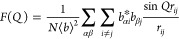
12where α = β represents intramolecular pairs and α ≠ β represents intermolecular pairs, or more simply
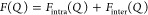
13

**Figure 12:**
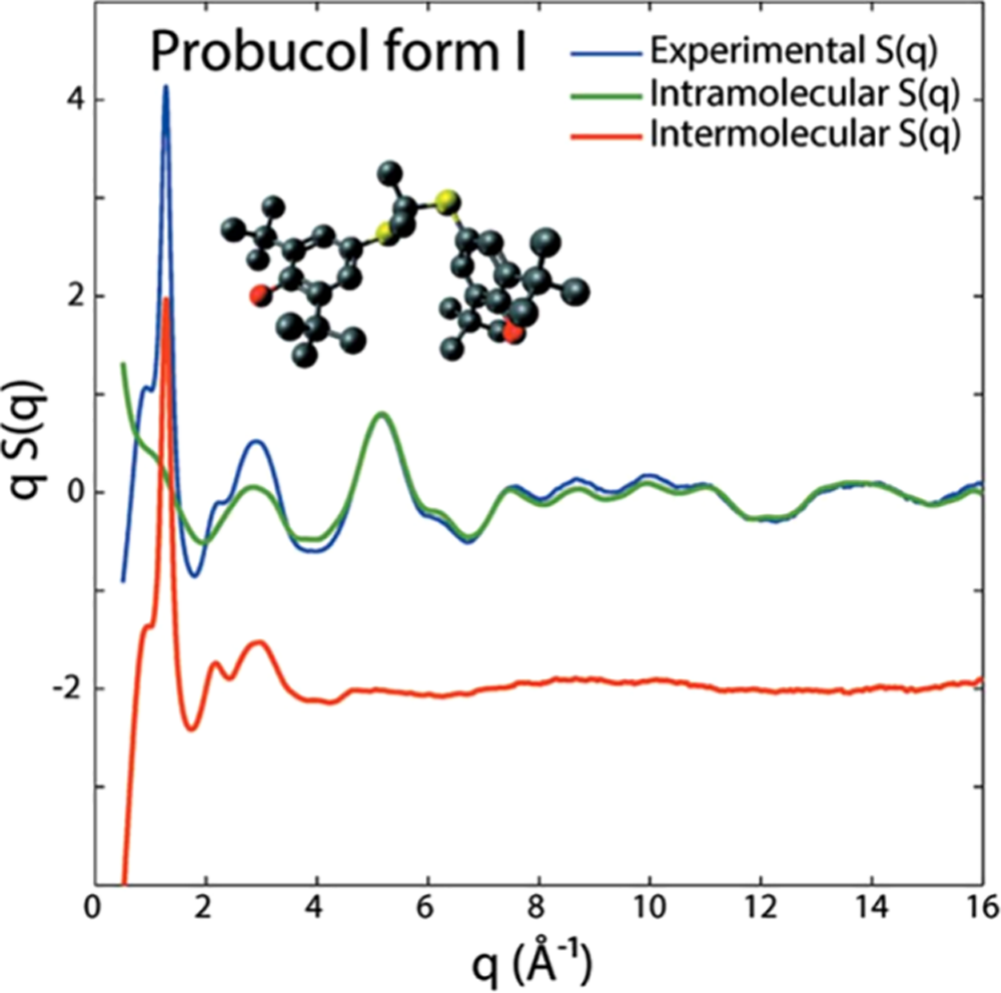
Modeling total scattering intensities in reciprocal space: fit of Probucol molecular structure to high-*Q* region. Here, *qS*(*Q*) is equivalent to the definition of *F*(*Q*) used in the current paper. Reproduced with permission from ref (464). Copyright 2015 International Union of Crystallography.

In materials where the diffuse scattering dominates, *F*_intra_(*Q*) can be simulated and compared to measured data, typically above 2–4 Å^–1^ depending on the extent of the molecular motif and its conformational flexibility. This is the case for noncrystalline molecular materials, and therefore *F*_intra_(*Q*) can be used to identify plausible molecular conformations absent a crystal structure. In crystalline molecular materials, increased thermal factors between molecules (and faster damping of low-*Z* form factors, e.g., in organic materials) means that the high-*Q* region is also mostly dominated by *F*_intra_(*Q*). Refinement can aid the analysis, using for instance
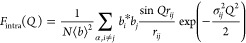
14as implemented in the program X-ray Intermolecular Structure Factor (XISF) from Mou, Benmore, and Yarger.^464^ The exponential term accounts for Debye–Waller effects, where σ_*ij*_ is the root-mean-square displacement for atom-pair *ij*. An example of fitting with XISF is shown in Figure 12.

**Figure 13:**
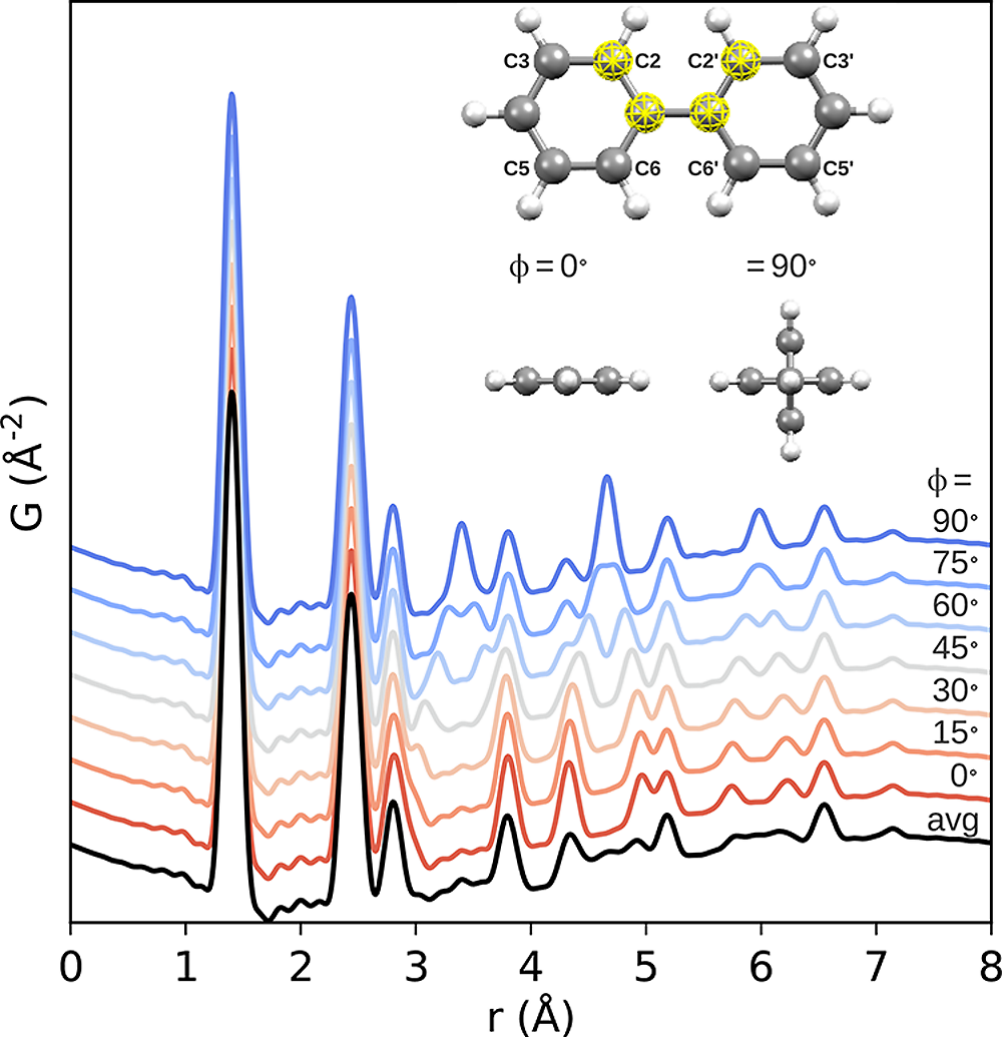
Conformational effects on the PDF: simulated PDFs offset from one another as a function of biphenyl dihedral angle from 0 to 90°. The black curve represents an average over all angles.

Similar analysis can be performed using the corresponding PDFs, with the additional benefit that unlike the *seemingly* arbitrary shape of the interference pattern, the real-space signal can be more intuitive to assess with respect to bond distances in the model. It is useful then to consider how changes in molecular conformation might affect the PDF signal. If free rotors exist in the molecular structure, then any rotation of these groups with respect to one another can change the pair-distance distribution. Consider as an example biphenyl (C_6_H_5_)_2_, which consists of two benzene molecules linked together by a single rotor, shown in Figure 13. For illustrative purposes, a planar conformation with torsion angle 0° is used as the starting point. As the torsion angle is rotated to 90°, the R- and para- carbons remain in plane with respect to each other. However, the ortho- and meta- carbons rotate out of plane, resulting in modified pair-lengths between the two separate phenyl groups. For example, as C2 rotates out of plane, the C2–C2′ distance increases, while the C2–C6′ distance decreases, until converging (C2–C2′ = C2–C6′) at 90°. One additional curve, shown in black, is the average of all prior rotation angles. This is what would be expected if the central bond is a free rotor. While biphenyl does not present a true free rotor,^465^ this averaging effect demonstrates how the observed signal will also be modified by a distribution of conformations present in the material. Of interest is the fact that some peaks in PDF are washed out, for example, the peaks between 4.5 and 5 Å and between 5.5 and 6.5 Å, whereas other peaks are preserved, for example the peak at 6.6 Å that comes from the carbon atoms that are widely separated in space but which lie on the axis of the rotor. Thus, PDF analysis can be be useful in the assessment of conformational details, particularly in disordered or noncrystalline materials that cannot be assessed using typical crystallographic methods and where distributions of conformations may exist.

In practice, the assessment of conformations in this way can be difficult due to significant overlap of intramolecular and intermolecular pair distances, for example, at ≳3 Å for organic molecular materials. For cases with significant intermolecular MRO or LRO, the analysis should consider both conformation and packing in space. However, single-molecule models can still be useful. If the correlation between atom-pairs in neighboring molecules is very weak when averaged over all possible neighbor orientations, for example in many amorphous materials, then the intermolecular signal will be very broad leaving the intramolecular signal to dominate the sharp signal over short distances. Thus, the assessment of conformations in a single-molecule model can serve at least two purposes. First, it can help to confirm which conformations or conformation distributions are consistent with the observed data. Second, it can help to identify how distributed the conformations must be to successfully reduce the simulated intramolecular coherence compared to the observed signal. If the orientations of neighboring molecules are on average highly correlated, for example, for the formation of dimers, then it may become necessary to use multimolecule models to simultaneously explore different dimer orientations.

**Figure 14:**
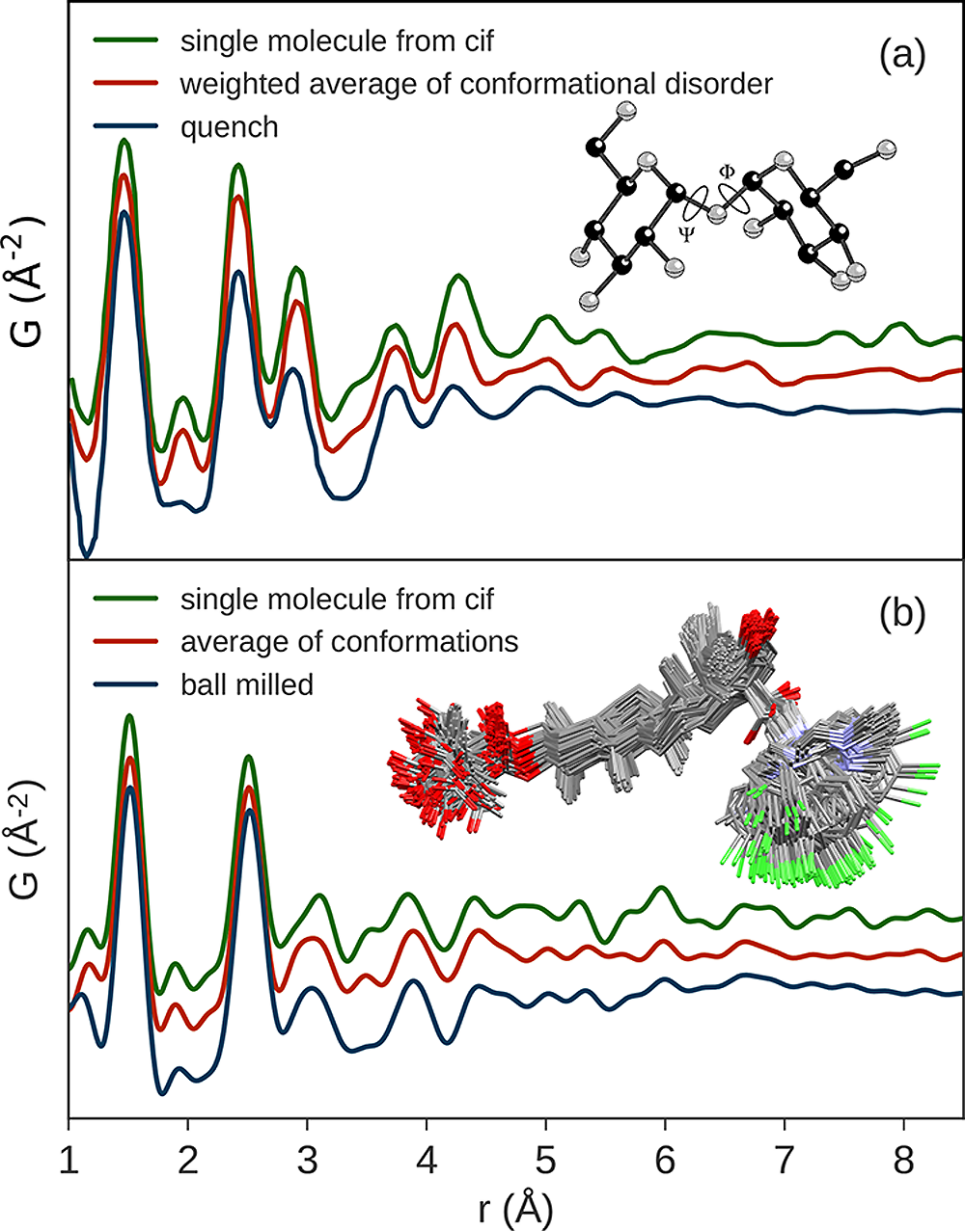
Experimental effects of conformational disorder in amorphous compounds: (a) comparison between PDFs of a single molecule from the crystal structure, a weighted average of conformers, and the measured data for melt quenched β-trehalose. The data were digitized from Bordet et al.^183^ with the conformer distribution determined from ^13^C SSNMR.^466^ A similar comparison is given in (b) for human immunodeficiency virus type 1 maturation inhibitor GSK2838232B; data are from Terban et al.^177^ In this case, the conformations are predicted from the program Mercury.^467^

Conformation distributions can be tested using spectroscopic or computational methods. Bordet et al.^183^ measured amorphous β-trehalose and found that the PDF simulated from a single conformer of the known crystal structure did not match well. A disordered model was built by varying the torsion angles of the two glycosidic linkages, and weighting the contributions according to the probability distribution reported from ^13^C SSNMR data.^466^ The qualitative improvement in the match between simulated and experimental PDF patterns is shown in Figure 14a. Another example is given for a human immunodeficiency virus type 1 (HIV-1) maturation inhibitor GSK2838232B in Figure 14b. In this case, an unweighted averaging of conformations predicted by using Mercury^467^ also gave substantially better agreement with the local structure observed for a ball-milled amorphous sample, as suggested by significantly broadened distribution of chemical shifts observed by ^13^C SSNMR.^177^ These observations show that for larger molecules with more conformational degrees of freedom, the sharp features in the intramolecular region can be dominated by the rigid subunits of the molecule, while correlations across the linkage of rotatable bonds are significantly broadened.

Similar concepts can be useful for the assessment of subunits within coordination compounds and other network-forming materials. Conformational distortions have been observed in a macrocyclic gadolinium chelate coordination polymer. On micronization, the crystal structure of Zn–Gd-DOTA-4AmP becomes amorphous, and disordering of the phosphonate terminated arms coordinating Zn^2+^ ions leads to a loss of coherence between the Gd and Zn.^468^ In amorphous zeolitic gel precursors, the presence of ring structures forming in the gels was detected prior to crystallization,^469^ as was the presence of seven-membered rings in nongraphitic carbons.^69^ PDFs can also substantially aid in identifying secondary building unit (SBU) structures. Mohideen et al.^470^ discerned the presence of Cu-tetramers versus Cu-dimers in a Cu-based layered coordination polymer, STAM-2, and Cliffe et al.^471^ determined the presence of Hf_6_ double clusters in 2D layers cleaved from hcp UiO-67(Hf) MOF along with the delamination mechanism. By comparing samples from a variety of synthetic procedures of different porphyrinic Zr-oxo MOFs, Koschnick et al.^324^ demonstrated that Zr_6_O_8_ SBUs in the MOF PCN-221 had been incorrectly assigned as Zr_8_O_8_ due to orientational disorder.

### 4.3 Bond Stiffness and Correlated Motion

The width of the peaks in a PDF represent the probability distributions for the interatomic distances associated with given atom-pairs. This distribution is affected by both atomic motion and static displacive disorder. When the disorder is sufficiently small, it can be described by a Gaussian distribution with some width. The widths are captured in isotropic, or anisotropic, atomic displacement parameters (ADPs),^472^ and highly anisotropic ADPs often indicate the presence of non-Gaussian disorder. In local structure studies, after noting the presence of significantly anisotropic ADPs in an average refinement, it is common to use different models for the disorder to achieve smaller and more isotropic ADPs.^427,429^ More generally, the ADPs obtained from crystallographic average refinements can overestimate the PDF peak widths for low-*r* peaks because of correlated atomic motions. When atoms are directly neighboring one another, or associated by distinct bonding arrangements, their dynamics tend to effect one another, for example, the thermal motions of neighboring atoms may tend to push and pull one another, thus decreasing the time-averaged distance distribution. This has the effect of narrowing the width of the associated peak.^473−475^

This presents a special challenge for molecular materials, because different types of interactions manifest between intramolecular versus intermolecular atom-pairs. This requires special treatments that are generally not needed for most inorganic materials. Covalently interacting, intramolecular atom-pairs will generally experience a higher degree of correlated motion, resulting in narrow peak profiles. Hydrogen and van der Waals bonding between separate molecular species is relatively soft, resulting in larger vibrational amplitudes and correspondingly much broader peaks in the PDF. The broad peaks in the high-*r* region become overlapped and show up as broad, complex features that are not well separated, but are representative of the underlying structure, such as functional groups or entire molecules. These peaks are a direct result of conformation and packing of the molecules, but also reflect the inherent disorder in molecular packing in many molecular systems. Similar behavior also occurs for correlations between flexible ligand molecules and inorganic clusters in MOFs, as well as molecular segments associated by distinct network connectivity, as seen for example in some amorphous MOFs.^476^

**Figure 15:**
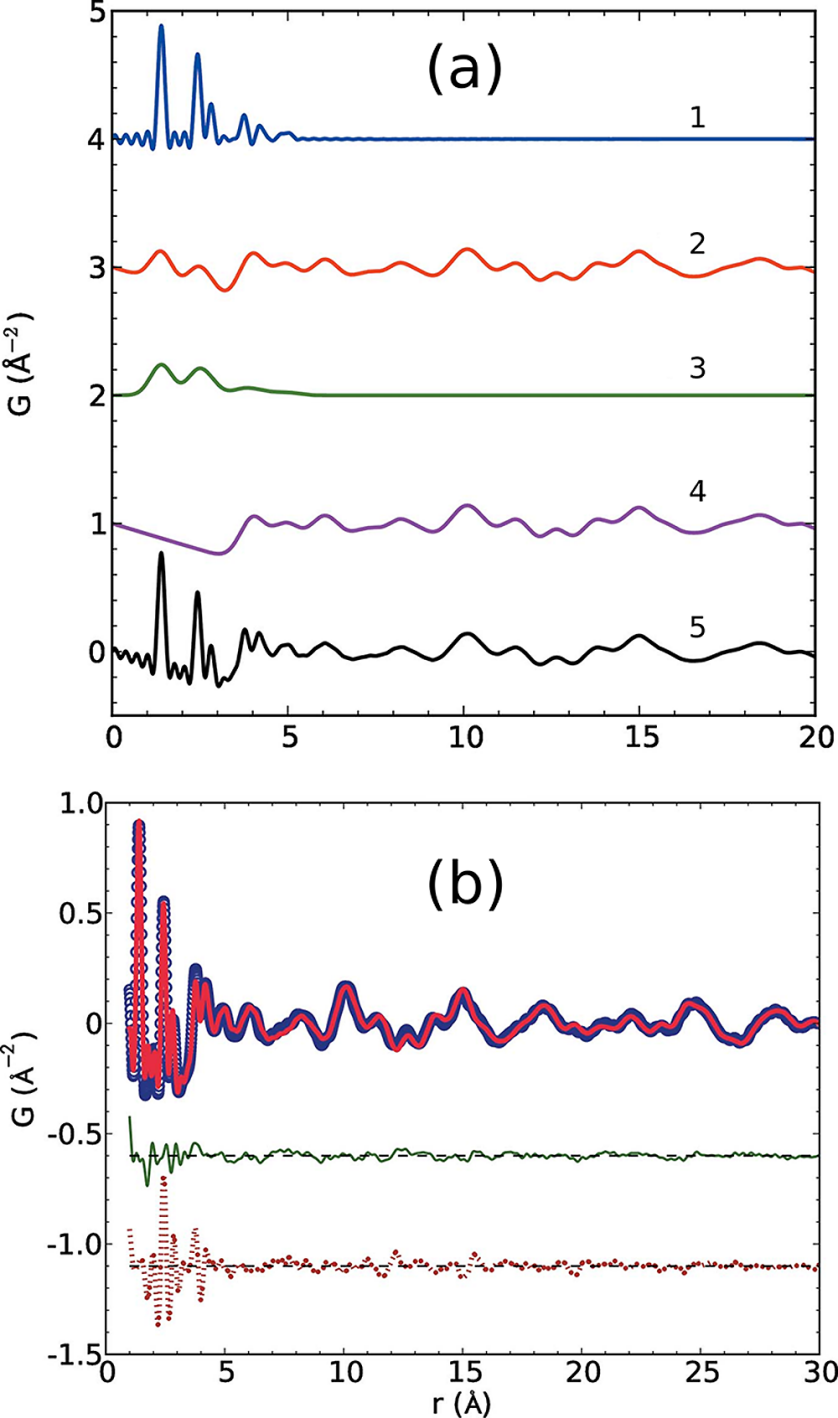
Systematic illustration of intra- and inter-molecular bond contributions to the PDF of a small molecule solid: (a) simulated PDF patterns of (1) *G*_*m*2_(*r*), (2) *G*_*c*_(*r*), (3) *G*_*m*1_(*r*), (4) *G*_*c*_(*r*) – *G*_*m*1_(*r*), and (5) *G*_total_(*r*), as in eq 15. (b) Resulting refinement. The blue curve is the experimental PDF of naphthalene. The red curve represents the corresponding calculated PDF using the new approach with two isotropic displacement parameters. The green curve depicts the fit difference, while the red line at the bottom shows the fit difference from the standard approach. Adapted with permission from ref (175). Copyright 2015 International Union of Crystallography.

The program PDFgui^475^ suggests the parameters δ_1_ or δ_2_ for describing correlated motion, with linear and quadratic *r*-dependence of the peak broadening, respectively. This behavior has a basis in theory (the correlated Debye model),^473,474,477^ but is more often just used as convenient fitting parameters. Where there is a sharp cutoff between highly correlated low-*r* peaks and less correlated intermediate-*r* peaks, it is possible to use the **sratio** parameter. This is a reasonable approximation for the step-function-like sharpening behavior of rigid molecules up to a cutoff distance of **rcut** beyond which intermolecular interactions begin to dominate.^475^ However, this is not ideal, because there is a range of distances over which intramolecular atom-pairs and intermolecular pairs coexist, meaning that sharp peaks are present sitting on top of broad peaks. In this case, a distinct modeling procedure has been described in which separate ADPs are used to describe the mean squared displacements (e.g., *U*_*ij*_, *B*_*ij*_) of intramolecular and intermolecular pairs, respectively, in the simulated PDF. This is implemented for example by Rademacher et al.^478^ using the program DISCUS,^479^ and by Prill et al.^175^ using the program Diffpy-CMI.^480^ This can be performed using Diffpy-CMI by first calculating the PDF from a crystal *G*_c_(*r*) as described above, with ADPs *U*_inter_. Then, the PDF is separately calculated for an individual molecule *G*_*m*1_(*r*) with *U*_inter_ and subtracted. Finally, the individual molecule PDF is calculated again *G*_*m*2_(*r*) but with separate ADPs *U*_intra_ and added, giving the total contribution
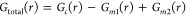
15The procedure is visualized in Figure 15 as implemented by Prill et al.^175^ in the case of naphthalene. Similar principles were discussed earlier by Narten and co-workers for applications in molecular liquids.^481^ For flexible, macromolecular fluids, for instance, it is no longer possible to uniquely separate the total scattering structure function (or PDF) into intra- and inter-molecular contributions. However, the long-range intramolecular interactions can be assumed to be screened out by intermolecular interactions, and therefore, the intramolecular component can be approximated at the monomeric length-scale.^482^ Separate intra- and inter-molecular atomic ADPs have also been utilized for modeling chain structure in the solid state.^130,137^

In TOPAS v6 and up,^483^ this discrepancy in intra- versus inter-molecular peak widths can also be modeled by designating specific atomic-pairs within the unit cell using the **pdf_for_pairs** macro, which allows a new peak broadening parameter or function to be defined that overrides the previous thermal parameter descriptions. For example, in a unit cell where all atoms are generically described with thermal parameter **beq = Biso**, if C1 and C2 are two sites sharing a chemical bond, then the broadening of the corresponding peak can be redefined by defining a new parameter for the intramolecular peak width:**prm Bintra 0.1**then specifying the corresponding bond with**pdf_for_pairs****”C1”****”C2”****pdf_only_eq_0****pdf_gauss_fwhm = Bintra;**

In this way, the distinct intramolecular pairs may also be described as an *r*-dependent function, and sections of larger molecules may be segregated into distinctly correlated subunits to account for conformational distortions.^177^ More information can be found in the TOPAS technical reference^484^ and the PDF macros on the TOPAS wiki page.^485^

### 4.4 Molecular Coordination and Orientation

**Figure 16:**
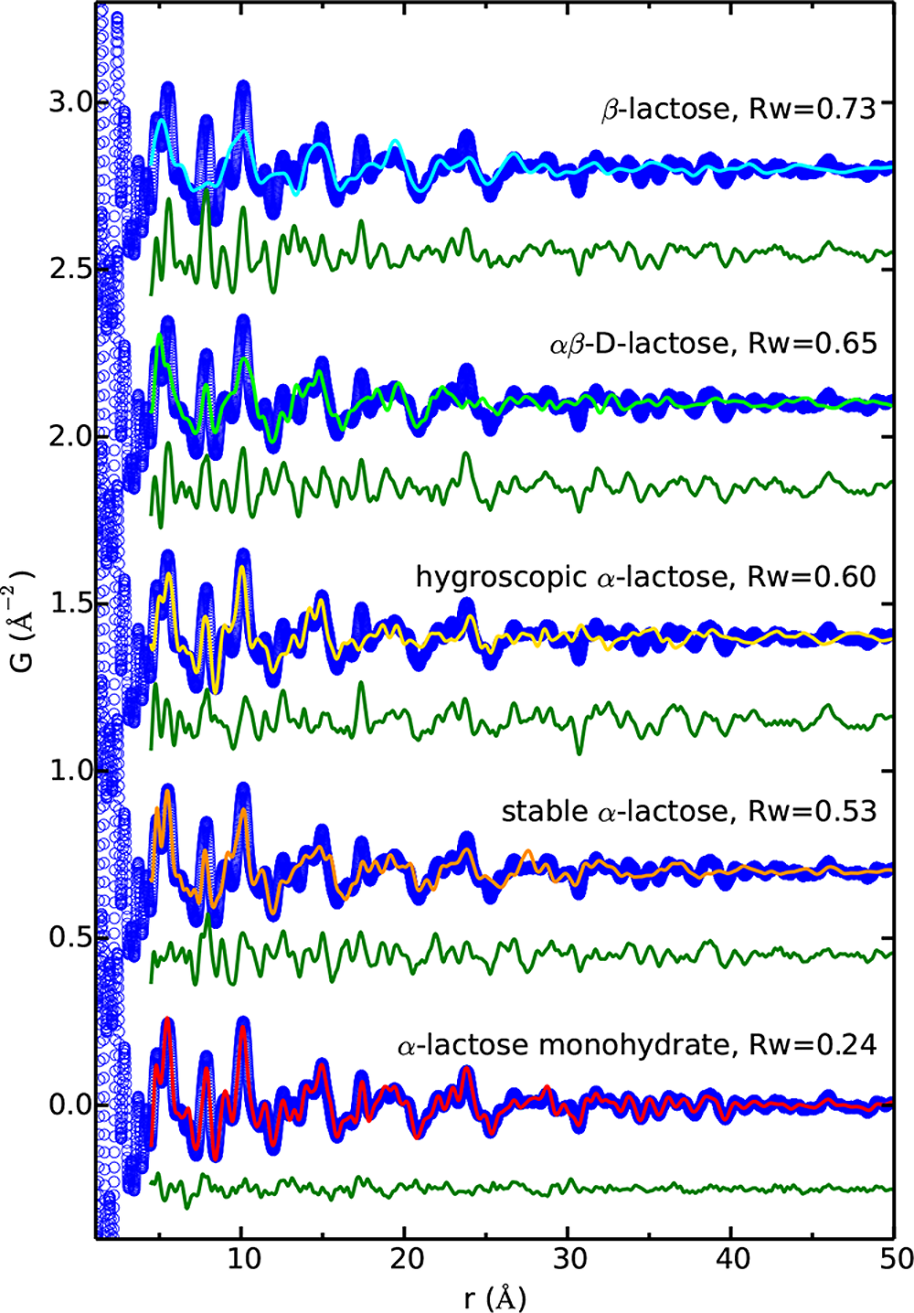
Refinement of different polymorphic structure models (anhydrates and hydrates) to the experimental PDF for lactose: Similarities in local intermolecular packing between models allow for varying degrees of agreement to some features, even for models with the wrong packing, but the models can be differentiated from fine-scale features that are sensitive to the packing. Figure reproduced with permission from ref (38). Copyright 2015 American Chemical Society.

By focusing on the intermediate and long-distance regions of the PDF, information on the intermolecular packing arrangement can be determined. The signal depends on both the conformations and 3D locations and orientations of the molecules. For highly crystalline materials, this provides comparable information to reciprocal-space analyses of the Bragg peaks. For less well ordered materials, it provides further insights. Because the atomic density distribution seen in the PDF is a complicated function of 3D information collapsed onto one axis, it can sometimes appear confusing for distinguishing molecular arrangements. However, these real-space functions are highly sensitive to even small molecular rearrangements. This is shown by comparing different polymorphic and/or hydrated forms of lactose to a measurement for α-lactose monohydrate in Figure 16.^38^ Similarities exist between simulated patterns from different structural models reflecting similarities in the preferred intermolecular neighbor packings between the (energetically) viable models. Differences between the distinct packing models can be differentiated from fine-scale features that are sensitive to these differences. It is useful to point out how the PDF reflects molecular packing information in a more intuitive way than the Bragg scattering in reciprocal space. Structures with similar intermolecular packing arrangements but which, nonetheless, result in LRO with different symmetries and unit cells will yield diffraction patterns that, to the eye, appear to be quite unrelated, but PDFs that are highly similar. In this way, PDFs can give a better measure of similarity in packing between models, to help the scientist compare and contrast intermolecular associations in different polymorphic forms.

This information about molecular packing persists in the PDF even when the material is not well crystallized, for example, in nanocrystalline drug and food compounds,^38,172^ poorly crystalline pigments,^486^ or thin-film materials.^487^ This is also evident for materials that undergo conformational or orientational order–disorder transitions or form plastic phases. In such cases, insights can be gained by comparison with related crystalline forms such as for *p*-terphenyl^478^ and cyclohexane.^488^ Head-to-tail disorder of molecules was studied for the case of 2-monomethyl-quinacridone (Pigment Red 192).^489^ A preferred local orientational ordering of molecules was observed along one diagonal direction, in agreement with theoretical calculations.

**Figure 17:**
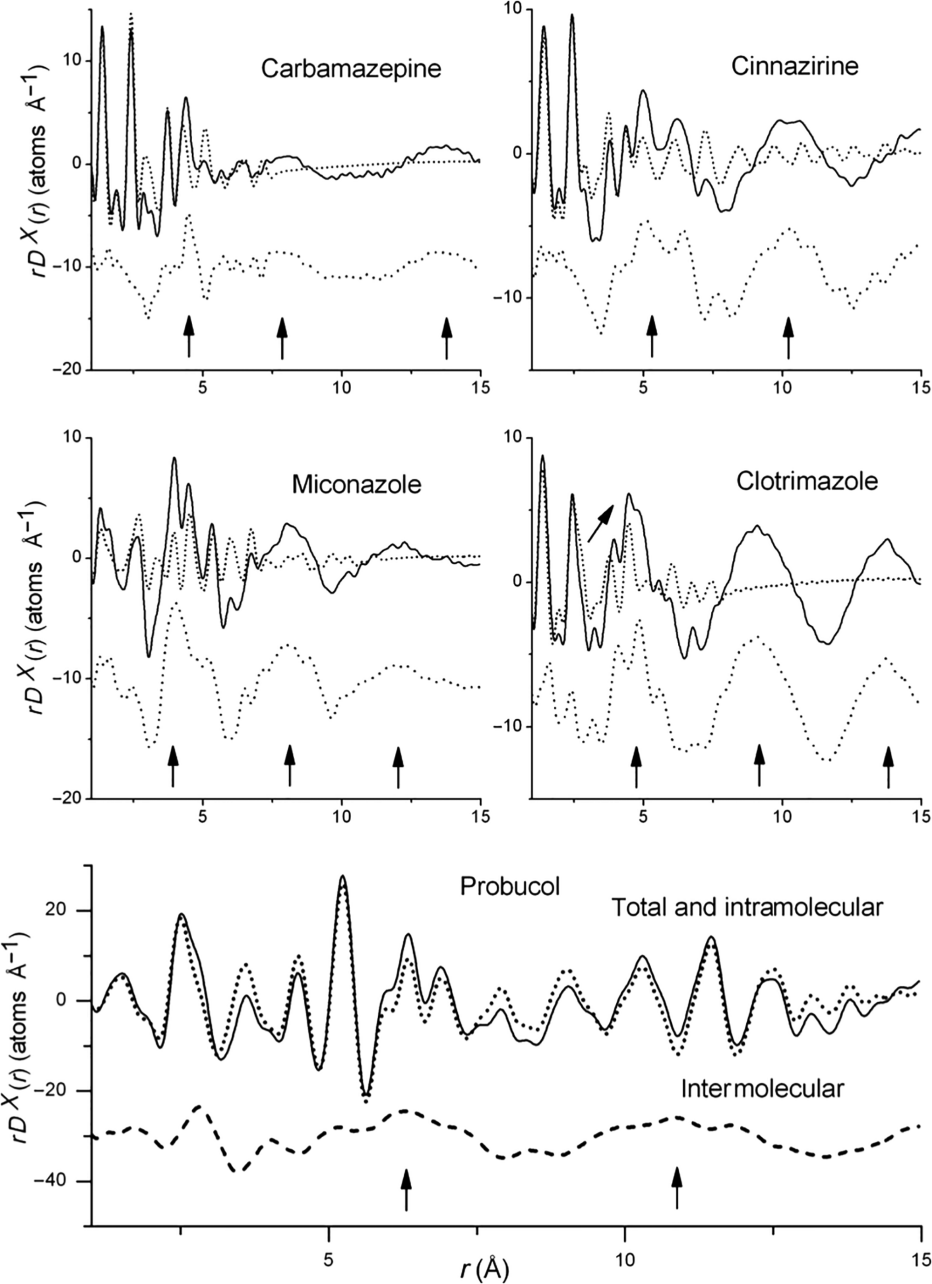
Extraction of intermolecular features from disordered pharmaceutical PDFs: measured PDFs (solid lines) are multiplied by *r* to highlight the medium-range interactions and compared with the intramolecular curves calculated from the crystal structures (dotted lines). The difference between the solid and dotted lines corresponds to the intermolecular PDF (dashed line below) with maxima highlighted by arrows. Here *D*(*r*) is a slightly different normalization of *G*(*r*).^31^ Reproduced with permission from ref (173). Copyright 2013 Elsevier.

For amorphous structures with no LRO at all, there often still exists some MRO defined by the preferred intermolecular arrangements, with correlations typically existing over a few molecular packing distances,^38,173,177,204,205,424^ as shown for melt-quenched active pharmaceutical ingredients: carbamazepine, cinnazirine, miconazole, clotrimazole, and probucol in Figure 17. For small rigid molecules, the frequency in *r* of broad correlations in the PDF can give some insight into the preferred packing orientations of molecules. However, for larger, more flexible molecules, they can rather reflect the distance of closest approach between different subunits averaged over different conformations.^204,205^ The peaks of the extended, intermolecular correlations in PDFs of amorphous molecular materials can be around an order of magnitude smaller than those in the ordered form, and close to 2 orders of magnitude smaller than the intramolecular peaks, therefore requiring amplification for example by *r* × *G*(*r*),^173^ and high quality data with minimal noise in the high-*Q* range. Lower *Q*_max_ cutoffs may be used in combination with modification functions to reduce termination effects and increase the visibility of the structural correlations at higher distances (see section 7.4).

The study of molecular spatial and orientational correlations in liquids has significantly benefitted from the use of MD and EPSR simulations (sections 3.3.3 and 6.3) to develop the most probable spatial density function (SDF) to describe the intermolecular density correlations or *solvation structure*.^238,490−493^ In a study of acetone and dimethyl sulfoxide, McLain et al.^296^ determined that both liquids show a high degree of preferred molecular orientations within the first coordination shell, dictated by the underlying symmetry of the respective molecules. Both liquids showed a preference for antiparallel alignments of dipole moments, but with a broader distribution for acetone and a more specific *head-to-tail* arrangement of the S=O···S=O bonds.^296^ Headen et al.^292^ studied the nature of aromatic π–π interactions in liquid benzene and liquid toluene. Benzene molecules were found to be the more highly correlated, but both benzene and toluene were found to contain coordination shells with an average of approximately 12 neighbors. At small separations (<5 Å) parallel displaced orientations were favored where the neighbor is slightly shifted to mimic that observed in the interlayer structure of graphite (and similar to stacking offsets in COFs^374−376,379^). At larger separations (>5 Å), interactions are dominated by an *edge-to-face* interaction where two H atoms are directed toward the acceptor aromatic ring (similar to the motif observed in crystalline benzene^494,495^ or as suggested in the packing of neighboring polyurethane chains^137^).

### 4.5 Chain Packing

**Figure 18:**
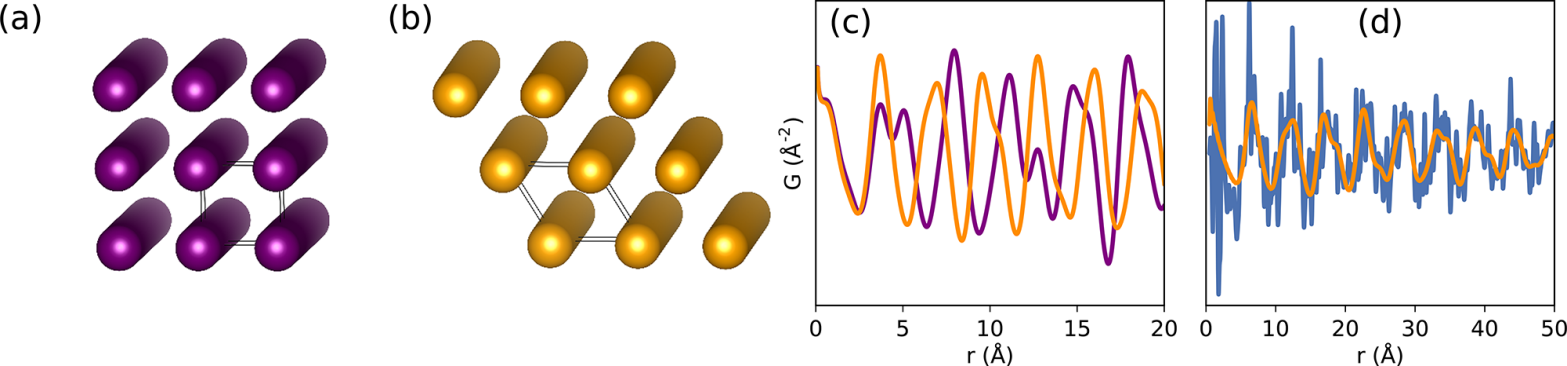
Demonstration of interchain packing: (a) square (purple) and (b) hexagonal (orange) stacking of cylindrical columns and (c) the associated density distribution functions. (d) The hexagonal stacking model (orange) was fit to the PDF simulated from the structure of δ poly(lactic acid) (PLA) (blue).

Chain- or 1D-like motifs are a common structural feature of many organic polymers and coordination compounds. Differences in the bonding properties along the chain versus between neighboring chain structures can affect the relative sharpness or broadness of the associated peaks, similar to the broadening observed for intermolecular peaks from small molecules. Furthermore, the effects of chain rigidity or persistence length and the overall small contribution from the pairs along a single chain to the total PDF at higher distances can lead to a significant loss of distinct atom-pair peak details at longer distances along the chain.^481,482^ Thus, the MRO and LRO signals in the PDF, if present, often reflect lower frequency density modulations that result from the packing directions perpendicular to the 1D chains. This is demonstrated simply using the density fluctuations of stacked cylinders. Square and hexagonal stackings of cylinders lead to different density distributions. The change in unit cell angle from 90° to 120° leads to a contraction of the 2nd neighbor distance in the square stacking model, which becomes equal to the first neighbor distance, as shown in Figure 18. Compared to the atomic PDF calculated for δ poly(lactic acid) (PLA) that has an orthorhombic unit cell but hexagonal-like packing of spiral chains, the hexagonal cylinder model reproduces the low frequency modulations in the density giving an accurate value for the nearest neighbor interchain distance.

As discussed in section 3.2.1, many studies of amorphous and molten polymer structures have attempted to determine local chain packing motifs by the relative positions and amplitudes of broad maxima extended over SRO and MRO distances. However, a difficulty in this endeavor arises in that the first sharp diffraction peak of the amorphous polymer scattering patterns predominantly arises from a fairly well-defined distance of closest approach between molecular subunits,^95,112^ rather than a particular packing pattern of necessarily rigid chain segments. Voigt-Martin and Mijlhoff also discussed a concern that a potentially significant source of error in such analyses could result from the generation of low frequency termination oscillations due to the *Q*_min_ cutoff (see section 7.4 for further discussion).^496^ It is difficult to make this assessment *a posteriori* for claims made in early papers. The observation of long-wavelength ripples in the PDF certainly implies the presence of some kind of density modulations present in the structure, but care must be taken not to read too much into the value of the wavelength unless it has been established to not be affected by variations in *Q*_min_ (see section 5.8). In situations such as these, conventional RAPDF data can be augmented with data collected on a detector at a further distance from the sample (i.e., medium angle X-ray scattering or MAXS) or even a full-on small angle scattering measurement.

**Figure 19:**
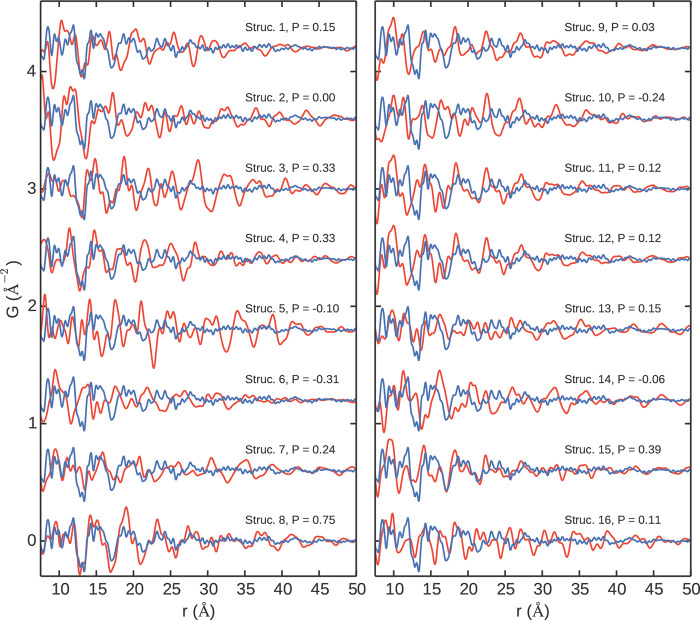
Comparison of candidates models with different conformations and interchain packing to the experimental data: PDFs simulated from different models of MDI:BD thermoplastic polyurethane models are compared to the experimental data. Ranking by Pearson correlation coefficient (see section 5.1) allowed for identification of the best candidate, Struc. 8. Reproduced with permission from ref (137). Copyright 2016 American Chemical Society.

Many poorly ordered, semicrystalline polymers do have some degree of LRO present, and the resulting long distance signals can be used to differentiate different polymorphs^137,139^ and to differentiate the packing characteristics resulting from different processing methods.^32^ In thermoplastic polyurethanes synthesized using 4,4′-methylene diphenyl diisocyanate (MDI) and 1,4-butanediol (BD), kinking of the diphenyl-methane leads to oscillatory behavior along the chain axis and more complex intermolecular packing motifs, and the chain packing can no longer be thought of in terms of linear packing motifs. Different models were constructed by using either gauche or trans configurations of the central bond in the BD fragment, different orientations of the urethane linkage with respect to the neighboring diol and phenyl segments, and varying the dihedral angle of the diphenyl–methane segment.^137^ This resulted in different chain packing motifs in the direction perpendicular to the hydrogen bonding direction. A particular match to the intermolecular packing pattern could be discerned through a combination of similarity comparisons as shown in Figure 19 and subsequent structure refinement.

### 4.6 Layer Stacking

Layered motifs are a common feature in many materials.^497,498^ In general, differences in preferred bonding interactions within and between layers lead to a variety of interesting disordering mechanisms that can complicate the idea of a crystal and therefore the assessment or solution of its structure.^499^ These can include relative shifts or rotations between layers, interstratification by other chemical species, or delamination effects leading to true 2D materials. When possible, candidate stacking vectors should be derived using high-resolution reciprocal-space diffraction data or by comparison to ordered variants, although PDF data can be very useful for constraining intralayer bond distances and verifying local stacking motifs, such as in honeycomb structures.^500,501^ In other cases, diffraction analysis may not be feasible due to severe stacking disorder or even nanosized in-plane dimensions, and this is where PDF methods can become particularly useful.

**Figure 20:**
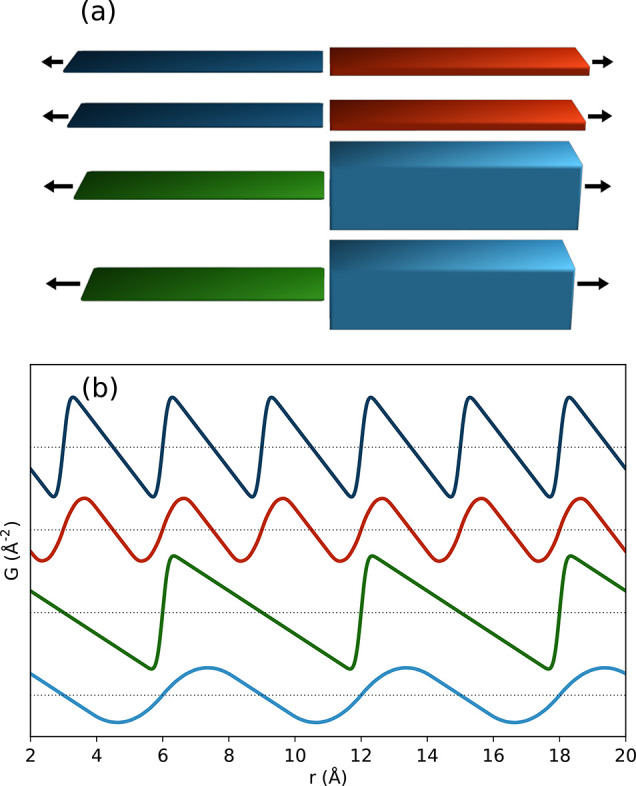
Representations of interlayer packing for turbostratic layers: (a) periodically stacked planes of constant electron density and (b) the associated PDFs (offset for clarity with dotted lines representing *G*(*r*) = 0). Dark blue and red represent thin and thicker layers with a spacing of 3 Å, and green and light blue represent thin and very thick layers with a spacing of 6 Å.

A feature of many disordered layered materials is turbostratism, or the random orientational arrangement from one layer to the next.^43^ In these cases, the interatomic correlations coming from atom-pairs located in separate layers is smeared out such that only information on the individual layer structure, along with the average density distribution in the direction of the layer stacking, can be obtained. As a simple demonstration, in Figure 20, consider the PDF for parallel planes of uniform electron density, with no distinct atomic sites within the layers. For very thin planes, the density distribution appears as a sawtooth pattern where the PDF sharply rises at each distance-of-closest-approach between planes and then falls off more gradually to higher distances. This, and for the same reasons, resembles the well-known Warren line-shape in diffraction.^43^ Increasing the thickness of the layers broadens these sharp features into an increasingly sine-wave-like oscillation. For an isolated single layer, with no stacking, these oscillations would not occur, and the PDF can then be described by a single layer model, for example in the case of delaminated layered double hydroxides (LDH).^461^ In cases of severe turbostratic disorder, these oscillations may be negligible if the ordering between layers is also very weak, for example, if the layers are not parallel or are bent. For the most information, the oscillatory behavior can be extracted from the fine-scale features due to the atomic-structure and analyzed separately from the intralayer components.^371^ Another possibility is to use a hybrid modeling approach with the intralayer structure described by an atomic model and the interlayer density fluctuations described by a second model with smeared electron density, as above, or an analytical description, for example, in the style used to model the radial distribution functions of some glassy carbons.^51,55^ Turbostratic disorder can also be approximated using anisotropic ADPs. Petkov et al.^60^ modeled the out-of-plane disorder in nongraphitic carbons by allowing for larger ADPs in the out-of-plane direction to dampen the interlayer correlations.

**Figure 21:**
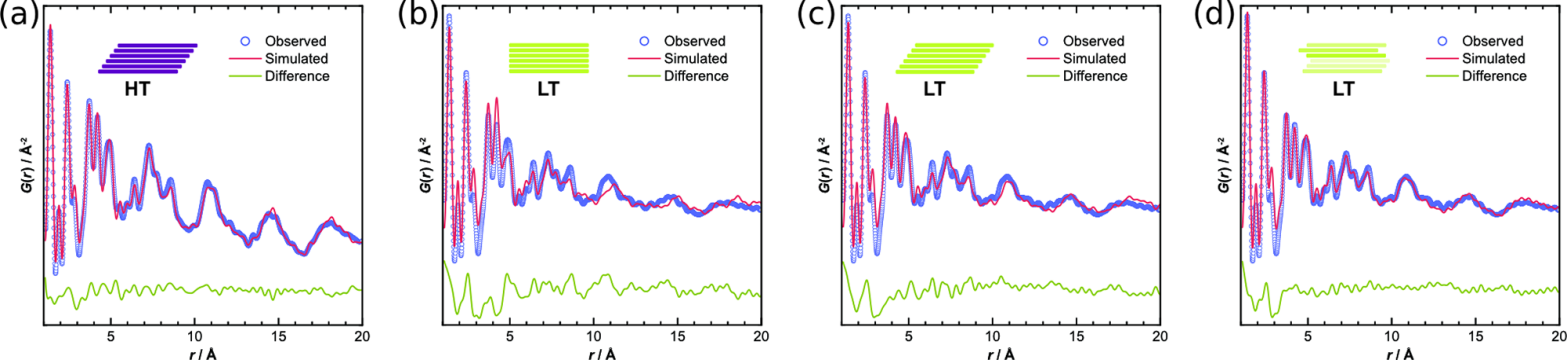
Different COF layer stacking models fit to experimental data: best PDF fits obtained from refinement over 1–20 Å to (a) the ordered HT-TTI-COF assuming unidirectionally slipped stacking (*R*_w_ = 12%) and of LT-TTI-COF, assuming (b) eclipsed (*R*_w_ = 32%), (c) unidirectionally slipped (*R*_w_ = 26%), or (d) randomly slipped stacking (*R*_w_ = 21%). Reproduced with permission from ref (376) under CC BY 3.0 license.

In many cases, fairly definite types of interlayer associations are present. For example, slipped stacking of neighboring layers in preferred directions is observed in porous 2D polymers including imide and amide COFs^376,379^ and poly(heptazine imide) (PHI).^377^ Although the long-range ordering of the slipping direction can be lost, the signal of local interlayer interactions is still preserved in the PDF if specific relationships are preferred between neighboring layers. For example, refinements of ordered slip-stacked, eclipsed, and randomly slipped models against the PDFs of ordered (HT) and disordered (RT) TTI-COF demonstrate the level of misfit, and improvement expected, for these different descriptions of the local layer stacking, Figure 21. In this case, even though an eclipsed model fits the diffraction pattern very well in reciprocal space, it is clear that the local structure prefers offsets of neighboring layers by the better agreement with the unidrectionally slipped model of the HT phase. Refinement instead of models with random slipping directions showed substantially better agreement in real space, and could fit the reciprocal-space diffraction pattern without the use of large valued microstrain parameters. As related to the use of separate in-plane versus out-of-plane thermal parameters for such layered motifs,^32,60,376^ the reduced correlations between layers can result from both disordered stacking relationships as well as conformational flexibility.^379^ When using anisotropic out-of-plane ADPs, it is important to keep track of correlations between ADPs, layer conformation, and the stacking vectors used to describe the modifications to the layer positions.

**Figure 22:**
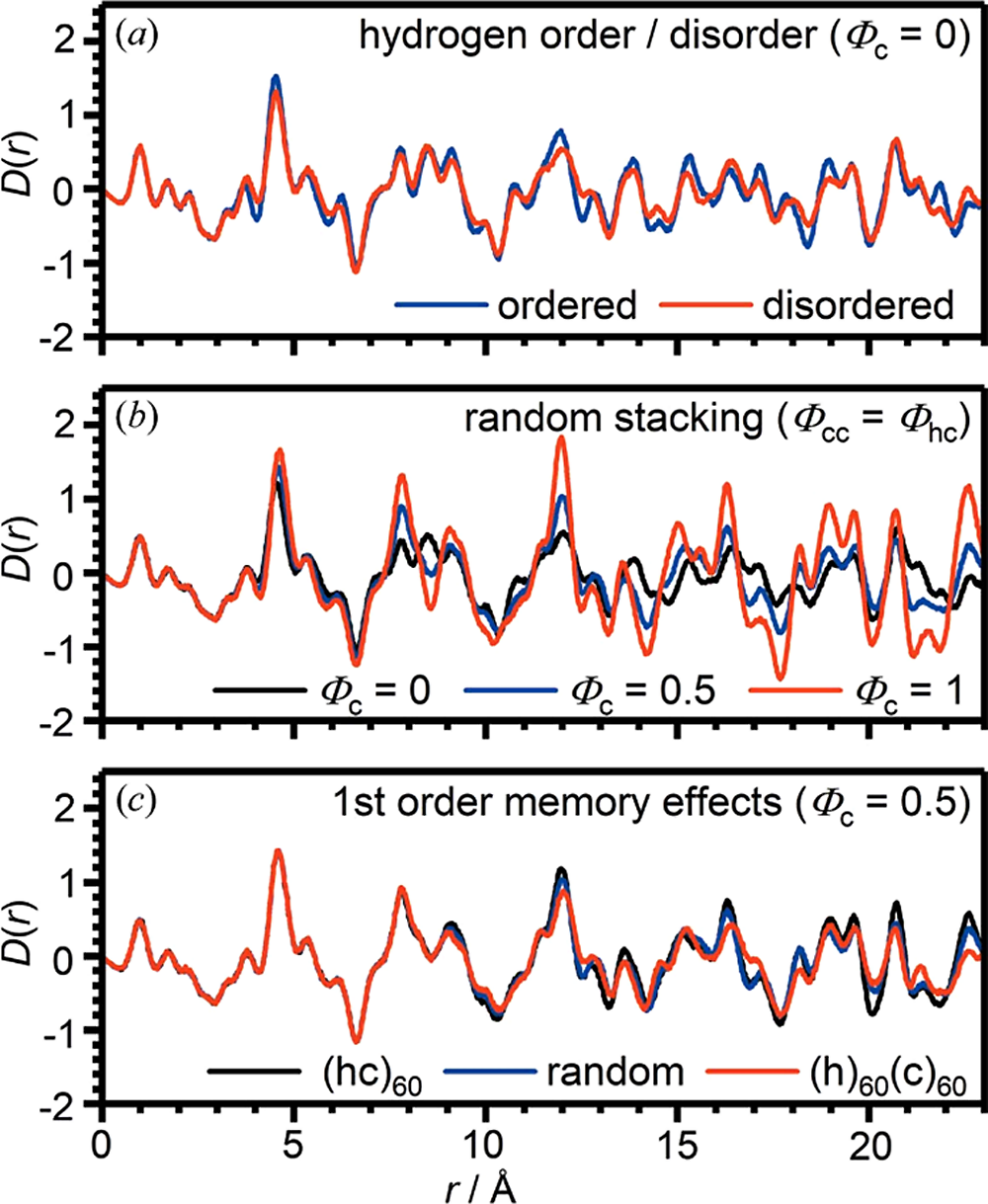
Influence of different interlayer structural characteristics on the calculated PDFs: (a) hydrogen-ordered/disordered ice Ih, (b) hydrogen-disordered ice I with cubicities of 0, 0.5, and 1, and (c) hydrogen-disordered ice I with 0.5 cubicity and different memory effects (strictly alternating, random stacking, and two slabs of cubic and hexagonal stacking. Here *D*(*r*) is a slightly different normalization of *G*(*r*).^31^ Reproduced with permission from ref (509). Copyright 2018 International Union of Crystallography.

Refinements to the local structure may be used to obtain a crystallographic unit-cell-type description of the local structure in layered materials where in which a solution from reciprocal space is not possible.^385,390,392,502^ Information about the presence and density of particular types of local interlayer interactions may also be accessible in nanomaterials for which features in reciprocal space are too diffuse for approaches focused on Bragg peak analyses.^503,504^ In practice, models of different stacking sequences can be constructed and then fit as separate phases to the experimental PDF up to the repeat distance of the stacking sequences in the model. Allowing random displacements in many-layer, supercell models can be useful for verifying the correct intralayer structure and likelihood for stacking faults,^505^ but this style of refinement is typically not very useful for determining precise kinds of faults that exist within the material or their probability. Most work in this area has focused on the simulation of the diffraction effects due to different kinds of stacking faults in reciprocal space, for instance the recursive approach used by DIFFaX.^506,507^ Recent efforts using statistical analysis of Markov chain Monte Carlo sampling has been suggested as a means to estimating the most probable stacking configurations from real-space data.^508^ Other recent work by Playford et al.^509^ has used experimental PDFs to determine the fractions of cubic versus hexagonal stacking between layers of water molecules in ice crystals. They discussed that the PDF data were less sensitive than the diffraction data to higher order memory effects, or “Reichweite”, where the probabilities of 2nd, 3rd, and so on, stacking events are affected by the first neighbor association. Because of this, they suggested that the PDFs may actually be a better measure of the percentage of cubic stacking events (cubicity). Figure 22 shows the effects of hydrogen disorder, cubicity, and different types of memory effects on the PDFs. Overall, it is expected that with high quality data and careful analysis, further development in the extraction of information from real-space refinements may be possible.

### 4.7 Micropores

**Figure 23:**
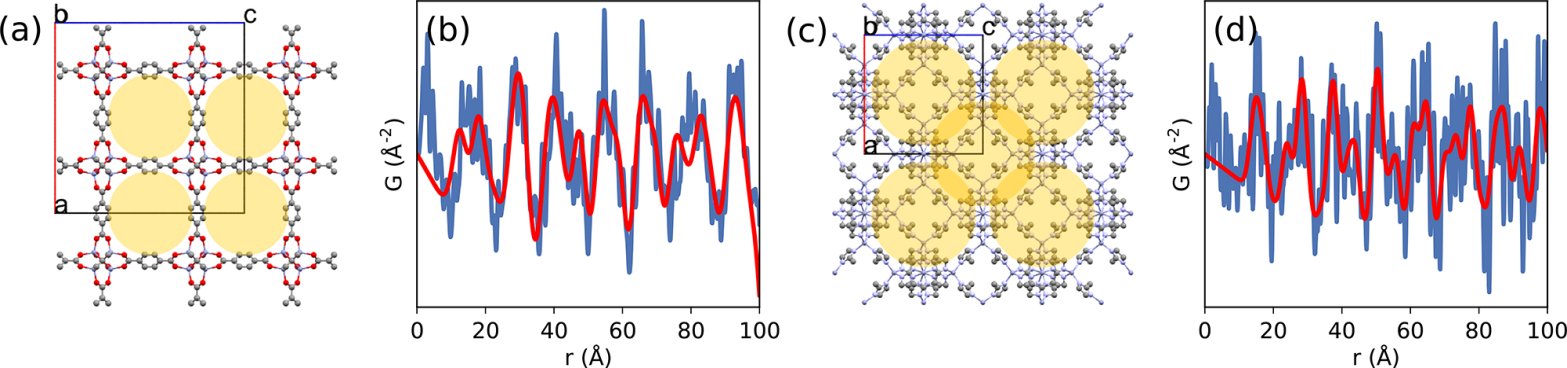
Demonstration of packing of pores in a periodic structure and the resulting density modulations present in the respective PDF signal: the long-wavelength signal can be thought of as the autocorrelation of the pores (red), superimposed onto the PDF of the discrete atom–atom pair distances (blue). Examples are shown for frameworks with both (a,b) simple cubic packing for MOF-5 and (c,d) body centered cubic packing for ZIF-8.

The pores in ordered framework-type materials can be considered 3D tesselations in space that, like the atoms in a crystal, also have a distinct phase relationship. Since the PDF is the autocorrelation of the electron density, it therefore intrinsically includes the correlations of the regions lacking electron density. To understand how the real-space signal of the pore structure manifests, one can consider the PDF for the inverse structure where pores are represented as a pseudoatomic solid; wherein the atom positions represent the pore distribution. This is demonstrated in Figure 23. Here, the PDFs have been simulated for two metal–organic frameworks, MOF-5 for which the pores are packed in a simple cubic (SC) arrangement and ZIF-8 for which the pores are arranged in a body-centered cubic (BCC) manner. To illustrate the simple, pseudoatom approximation, the corresponding monatomic SC and BCC models are utilized, with lattice dimensions rescaled accordingly. The respective PDFs are superimposed showing that the diffuse, long-wavelength density fluctuations are described well by this model. Similar approaches have been used to model the pore structuring in layered porous structures.^371,376^ In many cases, such voids within framework structures may be filled with gas or solvent molecules, and the composition, amount, and relative structuring should also be considered. Pore filling may act to suppress the amplitude of these low frequency oscillations due to the decrease in the density difference between the framework and occupied interstitial space.^369,377^

**Figure 24:**
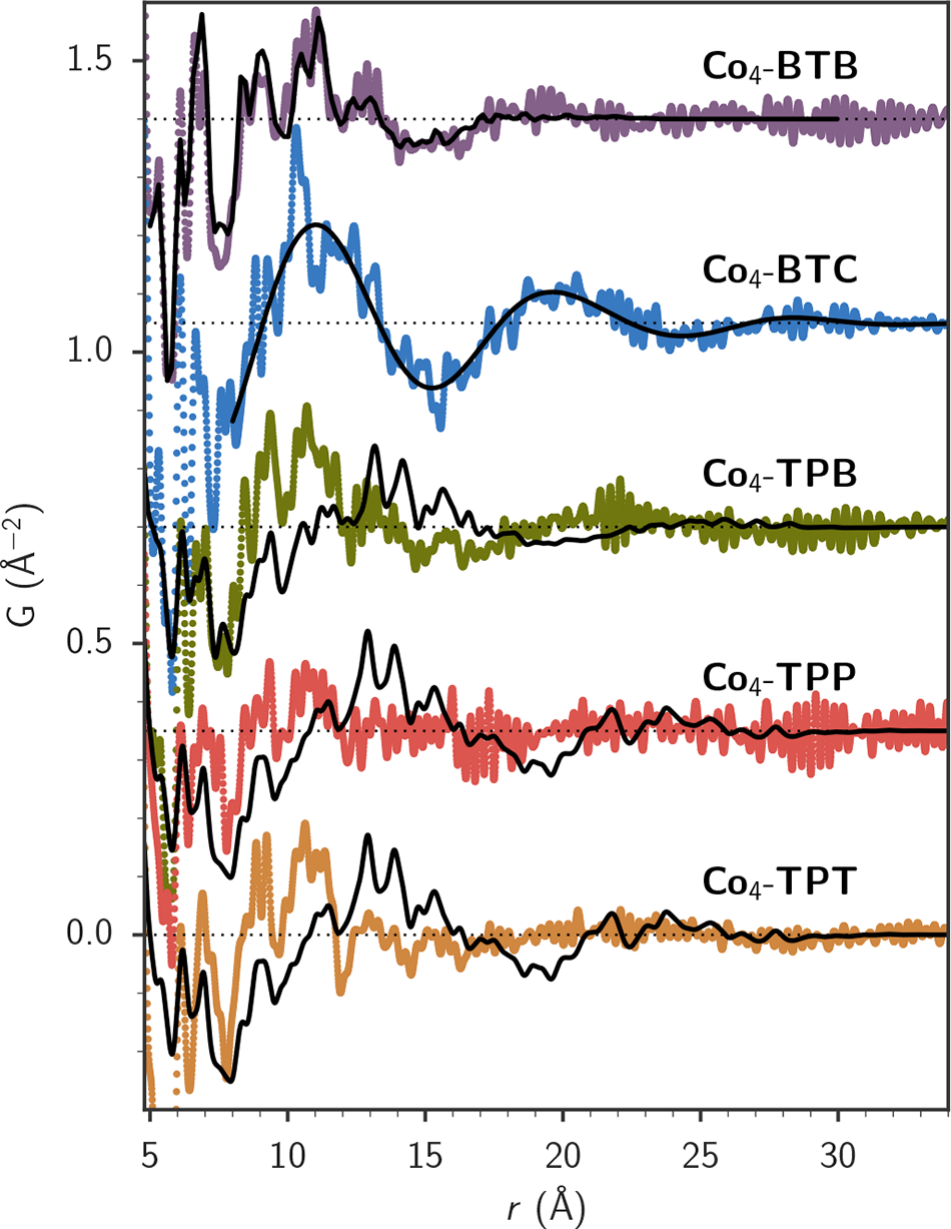
Short-range ordering of pore structure within disordered, metal–organic network stabilized Co_4_O_4_ frameworks: experimental data (colored curves) are fit with DFT-derived crystal structure models (black). Reproduced with permission from ref (341). Copyright 2019 National Academy of Sciences of the United States of America.

The observation of low frequency oscillations in the experimental PDFs can be useful for probing the presence and possible geometry of void structures present in a compound. This idea has been used to index the local pore structuring of microporous, amorphous MOFs against potential crystallographic models determined using model building and DFT relaxations.^341^ The MOFs incorporated cobalt-oxo cubane (Co_4_O_4_) clusters to be used as a catalyst for the oxygen evolution reaction, and the clusters were connected with different organic linkers including 1,3,5-tris(4-carboxylatophenyl)benzene (Co_4_-BTB), 1,3,5-benzenetricarboxylate (Co_4_-BTC), tris(4-pyridyl)benzene (Co_4_-TPB), tris(4-pyridyl)pyridine (Co_4_-TPP), or tris(4-pyridyl)triazine (Co_4_-TPT). Despite lacking long-range order, void structuring was observed, which allows catalytic functionality to be carried out in liquid solution. By testing the different crystal structure models developed, the PDF data were useful for determining their validity for describing the local connectivity and possible pore morphologies, as shown in Figure 24. The Co_4_-BTC was a near exact match, and the Co_4_-BTB described the features upon elimination of the lowest order Bragg reflections. In the other cases, the pore structure in the samples was shown to be similar to one another, but different from the models. The formation of longer-range oscillations can be an indicator of ordered network formation during synthesis, for example in the crystallization of ZIF-8,^342^ and this should be cross-checked against the development of a corresponding peak in *Q*-space. Likewise, such indications can be further used to check for or rule out the presence of porosity in precursor or intermediate structures formed for instance during the nucleation of MOFs^342,343^ and in MOF liquids and glasses.^334−336,476^ It is expected that such an analysis could be of further use for investigating the local structure and distortion for instance in molecular cages^510^ or other porous liquids.^511^

## 5 Characterization Methodologies

### 5.1 Fingerprinting and Refinement

The identification of certain phases or structural features within a material is one of the most important purposes for diffraction methods.^35^ In reciprocal space, this is generally carried out by qualitative or quantitative assessment of the measured diffraction patterns, from simple visual comparison all the way to refinement of a known structure model. Databases and tools exist specifically for identifying possible phases from observed Bragg peak positions.^512−515^ Diffuse diffraction patterns can be compared to identify differences between the samples, which may result from changes in structure, moisture content, impurities, etc., though very good quality data are required and with limited information accessible about the structure itself.^159,516^

PDFs provide a very sensitive signal for fingerprinting samples, with the additional advantage that crystalline Bragg reflections are not required. Slight but meaningful differences in diffuse scattering, considered as part of a *background* in most reciprocal-space analyses, may codify distinct structural differences between two samples (or sample and structure model). These differences can be studied by various methods in reciprocal space,^464,517^ although the extremely diffuse nature of the signals may lead to poor distinction of different candidates by many metrics.

When no LRO is present, the PDF provides a way to directly access structural properties such as bond lengths in samples where comparison of diffuse diffraction halos may be ambiguous. For similar materials, small differences in bonding character, for instance aliphatic versus aromatic content, can be resolved by shifts in the nearest neighbor bond-length distribution,^139^ or the presence of different ring or cluster structures as discussed in section 4.2. Differences in the molecular motifs present, or in the interaction between them, may generate characteristic signals in the MRO regions that may be identified through comparison to ordered structure models, even in the absence of well-ordered crystalline samples.^137,341,424,518^

A basic method for fingerprinting is to determine the degree of matching between the experimental PDF of an unknown compound and another PDF, which can be measured or simulated from a plausible model. Different metrics can be used to accomplish this. Simple metrics include measures of statistical correlation such as the Pearson product-moment correlation coefficient (PCC), which measures the strength of the linear relationship between two data sets, or Spearman’s rank correlation coefficient, which measures how well the relationship between data sets can be described by an arbitrary monotonic function. The PCC is defined as
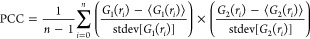
16where ⟨···⟩ denotes the average of the values for the respective function and stdev[···] represents the standard deviation of all data points in the function. It gives a value between −1 and 1, where 1 implies perfect correlation, 0 implies no correlation, and −1 implies perfect anticorrelation. An advantage of this measure is that the resulting value is independent of the scale of each function and simply yields the similarity in the shape of the function, for example slight peak shifting. The PCC can be readily computed using standard statistical packages in Python or R, or for example, by uploading a set of PDFs to the PDF in the cloud Web site (PDFitc.org).^519^

Values around 0.8 or higher have typically been taken to indicate a high level of structural similarity,^38,137,169,172,182^ though this is somewhat arbitrary. A more meaningful analysis may be carried out with multiple data sets, where comparison of PCC values between separate pairs of data sets can indicate better matching with one versus the other.^38,137^ In addition to computing the PCC, visual inspection of the PDF curves as a sanity check should not be overlooked. It should be noted that in the PDF, high PCCs could result between different materials or polymorphs composed of the same molecular species, due to a high degree of match in the intramolecular region. In this case, the coefficient can be evaluated over different ranges to yield information about the similarity of the species, their conformations, and their packing, separately. Deceptively low Pearson coefficient values could result between materials of the same structure when there are large differences in the domain size and degree of structural coherence. However, this can be corrected for prior to the coefficient calculation by applying a damping envelope to correct for the disparity in the curves (see section 4.1).^172^ Different effects can also be mitigated by comparing only certain distance ranges; comparing only the long distance range of the PDFs for instance has allowed for the identification of a suitable molecular packing model out of a large group of candidate structures.^137^

Another similarity metric uses the cross-correlation and respective autocorrelation functions of two data sets and gives sensitivity to comparisons for which shifts in the peaks may obscure actually high structural similarity.^520,521^ Such differences in the PDFs from otherwise similar structures can result from things like slight differences in lattice parameters, molecular orientations, or even conformations. The use of this metric requires a slight transformation of the PDF data set and a weighting function that constrains the calculation of the correlations over a finite range around any given point. This metric therefore gives an additional benefit over the PCC in that it may help to identify apparently bad structural model candidates that might be a good model after structure refinement.

**Figure 25:**
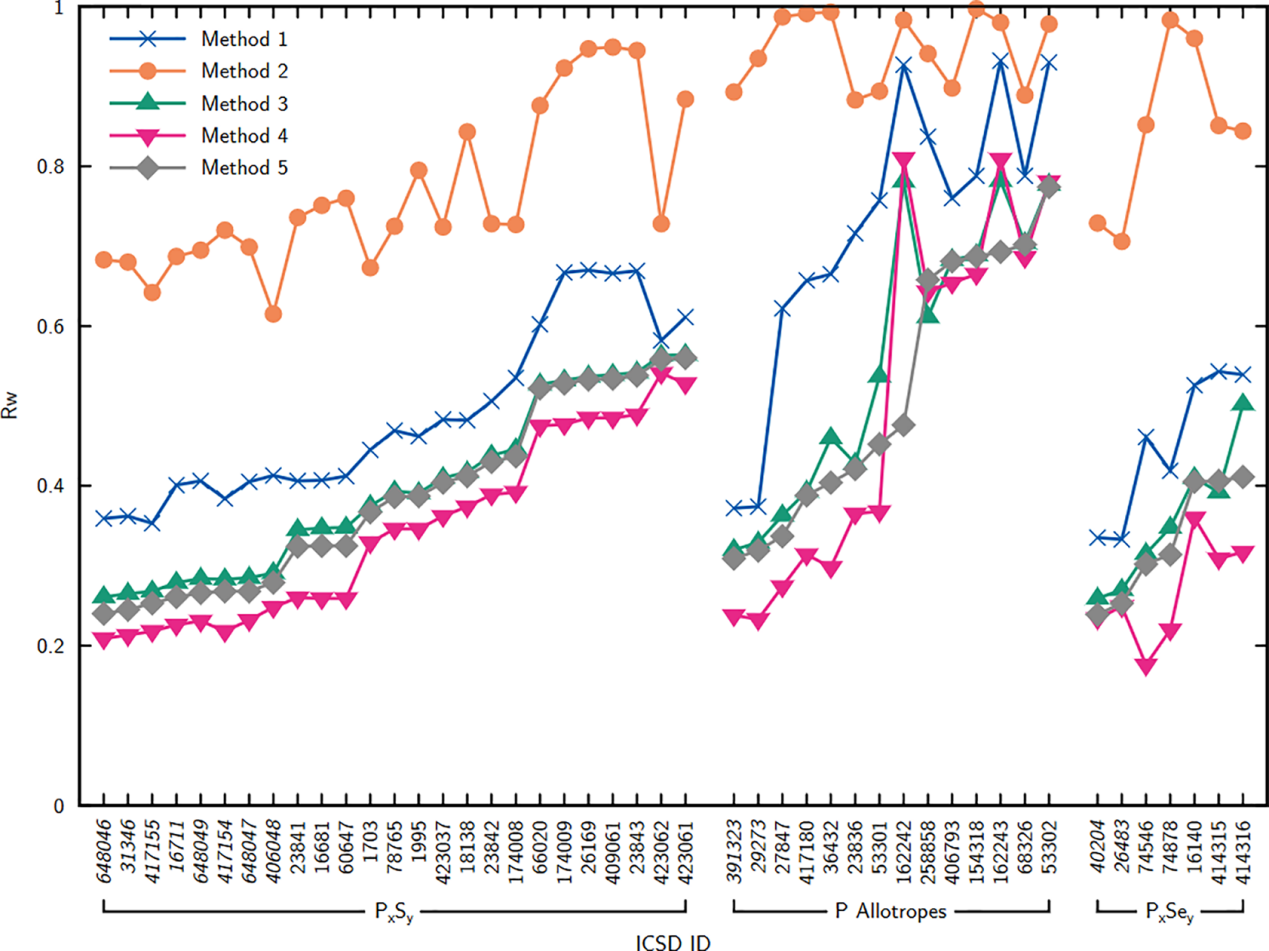
Database mining for structure models: potential models to describe amorphous P_2_S were obtained from the Inorganic Crystal Structure Database (ICSD), including materials with compositions of P_*x*_S_*y*_, P_*x*_Se_*y*_, and P. The models were iteratively fit to the local structure of the PDF data to determine best candidates using different refinement methods biased against the description of the local bonding properties (short distances), biased to the packing of molecular motifs (long distances), or for overall best agreement (full range). Reproduced with permission from ref (424). Copyright 2019 American Chemical Society.

Other goodness-of-fit metrics used in crystallography^522^ are more commonly used for comparison to a PDF simulated from a structure model, for example, after carrying out a structure refinement (see section 6). The weighted profile R-factor (*R*_wp_ or *R*_w_), used in many refinement programs, is defined as
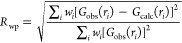
17It measures the absolute value of the discrepancy between observed and calculated profiles with the contribution of each point weighted by the uncertainty of the value, *w*_*i*_ = 1/σ^2^[*G*_obs_(*r*_*i*_)]. Depending on the mode of data collection, determining accurate uncertainties is not always easy,^523^ and an equal weighting can be assumed by fixing *w*_*i*_ = 1. There is no commonly agreed upon standard for what a “good value” should be. Whereas refinement of simple inorganic crystals may give *R*_wp_ < 2.0%, many studies quote values of 30% or more to indicate some structural insights, and it must thus be assessed on a case-by-case basis depending on the assumptions made, the structural features of interest, and the scope of the conclusion. Transue et al.^424^ used a combination of database mining and local structure fitting to determine potential structure models to describe the features observed for amorphous, vulcanized P_2_S, demonstrated in Figure 25. While describing an amorphous material with *crystalline* models will always have inherent issues, this method enables the indexing of certain physical properties, and in this case allowed for the identification of chemical bonding environments similar to P_4_S_3_ and molecular packing properties reminiscent of both P_4_S_3_ and Hittorf’s phosphorus.

An alternative method for “fitting” when no structural information is known, for example, for fingerprinting or quality control purposes, can be performed by fitting a series of peaks.^524^ A model can be generated from a *target sample*, and then other samples can be compared to determine the similarity in peak position and shape. Or, the peak positions could be constrained by the parent phase and then refit to the other sample PDFs to determine how well the structure is described by the target structure.^179^ For highly resolved data of simple materials at short distances, a peak may truly represent a single atom-pair type, and sophisticated ways for extracting pair-distance lists over short distances exist.^524,525^ However, for complex molecular materials, these fitted peaks may comprise a mixture of different pairs whose specific contributions are impossible to unambiguously deconvolute. Thus, the fit only encodes the total signal but not the specific contributions. More specialized use cases also exist, such as the identification of topological features as was demonstrated for the coordination topology of amorphous zeolitic imidazolate frameworks in comparison to models of vitreous SiO_2_ by simply stretching the reference SiO_2_ PDF.^328^

In certain cases, the target is to identify or compare unknown, often amorphous or impurity phases, mixed within a known phase. If a suitable structure model is known, this component can also be modeled by fitting to the high-*r* region of the PDF, then fixing the fit parameters, calculating over the whole range, and subtracting from the experimental signal, for example, to isolate unknown amorphous phases present.^90,526^ However, care should be exercised when the short distance behavior of the pure phase is not well characterized. The short-range, correlated motion behavior of the pure phase should be known to avoid misassignment of features or discrepancies in intensities. Another approach is to subtract a *measurement* of the pure known phase. If the concentration is low, the sensitivity of these methods are much lower since the signal is dominated by the majority structure, and therefore misfit in fitting a model can be detrimental. If the parent phase can be measured separately, without *any* significant structural modification, then it can be possible to directly subtract away its scattering contribution in order to isolate that from the minority component. This analysis generally requires longer measurement times since the statistics measured from the minority component are much lower than from the total sample.^179^

A future direction for fingerprinting and phase matching is through large-scale data mining, allowing for a more comprehensive search for, and identification of, potentially useful models. This has been implemented in the PDF in the cloud (PDFitc) project,^527^ for searching open source databases and in the Crystallography Open Database (COD) and Material Project (MP), for candidate structures.

### 5.2 Structure Solution

For nanocrystalline materials, crystallographic methods focused on Bragg peak analysis are not typically viable for ab initio structure determination. The idea of a unique structure may not even be well-defined for poorly ordered or amorphous materials. In these latter cases, different regions of the sample, or different nanoparticles will have aspects that are common, and aspects that vary from region to region or particle to particle. The measured PDF represents an average over all the variations, unless individual particles are studied, for example, using tomography on aberration corrected TEM images.^6^ Diffraction data reveal aspects of the structure that survive the averaging. This information can be extracted using small-box modeling or large-box modeling methods such as reverse Monte Carlo (RMC) or empirical potential structure refinement (EPSR) that can yield distributions of possible structural properties in the material. These methods will be discussed later in section 6.

**Figure 26:**
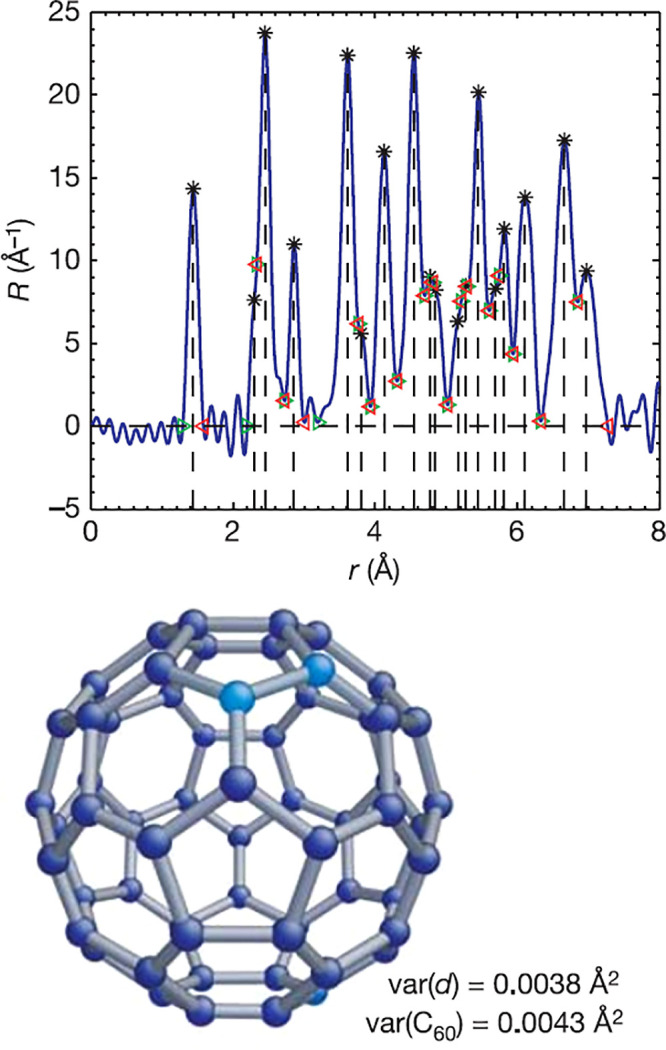
Reconstruction of C_60_ molecule from experimental *R*(*r*) using the Liga algorithm. Figure reproduced with permission from ref (73). Copyright 2006 Springer Nature.

For crystals, which have a mostly unique structure solution, it is often possible to determine that structure from powder diffraction data.^3,528,529^ Since the PDF is the Fourier transform of powder data, it seems plausible that it should be possible to determine structure directly from a PDF, under favorable circumstances. An ab initio determination of the molecular structure has been shown to be possible from experimental data when a sufficient number of distinct peaks are available (i.e., the molecule is rigid with few overlapping contributions).^73^ This was demonstrated for the canonical case of buckminsterfullerene C_60_, using a cluster buildup algorithm, called Liga, developed for the purpose, Figure 26. The algorithm relies on reliable extraction of atom-pair positions from well-resolved peaks, further using the multiplicities based on the integrated intensity in the corresponding RDF in the elemental material (carbon), though was shown to be robust against reduction and even loss of this information.^530^ It uses a competition between candidate clusters, building promising clusters to larger size, and reducing the size of less ideal clusters, based on a score with respect to the list of distances extracted from the PDF.^73,530^ The approach also worked well to determine a number of inorganic crystal structures of compounds and minerals.^530^ Unfortunately, for more complex, low symmetry molecules it failed, due to an inadequacy of information in the signal due to peak overlaps; the inverse problem became ill posed.^531^ Additional information, for example, knowing the number of unique chemical environments in the molecule, further constrains the problem and may remove the ill conditioned nature of the problem.^74^ For molecular systems, the problem can be simplified if the molecular geometry and its conformational degrees of freedom are already known. By fixing the molecular geometry and optimizing only the molecular positions and orientations, structure solutions were accomplished by Prill et al.^176^ The crystal structures for quinacridone, naphthalene, allopurinol, and paracetamol were solved from experimental PDFs given inputs for the molecular structure, unit cell parameters, and guesses for the number of molecules in the asymmetric unit, with or without symmetry imposed on the molecular positions. Given constraints on bond lengths and torsional angles, the conformational flexibility could be parametrized for individual molecules in the asymmetric unit by reducing the symmetry, for example refining as *P*1 with *Z* = *Z*′ = 4, instead of *P*2_1_/*c* with *Z* = 4, *Z*′ = 1. This method appears highly promising, especially given that there is substantial space for improvement, for example by incorporating ensemble-type behavior for orientational or substitutional disorder in the crystal,^532^ or using crystal structure prediction methods to obtain new starting configurations.^533,534^ A brute force method extended from these ideas has been recently demonstrated for the solution of small molecule crystal structures without a priori knowledge of the unit cell or space group symmetry.^535^ This method requires only the molecular geometry, experimental PDF, and a setup of the allowed search-space including, for example, allowed ranges for lattice parameters and cell volume, space groups to be investigated, and any restrictions on molecular position, orientation, or internal degrees of freedom. The proceeding steps then involve random structure generation, evaluation, and refinement, significantly benefiting from fast simulation of the PDFs from the models.^483^ Success of the method was demonstrated for barbituric acid form IV using a range of only 1–20 Å, indicating that it may also work for nanocrystalline materials with structural coherence on the order of only a few nanometers.

Section 4.6 discussed the stacking relationships in layered materials. In some cases, the atomic structure within the layers must also be determined. Even if the layers in the material are not long-range ordered, it is possible to test different structural candidates for the layer motif by comparing only over short distances. Once suitable candidates can be identified, they can then be used for further structure solution by optimizing the interlayer degrees of freedom, for example, interlayer distance and relative orientation as discussed above. For example, in a study of V_2_O_5_·*n*H_2_O xerogel,^390^ fitting to the local structure enabled the presence of bilayers to be identified as opposed to single layers. Then, the bilayer motif was placed into a unit cell with an appropriate interlayer stacking distance and refined over a wider *r*-range, resulting in a suitable model for the material. In a similar way, the structure of a 2D metal carbide (MXene)^383^ was determined for Ti_3_C_2_T_*x*_ where T stands for surface terminating species such as hydroxy ligands.^385^ Here, the starting structures were single layers generated from DFT calculations. The layers were placed into a unit cell with a *c*-axis lattice parameter approximated from the diffraction pattern with the number of layers constrained to two. A suitable model could not be obtained by translation alone, but a good fit could be obtained by a symmetrically allowed rotation of the second layer by 60°. Thus, a suitable space group could be deduced, P6_3_/*mmc*, with a screw axis along *c*, which was further used to constrain the atomic positions in the final refinement. This allowed for further investigation of the effects of intercalation by potassium hydroxide or sodium acetate.

A fundamental difference in structure solution by reciprocal-space crystallographic methods is the importance of first indexing a structural symmetry, which massively reduces the parameter space of the problem. However, in real space, the modeling is often begun without assuming any symmetry (*P*1). Then, attempts are made to build up the model starting with known structural motifs, for example, molecules or secondary building units, while using any knowledge or reasonable guesses about packing distances, orientations, and/or connectivity. Working up from this, MRO structural motifs or even emergent symmetry relationships may be identified in the ensuing models. Since all the information on the diffraction pattern is contained in the PDF (the Fourier transform is a linear transform), it is not implausible to imagine that similar indexing processes could be performed in real space as well. Liu et al.^536^ have approached this idea using machine learning to try and predict space groups directly from the PDF. This may pave the way to better approaches for using crystallographic symmetries to reduce the parameter space for real-space structure solution.

### 5.3 Phase Quantification

The determination of relative phase fractions in mixed phase materials is critical for materials characterization, especially in the preparation of pharmaceutical and food formulations. This includes quantification of different phases of the same molecule (e.g., mixed polymorphic forms persisting during crystallization or amorphous and crystalline phase fractions produced through various processing procedures) or different molecular species (e.g., from phase segregation due to immiscibility).

Various procedures for phase quantification with reciprocal-space analyses are well-developed.^537^ Methods catering specifically to estimating crystalline and amorphous components for example in molecular solids^538^ or partially disordered polymer mixtures^539−541^ have been suggested, depending upon certain measurement conditions and assumptions about the samples. Some phase quantification applications can also be performed using PDF data.

It is useful to reconsider the total scattering structure function in terms of partial structure functions associated with the atoms *i*,*j* situated in separate phases α and β. If the sum over individual molecules in eq 12 is replaced with a sum over atoms of distinct phases, we obtain
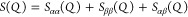
18and a PDF similarly described as a sum of phase-specific partials as in
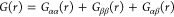
19If it can be assumed that separate phases coexisting in the material do not have any coherent structural relationship, then the cross-phase terms, *G*_*αβ*_(*r*), become zero on average and the observed data are a linear sum of the PDFs of the separate phases *p*, where
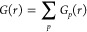
20In general, this is a very good assumption. Only in special cases, where two phases have well-defined relationships to each other, such as through epitaxy, does this assumption not hold.^542,543^ The contribution to the total from each phase (for X-rays) is roughly proportional to the squared number of contributing electrons of that phase within the scattering volume, or more roughly to its atomic number density weighted percent, which is generally rather close to its mass percent. One way to reconstruct the total in eq 20 is by scaling measured PDFs from the pure phases. When standards are measured together with the sample-of-interest, this has the advantage of accounting for experimental effects and systematic errors in the measured PDFs. The mixed phase sample data *G*_mix_ can be reconstructed by optimizing the scale factors of the standards, for example, for crystalline and amorphous data sets *G*_*s*1_ and *G*_*s*2_, by
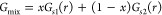
21The concentration, *x*, is varied such as to optimize some metric relating *G*_mix_ and the measurement *G*_exp_.^38,137,177,182,183,544^ A program for carrying out a similar assessment for in situ data has been published.^545^ Accurate quantification relies on a consistent scaling of the standard data sets. Formally, this is accomplished by data processing programs (see section 7.6) that explicitly place the scattering data onto an absolute scale prior to Fourier transformation. When the data are not on an absolute scale, a consistent data scaling must be determined. This could be estimated for instance by fitting a structure model to the standards to determine the scaling or by measuring the density and rescaling according to the −4*πrρ*_0_ baseline. If the samples are composed of the same molecules, it may be sufficient to assume the same density by equating the integral areas of the first coordination shell, because the coordination environments within the molecules should be the same.^38,177,182^ The assumption of equal density is often not bad (e.g., the difference in crystal densities for polymorphs of the same organic molecule often do not exceed 5%)^546^ even for the amorphous forms.^547^ In the cases where the atomic density differences between phases are more significant, then measured or predicted densities might be used to normalize the data to the RDF and normalize by the integral areas of the associated peak.

If the structures of *G*_*s*1_ and *G*_*s*2_ are known, or if there is a suitable model that reproduces the experimental density and fits the PDF, then phase contributions can also be simulated from said models. In this case, the experimental effects must be carefully taken into account. This can be difficult, for the reasons given above about intra- and inter-molecular correlations and variation in conformations. However, if successful, it does have advantages over the summation of standards that scale factors normalized per mass, atomic number, or molecule can be directly extracted (for example, they are listed in the output file of PDFgui), and additional measurements of standards are no longer necessary. In principle, the structural description of a phase that becomes modified in the mixed state can taken into account. Preparing pure phase standards may not be possible, for example when a pure amorphous compound is not stable against crystallization. It is sometimes possible to account for the presence of amorphous phases by computing the PDF for just the molecule itself, and not the full crystal structure. If the molecule is flexible, it may even be better explained by the sum of the PDFs of the rigid subunits of the molecule. The amorphous component in a mixed crystalline and amorphous system is then estimated from the refined scale factors of a two-phase model of the PDF: one calculated from a single molecule and one from the crystal structure of the same material. However, this method should not be expected to give quantitatively accurate values if the amorphous phase has significant MRO. In cases for which significant intermolecular contributions are present in the amorphous phase, one could also consider using MD simulations to develop an amorphous model.

**Figure 27:**
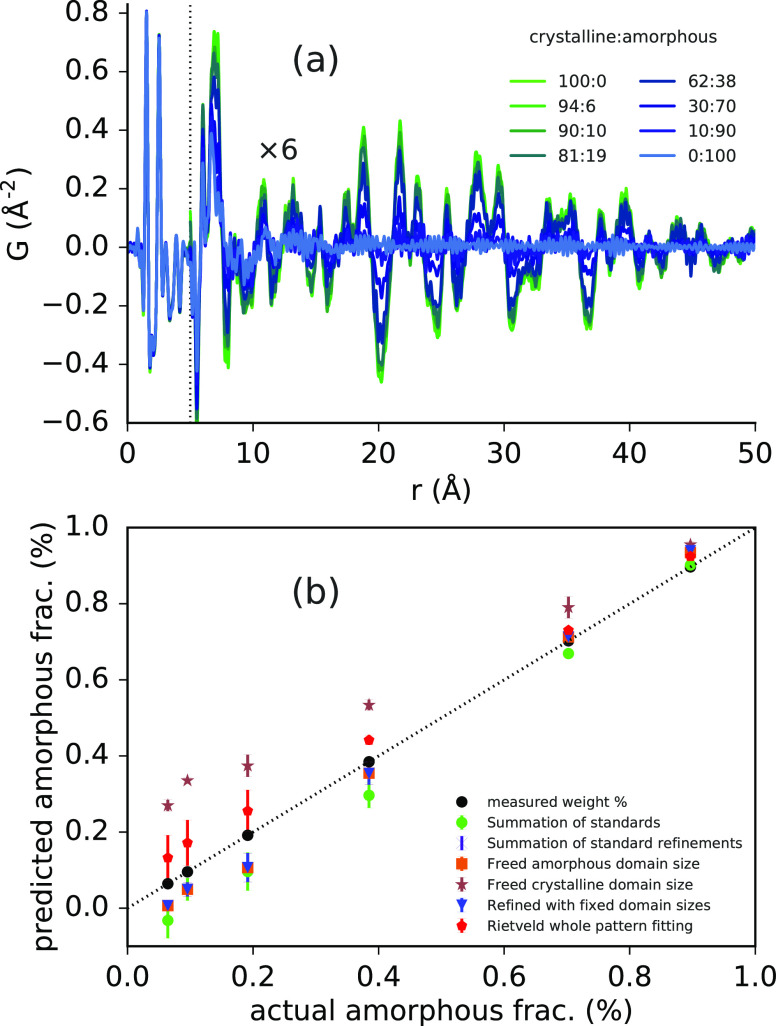
Effect of disordered phase fraction for an HIV pharmaceutical drug, C_48_H_73_ClN_2_O_6_: (a) the peaks due to intramolecular bonding experience negligible change, and the disordered phase does not contribute to the long-range region. A monotonic increase in peak amplitudes due to intermolecular ordering is observed with increasing ordered phase concentration. (b) The results of all phase quantification methods are plotted against the weight percents obtained by massing the separate components prior to mixing. Figure reproduced with permission from ref (177). Copyright 2020 American Chemical Society.

A similar strategy also works if the sample consists of LRO and MRO nanocrystallites. In this case, the MRO component can be approximated using the crystal structure model and damping the longer-*r* correlations to zero (as discussed in section 4.1), using
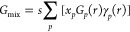
22where *s* is a global scaling factor, *x*_*p*_ is the specific phase fraction for phase *p*, and γ_*p*_ is any modification due to finite domain size and shape. A model for addressing the problem of amorphous and crystalline phase fractions in a two-phase mixture could look like
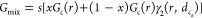
23with the simulated PDF for the crystalline structure model *G*_*c*_(*r*), and where γ is the characteristic function to describe the damping, and *d*_*c*_ is the coherence of the nanostructured or amorphous phase. Different methods to account for the presence of amorphous/crystalline phase fractions were tested for PDF data of a pharmaceutical ingredient and compared to reciprocal-space pattern fitting results, Figure 27.^177^ In this case, the same trend was found for summation of standards versus summations of models, except when the crystallite size of the crystalline model was allowed to vary. The limits of detection and quantitation were determined at 4% and 13% respectively. That these values are an order of magnitude higher than 0.37% and 1%, achieved for whole pattern fitting of diffraction data from a model system of lactose,^538^ may suggest that reciprocal-space analysis is the approach-of-choice when possible. However, the PDF approach may still be semiquantitatively useful in nonideal cases where proper structure models and standard patterns are both not available.

Purely statistical approaches can be used to track the phase evolution as a function of some control variable such as time or temperature. The set of PDFs are considered to be statistically independent and formed into a matrix where each column contains a PDF. This matrix can then be decomposed in different ways, for example, principal component analysis (PCA),^548^ which finds the eigenvectors with respect to the variation in the PDFs, or non-negative matrix factorization (NMF)^184^ that places a positivity constraint on both the components and their weights. PCA is mathematically more straightforward, but NMF results in more physical components.^549^ These methods are used to greatly reduce the number of PDFs that need to be modeled. Instead of doing PDF refinements to possibly hundreds of data sets in the time-series, it is only necessary to refine models to the 3 or 4 or 5 most important components. Every one of the PDFs in the series is then determined by a linear combination of these components, where the weights are output by the NMF procedure. In both cases, results must be judiciously checked for meeting realistic expectations. It is worth noting that, because the matrix decomposition methods are determining the smallest number of components that captures the most variance in the data, they are monitoring what is *changing* in the data as a function of time. For example, if the experiment is to monitor an in situ reaction, a number of reagents react to produce the product, or a number of products. In this case, the NMF components will reflect the reaction equation; in other words, one of the components will yield a linear combination of the PDFs of the reagents with weights given by the balanced stoichiometric reaction equation, and the other will give the same for the products. There is a lot of open space for improvements in the application of these methods, particularly in terms of protocol standardization.

### 5.4 Component Miscibility

The understanding of intermolecular interactions is pivotal in both cocrystal and pharmaceutical formulation design.^550−552^ If the components of a mixed-phase system are highly miscible with each other, then eq 20 is no longer a good assumption, because a new scattering contribution arises from the restructuring of mixed species. A prominent problem in pharmaceutical and polymer processing, is the ability to determine the level of solubility between solid phase components. The driving force to mix hinges on the formation of new, preferred structural states involving both components. This can strongly impact the observed properties and stabilize individual components against product-degrading transformations. This has been a primary focus for much PDF research carried out in the pharmaceutical literature, for example, for the study of amorphous pharmaceutical-polymer blends.^185−193^

Considering a mixture of drug (D) and polymer (P) molecules, eq 18 can be reformulated into component-specific terms as
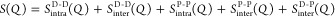
24

The target for the experimental analysis is to determine whether the cross-component terms *S*_inter_^D–P^(*Q*) are present, and hopefully gain some insight into the nature of these structural states. Unfortunately, the accurate extraction of this term is not trivial. One method to do so has been demonstrated for a compositional series of itraconazole and polyvinylpyrrolidone (PVP).^194^ The process requires first analyzing the measured X-ray diffraction patterns and identifying compositional ranges for which isosbestic points exist (or do not exist) in relationship to the pure phase patterns. Ranges where new isosbestic points develop that are inconsistent with the pure phase patterns may suggest new structures developing from interactions between drug and polymer and the development of a region of new phase stability in the system. If the structure of the polymer is not strongly affected by the presence of the drug, *S*_intra_^P–P^(*Q*) + *S*_inter_^P–P^(*Q*) may be approximated and subtracted using the measurement of the pure polymer. If the drug disperses well, or in cases of dilute drug concentrations, it may also be possible to consider drug–drug interactions *S*_inter_^D–D^(*Q*) as negligible. Finally, *S*_inter_^D–P^(*Q*) can then be extracted by subtracting *S*_intra_^D–D^(*Q*) simulated from a model of a single drug molecule, as discussed in section 4.2. Note that this requires a suitable molecular conformation, which may not necessarily be well-represented by the crystal form, and thus conformational searches and comparison of simulated and experimental *S*(*Q*) may be useful for identifying an optimal model.

**Figure 28:**
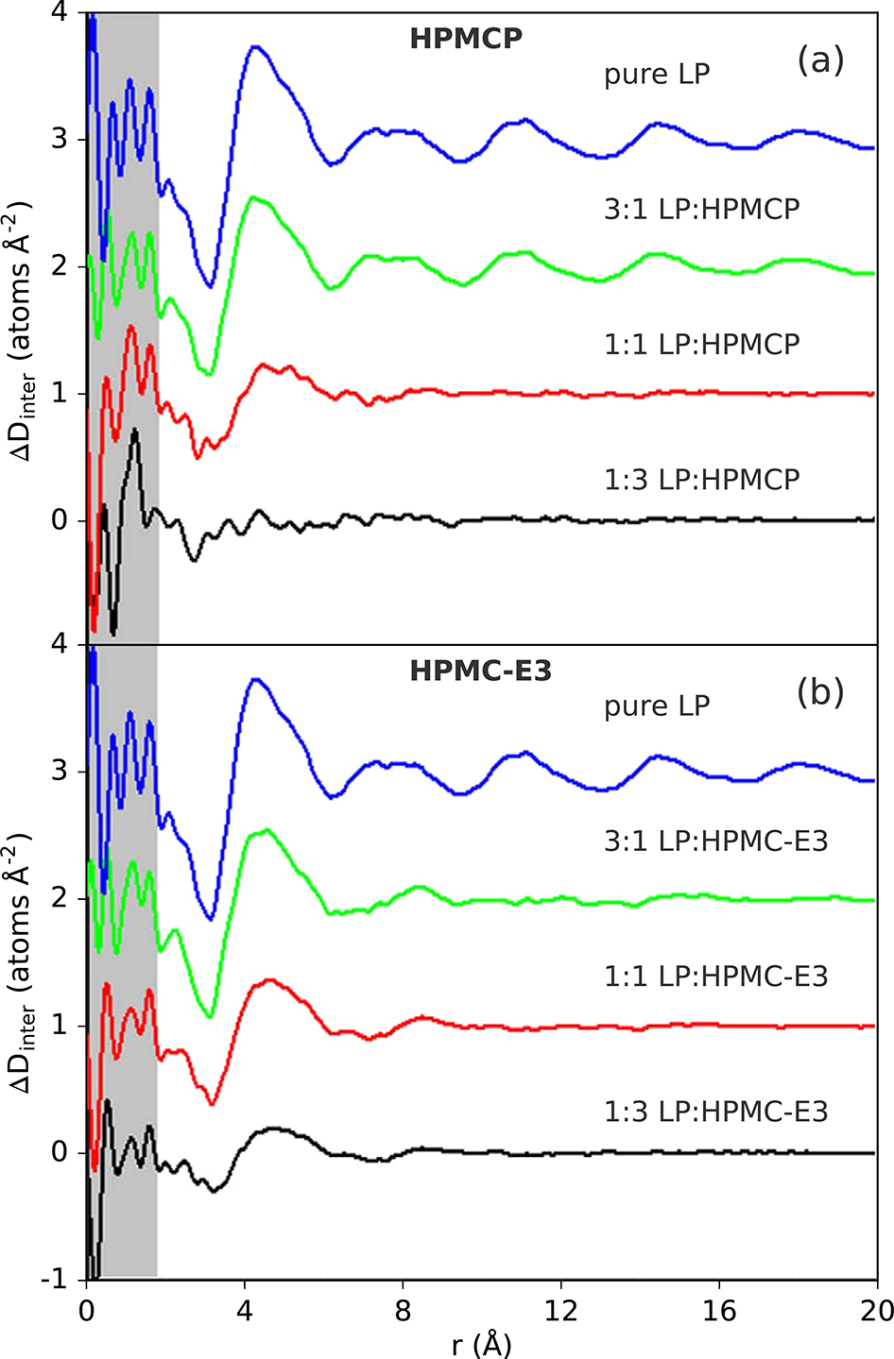
Extraction of intermolecular dPDF including drug–drug/drug–polymer correlations for dispersions of lapatinib (LP) with (a) hypromellose phthalate (HPMCP) and (b) hypromellose (HPMC-E3) at ratios of 1:3, 1:1, and 3:1. Decreased intermolecular correlations for 1:1 and 1:3 LP:HPMCP for instance were noted to correlate with salt formation and increased stability. Here *D*(*r*) is a slightly different normalization of *G*(*r*).^31^ Figure adapted with permission from ref (195). Copyright 2017 Springer Nature.

A similar assessment was utilized by de Araujo et al.^195^ to assess the mixing and associated stability of dispersions of the active pharmaceutical ingredient lapatinib within hypromellose phthalate (HPMCP) and hypromellose (HPMC-E3) at ratios of 1:3, 1:1, and 3:1. Equation 24 can analogously be written in real space as
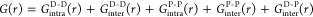
25and in this case, *S*_inter_^D–D^(*Q*) or likewise *G*_inter_^D–D^(*r*) could not be neglected. A subtraction of the reference polymer measurement *G*_polymer_ from the drug loaded polymer measurement *G*_loaded_ results in a difference function given as
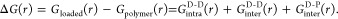
26Further subtraction of the intramolecular drug component in reciprocal or real space results in only the intermolecular components. In either case, the partial signals resulting from these differences are commonly referred to as the difference PDF (dPDF). By comparison to *G*_inter_^D–D^(*r*) of the pure drug, signals corresponding to some orientational correlations between either (or both) drug molecules or drug and polymer remain and lead to lower stability against lapatinib recrystallization. Salt formation between lapatinib and HPMCP resulted in reaction and a homogeneously disordered dispersion of the drug, suggested by a lack of intermediate-range structure in the dPDF signal, Figure 28. A similar assessment of polymer dispersions with the antiparasitic drug flubendazole was also carried out.^553^ In these implementations, the software XISF^464^ was used for fitting intramolecular components in *Q*-space, though similar methods in real space can be implemented using, for example, Diffpy-CMI^480^ to calculate single molecule PDFs using the Debye equation method.

Another method for determining the presence or absence of significant intercomponent interactions is to reference X-ray scattering and PDF data to the pure component patterns. For phase separated systems, the PDF is assumed to be an incoherent sum of drug–drug and excipient–excipient interactions, and it should be suitably fit by a linear summation of reference patterns from the individual phases. In highly miscible systems, the fitting of standards should not suffice due to the third intercomponent interactions between drug-excipient. This should lead to a poor fit from the reference data or scale factors that deviate significantly from known weight percentages.^185^ When reference patterns for individual components cannot be obtained, for example when an individual component will not form a glass on its own, a similar method to PCA termed the Pure Curve Resolution Method (PCRM) has be used. Dispersions are measured over a range of mass ratios and a statistical analysis is used to extract reference patterns from the variance in the data.^187,188^ There are significant benefits to performing these measurements over a wider *Q*-range, in that near-neighbor and intramolecular pair-correlations can be resolved, and there is less error due to termination effects.

A further approach to extracting intermolecular interactions, rather than the reference methods described, is to holistically model the interacting system. However, this method requires more assumptions in building and constraining a starting model, especially where structural details of amorphous excipient materials are unknown. It may be more feasible for studying intermolecular modifications in single component systems, for example, modifications to hydrogen bonding structure,^198^ though support from other techniques is increasingly critical.

### 5.5 Impurities and Degradants

It is important to detect and understand the presence of impurities in functional materials. Unlike solid dispersions mixed with known components, the nature of the impurities may be unknown. They may be present in the starting materials or inadvertantly produced through reactions or degradation. At high concentrations of segregated phases, impurities may be assessed by some of the indexing methods described above, for example to determine inorganic degradation phases in MOFs,^367,368^ in either the diffraction pattern or PDF. If crystalline, impurity components may be identifiable at well below 1 wt %.^179,180,554^

Identification strategies become more difficult for impurities dispersed at low concentrations within the sample-of-interest. Ideally, if the impurity composition contains different elements, especially with higher scattering power (e.g., higher *Z* elements for X-rays), then PDFs may show altered relative intensities or new peaks from different bond lengths at short distances. It is typically not possible to assign the chemistry of a peak from the distance alone, so other methods such as various spectroscopies (FT-IR, NMR, EDX, etc.) could be helpful.

Molecular impurities can be observed in the form of moisture from the atmosphere, other solvents from processing, or degradation products.^159^ In these cases, additional analyses may be necessary, via, for example, chromatographic, thermal, or mass-spectrometric methods. If the impurity composition is very similar to the sample-of-interest, or the weight-percent is too low, other effects may be observed in the PDF. For example, when sucrose is melted, degradation to glucose and fructose and formation of oligomers has little effect on the intramolecular bond distribution, but the presence of the mono- and polysaccharide compounds modifies the intermolecular distribution of pyranose and/or furanose rings, which can be observed by changes in the broad density modulations from roughly 6–16 Å.^204,205^ This is accompanied by systematic changes in the observed diffraction patterns, and it is therefore critical to assess the role of impurities on the diffraction patterns of amorphous materials.^159^ Residual moisture/solvent can also have a significant effect on the diffractoion patterns and PDFs.^177,197^ Signals in the PDF could be observed and extracted from dichloromethane leftover from microfluidic processing around 4 wt % in an amorphous HIV drug formulation by subtraction of the PDF of the ball-milled amorphous form.^177^ Complicated situations with distributions of different species with similar bonding properties, such as different thiophosphate anions discussed in section 3.4.3, may require further standard measurements in order to benchmark the local bonding properties, for example, intramolecular peak widths and relative intensities of pure species, to have any chance of indexing the presence of the components in mixtures.

### 5.6 Incorporation and Binding

Target applications for nanoporous molecular materials include nanoscale sieves or filters to take up, store, and/or neutralize certain ionic or molecular components. This encompasses a wide variety of so-called *host–guest* compounds with uptake of noncovalently interacting guests into, for example, inclusion (molecular host cavity or lattice of channels/tunnels), intercalation (laminar host structures), and clathrate (host-cage or lattice of host molecules) compounds. Many incorporation processes do not result in crystalline guest-structures, leading to diffuse, difficult-to-interpret effects on the diffraction pattern. However, an understanding of the adsorption processes, and the resulting structural states at the atomic scale, is required to improve selectivities. The goal in total scattering studies is therefore to isolate and interpret the scattering contributions from the localized guest structuring.

The approaches discussed in sections 5.3 and 5.4 can be used for a system with host (H) and guest (Gu) structures. If the scattering contribution or real-space structure of the host can be adequately modeled and removed, then the dPDF, in this case the difference between the guest-loaded PDF, *G*_loaded_(*r*) and the bare host, *G*_host_, can be written as
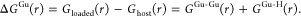
27In other words, the dPDF contains information pertaining to the structural relationship predominantly between the guest and host material, but also any guest–guest correlations that survive the averaging. For certain elements, the dPDF can be formally accessed experimentally through the use of anomalous differential X-ray scattering (section 7.1.1) or through isotopic substitution with neutrons (see section 7.1.2). A routine for extracting partial PDFs from combined X-ray and neutron data, MIXSCAT, has also been proposed.^555^

**Figure 29:**
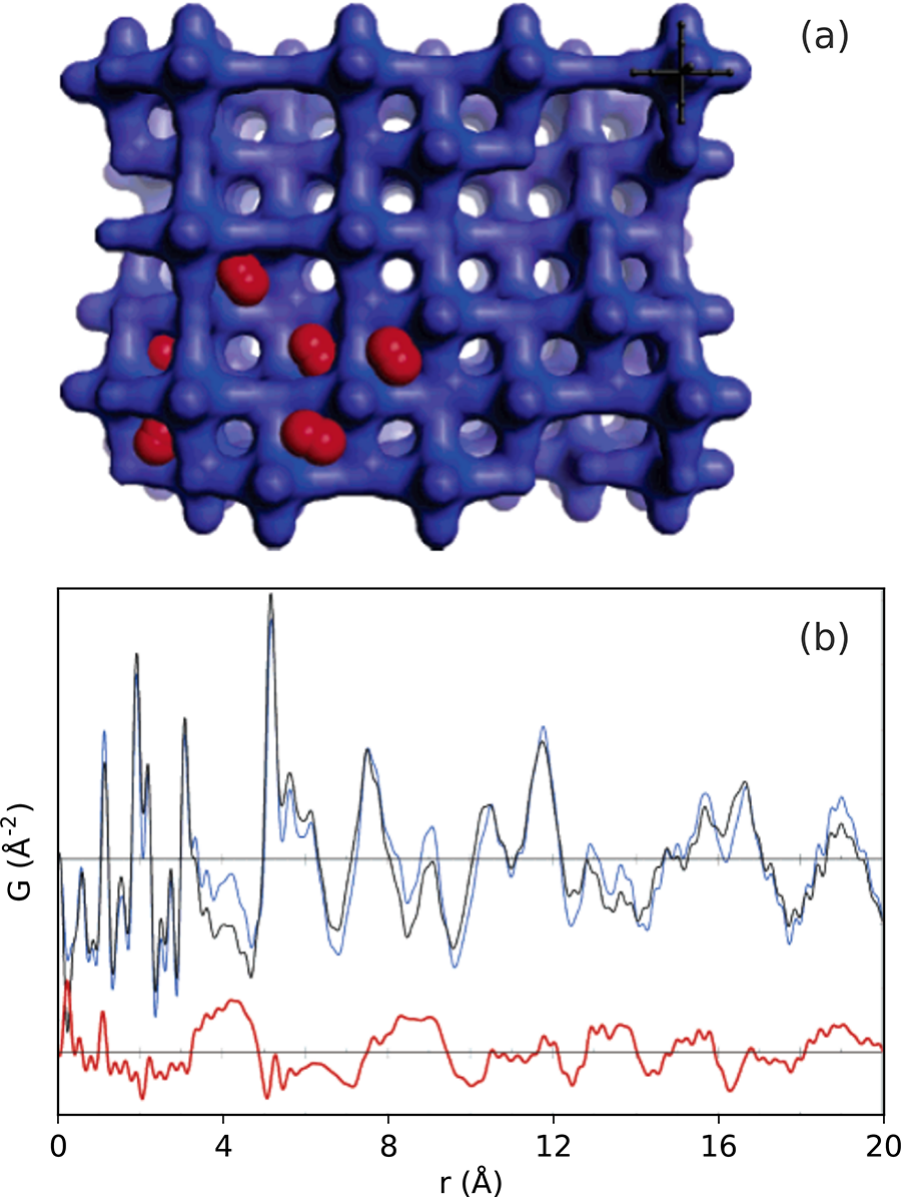
Examples of guest structuring accessed via difference PDF analysis: associations of disordered N_2_ gas with itself and the host framework within the pores of Prussian Blue Mn_3_[Co(CN)_6_]_2_·*x*{*N*_2_}. Adapted with permission from ref (344). Copyright 2015 American Chemical Society.

Another form of the differential PDF may be accessed using two measurements, one of the host material as-is and the other with it loaded with the guest. The subtraction of the two signals in this case also gives Δ*G*^Gu^(*r*) when the structure of the *host* component does not change. This can be a good assumption when loading levels of the component of interest are very low, the interactions between guest and host material are relatively weak, and/or the host material is very rigid. An example of extracting the *guest* dPDF using this principle for N_2_ gas within Prussian Blue is given in Figure 29. If the host structure does change, then *G*^H–H^(*r*) cannot be formally removed in this way. The modified host structure must then also be accounted for using a model or also possibly by applying modification operations to the empty host data set (expansion, contraction, broadening, scaling, damping, etc.).^369^

A weighted difference approach to extracting the local structure of Cs^+^ cations within zeolite ITQ-4 was motivated by the survival of the silicate framework local structure when intercalated by varying concentrations of Cs^+^.^556^ Upon signal extraction, intrinsic structural features could be directly observed, such as a distinct coherence length of approximately 13 Å, and the nearest neighbor Cs bond distances of approximately 4 Å. Further, models incorporating the Cs ions into the host structure in various ways could be tested and ruled out, showing that locally ordered zigzag chains of Cs^+^ ions form along the 1D channels in the zeolite. PDF coupled with Raman measurements could also identify functionalization of Hg by thiolate sulfur bridging within mesostructured silica.^557^ A similar data treatment and analysis involved determining how Tb^3+^ ions load between Zr-phosphate layers linked by the phenyl-phosphonate groups.^371^ By subtracting the scattering from the empty host-structure, prior to Fourier transformation, a distinct and well-defined local coordination environment for the Tb ion could be determined. This was done by assembling a library of known, predicted, and closely related structures of TbPO_4_, then simulating the Tb-associated dPDFs using PDFgui. In another example, a detailed investigation of [Fe(CN)_5_NO]^2–^ anions within mesoporous silica allowed for identifying models for their orientations within the pore and chemical association with Na^+^ cations.^558^

The loading of molecular gas and liquid species into microporous hosts has also been studied, for example, the substructures of H_2_ and N_2_ loaded into Prussian Blue analogues.^344,345^ H_2_ showed a disordered, nondirectional orientation distribution within the pore, without any direct binding to Mn^2+^ sites, whereas different adsorption sites with varying occupation were observed for deuterium loading into IRMOF-1.^347^ The confinement driven structuring has also been studied for various species within nanoconfined graphitic pores.^559−563^ I_2_ incorporation into ZIF-8 for radioiodine capture and storage has also been investigated, showing that I_2_ loading is consistent between crystalline ZIF-8 and after amorphization, which helps to inhibit subsequent release of the guest molecules.^348^ The binding behavior of I_2_ was found to change with increasing loading concentration, further leading to a loss of long-range order in the host.^349^ A study of hydrogen sulfide (H_2_S) binding into the metal–organic framework CPO-27 showed directional coordination of H_2_S to Ni sites by a Ni–S peak at ∼2.55 Å as well as binding at coordinatively unsaturated sites left after dehydration.^350^

The binding associations of different compounds and complexes with Zr_6_O_8_ clusters have also been investigated in a variety of MOFs. These associations can typically be identified by the appearance of peaks at the distance between the most strongly scattering species of the guest component and the Zr atoms of the MOF node, such as for Sb(OH)_6_^–^,^564^ selenate/selenite anions,^352^ InMe_3_,^354^ PS_4_^3–^,^565^ and dimethyl methylphosphonate.^369^ In many cases, any distortion to the rigid Zr-oxo clusters appears negligible, allowing direct extraction of the dPDF of the binding associations and comparison to distances expected for different binding models. Interestingly, apparently stronger interactions associated with structural modifications such as the bridging of Ni-hydroxo clusters^326^ can lead to significant distortions, as observed in temperature-induced polymorphic transitions of both Zr-oxo- and Hf-oxo-clusters.^325^

### 5.7 Interfacial Modification

In select cases, the rearrangement of solvent molecules at interfaces has been studied by total scattering and PDF methods.^281^ Grazing incidence wide-angle X-ray scattering (GIWAXS) has been applied to study the arrangement of water molecules at the liquid–vapor interface.^566^ By tuning the beam to sufficiently low incidence-angles, total scattering structure functions could be obtained corresponding only to the interfacial region. The inability to describe a shift in the first sharp diffraction peak with respect to measurements of bulk water by geometrical effects suggested an expansion of the molecular packing near the surface, in agreement with prior ab initio molecular dynamics simulations.^567^

Comparison methods using so-called double-difference PDF analysis have been used to investigate the rearrangement of solvent molecules around dilute, suspended nanoparticles. This was first demonstrated for various nanoparticles dispersed in methanol, ethanol, 1-propanol, and *n*-hexane.^568^ These suspensions also show characteristic shifts in the first sharp diffraction peak that suggest modifications to the solvent behavior. In their analysis, scattering contributions from the bulk solvents were subtracted from the suspensions, and the resulting PDFs were found to consist of signal from the dilute nanoparticle structures with additional damped sine-wave-like oscillations suggesting the modification of layers of solvent molecules due to the interaction with the particle surfaces. The modulations were found to scale with the chain length of the solvent with reordering up to distances of 2 nm. Similar analysis in subsequent work suggests that water molecules rearrange into adsorbed and further hydration shells at faceted surfaces for different nanoparticles and capping agents.^281,569^

Solid interfaces can also govern material properties. The interfacial structuring of sunlight-harvesting dye molecules and semiconductor materials is important for the function of dye-sensitized solar cells. A recent study has suggested a method for probing the structural modifications associated with different dye binding modes at TiO_2_ interfaces through difference analysis (Δ*G*_sensitized_(*r*) – *G*_unsensitized_(*r*)).^570^ While the data did not appear to strongly discern between the proposed models, the qualitative agreement in peak positions shows promise for indexing such structural states and possibly to provide an avenue to determine deficiencies in simple DFT-derived models versus real interface structures. The NMF approach discussed for phase analysis in section 5.3 has also been recently demonstrated for extracting the interfacial signal from an experimental set of PDFs.^571^

### 5.8 Density Distributions from Small and Medium Angle Scattering

Longer length-scale density modulations appear in the low-angle region of the scattering pattern and are studied using small angle scattering (SAS). Having this scattering information available can be useful for studying things such as the distribution of larger, secondary molecular motifs or layers, the distribution of separate phases or domains, or the distribution of particles themselves, rather than their internal atomic structure. Peaks observed for instance in paracrystalline and semicrystalline polymers (including thermoplastic polyurethanes) are often attributed to microphase segregated crystalline and amorphous (or hard and soft) regions, often in the form of lamellar stacks^572−575^ or a random close-packed or liquid-like distribution of segregated domains.^576^ For a 3D isotropic material (e.g., random close-packed or liquid-like), the position of the peak in the observed SAXS intensities, *I*(*Q*), can be related to the interdomain spacing *d* by the Bragg relationship (i.e., *d* = 2π/*Q*).^577,578^ Discrete objects are also routinely investigated, for instance the size, conformation, and even interactions between biological macromolecules in solution.^579^

SAS data are commonly assessed by various forms of real-space distribution functions that come with different requirements for the validity of their use and varying benefits for assessing different physical properties of structural heterogeneities.^573,580−583^ The most common forms are the generalized 3D and 1D correlation functions. These functions represent the longer-range fluctuation in the density with respect to the average and are related to the probability that a rod of length *r* will have both ends in the same phase. The 3D correlation function corresponds to the case of isotropic materials, and can be calculated by
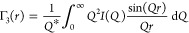
28where Q* is the SAS invariant, defined by Q* = ∫_0_^∞^*Q*^2^*I*(*Q*)* *d*q*. The 1D correlation function corresponds to the case of scattering from orientationally averaged 1D stacks and can likewise be calculated from
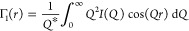
29These functions can be assessed for measured data using for instance the program SasView.^584^ Other common real-space functions used to assess SAS data include the chord distribution function, *g*(*r*) (3D isotropic case),^580^ and the interface distribution function, *g*_1_(*r*) (1D stacks),^573,574^ which are respectively related to the second derivatives of the 3D and 1D correlation functions. Density distributions from SAS signals are commonly used to assess morphologies of discrete macromolecular and nanoparticulate structures in dilute suspension,^585,586^ This can aid in determining macromolecular conformations such as cis versus trans conformations of dumbbell shaped polyoxometalate-organic hybrid molecules in dimethyl sulfoxide solution.^587^ Real-space assessment of coherent SAS interferences between particles or phases is also possible, for example, to investigate distributions of lamellar or secondary molecular structure motifs.^153,588^

The particular formalism used by the atomic PDF can be extended to the SAS regime in cases where certain scattering properties for the scattering entities are satisfied. The total scattering structure function can then be described by
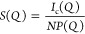
30where *P*(*Q*) is the orientationally averaged intensity resulting from the form factor for the particular scattering motifs.^589^ This approach has been developed as a particular application for studying the spatial arrangement in assemblies of nanoparticles in real space.^590^

**Figure 30:**
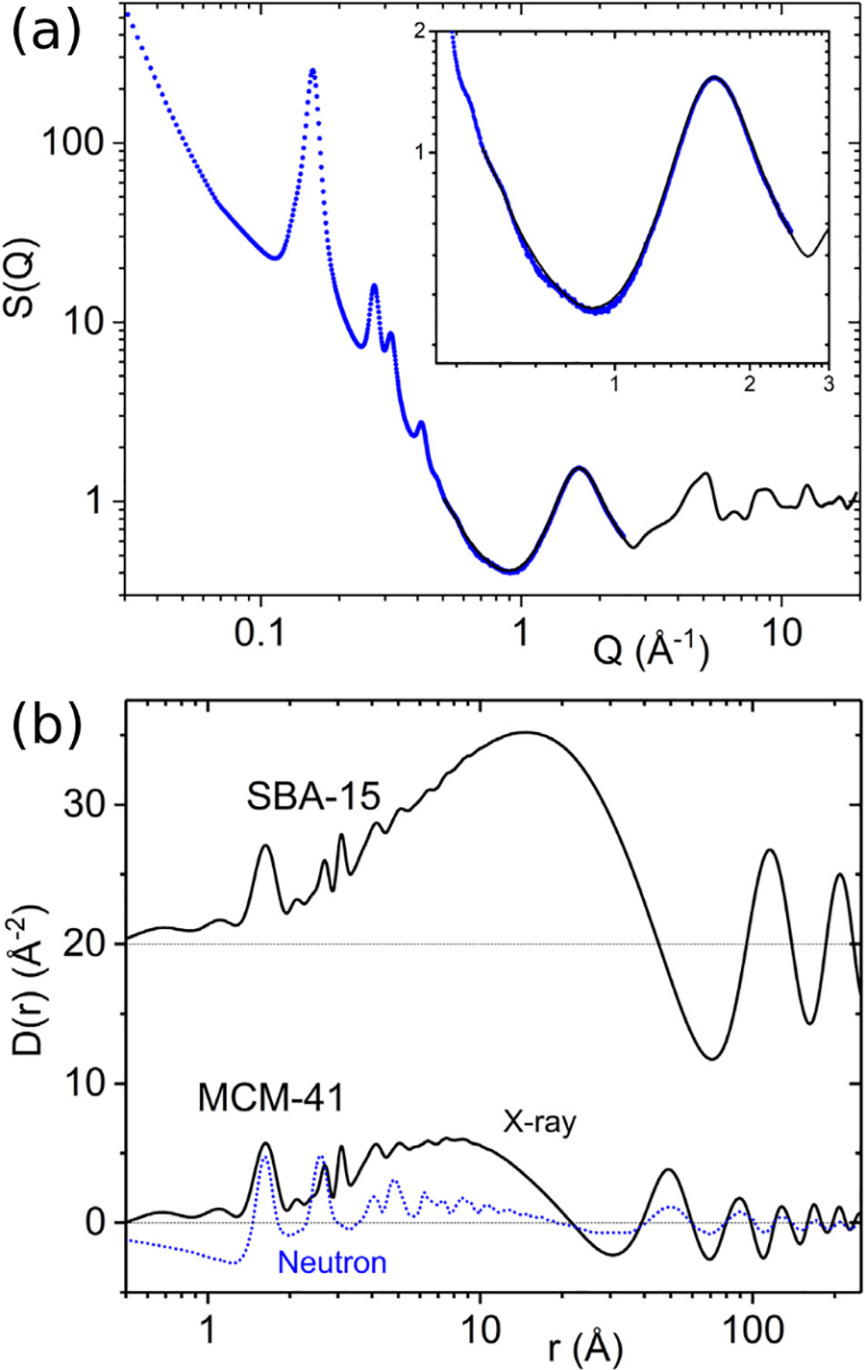
Extended-range PDF: (a) the overlap region in *Q*-space between the SAXS and WAXS detectors shown for the case of ordered mesoporous amorphous silica MCM-41, and (b) the extended range X-ray PDFs for amorphous mesoporous silicas SBA-15 and MCM-41 (solid black curves). In the lower part of the figure, the X-ray PDF of MCM-41 is compared to the published neutron PDF (blue dotted line, scaled for clarity). Adapted with permission from ref (591). Copyright 2020 Elsevier.

Absent a priori knowledge of the scattering behavior of the motifs, Benmore et al.^591^ have recently proposed a method for stitching the SAS signal onto the wide-angle, total scattering signal at the *I*(*Q*) level, and then propagating forward to real space. This is no longer the standard atomic PDF, *G*(*r*), which does not include the SAS signal by definition,^33^ but a modified form that carries additional, lower-frequency density modulations associated with long-range morphological or phase inhomogeneities, as shown for mesoporous silica samples in Figure 30. While this approach has not yet been applied to further scientific use cases, it is expected that these measurements could provide an experimental reference for testing the validity of large-scale, molecular dynamics simulations or other hierarchical models.^592−595^ Total scattering and the associated extended-range PDF, including the relevant SAS regime, could be generated using the Debye formalism and validated against the experimental data. Given sufficient computing power, this could theoretically be performed for any size model and simulation time-scale. It could be particularly useful for applications to phenomena including phase segregation, nucleation, or the study of composite materials with mesoscale inhomogeneities.

**Figure 31:**
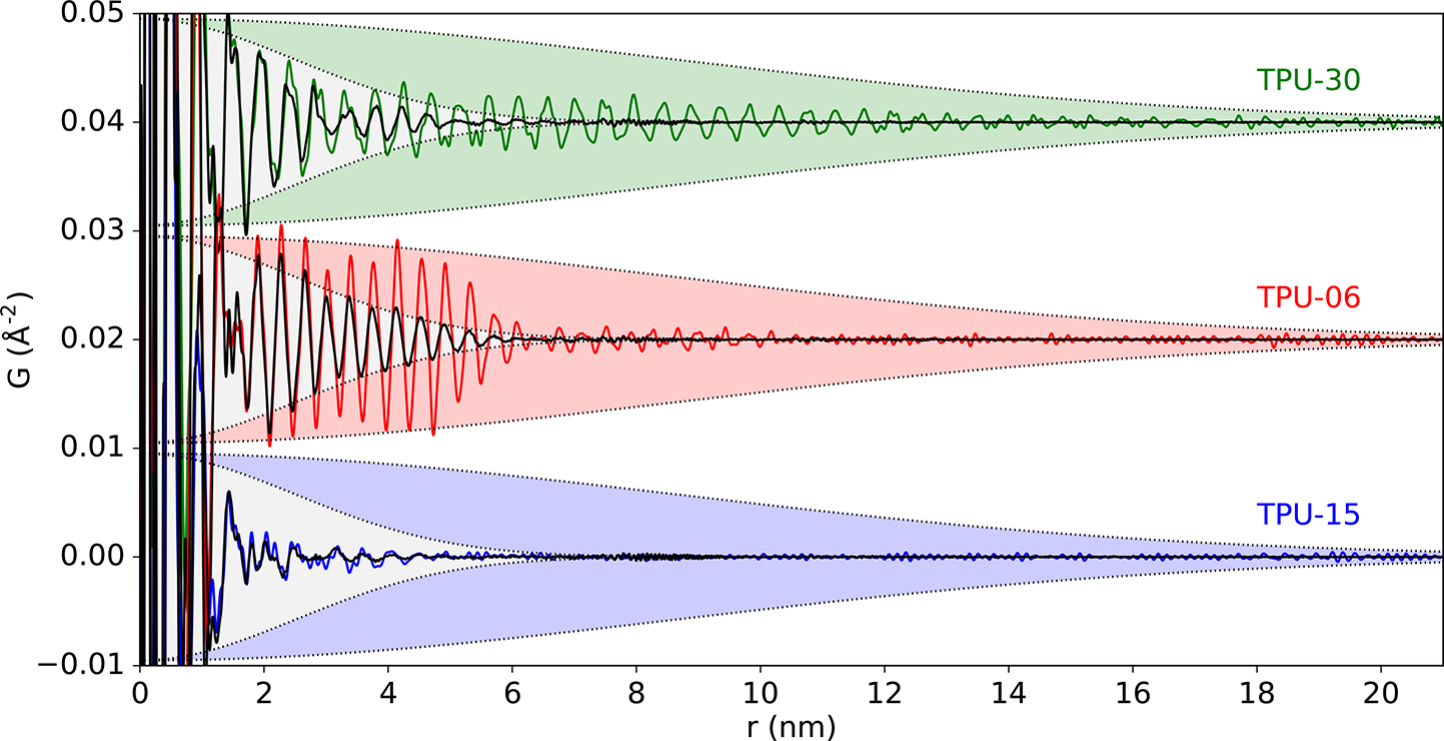
Intermediate and long-range chain packing density fluctuations: long-distance range of PDFs from RAPDF experiment (black) and from higher *Q*-resolution experiments (color) showing structural correlations over an increased distance and substantial differences in damping profiles due to differences in domain size and shape distributions as confirmed by TEM and AFM measurements. Reproduced with permission from ref (139) under CC BY 4.0 license.

The medium-angle scattering (MAS) regime (roughly 0.1–5.0 Å^–1^) generally contains the information about density fluctuations associated with the packing of chain, layer, and/or pore motifs as discussed in sections 4.5, 4.6, and 4.7. Larger thermal displacements and conformational disorder often lead to negligible distinct atom-pair correlations across motifs, and so use of data with a lower *Q*_max_ does not substantially degrade the real-space signal at long distances (a caveat being that the SRO cannot be accurately assessed with such data). High *Q*-resolution measurements of this MAS region can be useful for extracting the density distribution of these structures over larger distances. This allows for less ambiguously discerning particular packing structures and differences in both coherence length and morphology. The PDFs (in this case lower resolution density distributions) obtained from high *Q*-resolution Cu Kα_1_ data are compared to atomic PDFs obtained from typical synchrotron measurements of different thermoplastic polyurethanes in Figure 31. The power of this approach lies in the increased ability to differentiate uniform amorphous versus nanocrystalline structures as well as to determine differences in the damping profiles that correlate with both particle morphologies and size distributions observed from microscopy.^139^ This is an important consideration when measuring microporous MOFs and COFs, which may show strong Bragg reflections at much lower *Q* values than typical condensed crystal structures. For the assessment of low frequency structural oscillations at high distances, the effects of the *Q*_min_ should also be assessed, see section 7.4.

### 5.9 In Situ Structural Transformations

The structural effects and characterization methods discussed thus far give a useful toolset for analyzing systems over the course of exposure to some stimulus and ensuing transformation or reaction. Thus, by detecting and following changes in the conformation, contacts, packing, degree of order, etc., one can begin to determine directly the associated atomic-scale structural mechanisms. In addition to ex situ modalities, it is becoming increasingly common to study transformations in situ, typically with high energy, high flux X-rays and complex sample environments.

Variable-temperature studies are fundamental to studying phase transformations and properties such as thermal expansion. The combination with total scattering is particularly useful when the structural development has some noncrystalline component such as with order–disorder transitions. For example, cyclohexane displays orientational disorder in the high symmetry, or plastic crystal state,^596,597^ where the molecules are rotationally disordered on their lattice points.^488^ However, based on RMC modeling (section 6.2), the persistence of short-range orientational order of the molecules in the plastic phase was observed. Symmetry-reducing local distortions of the symmetric M_6_O_8_ (M = Zr, Hf) octahedral clusters were observed within MOF crystals of NU-1000 and UiO-66.^598^ The distortions could be likened to monoclinic to cubic transitions in bulk ZrO_2_ and HfO_2_ crystals. However, the distortions were observed to occur on heating between approximately 100–200 °C, rather than cooling down from ∼1170 °C (ZrO_2_) or ∼1750 °C (HfO_2_) for the bulk crystal phase. Interestingly, the distortions have no observable effect on the symmetry of the MOF crystal. Such behavior could hold potential for tailoring the electronic state of the active sites, allowing for instance tuning of the catalytic behavior under particular operating conditions. Local distortions in metal–organic compounds can also lead to interesting thermal behavior. Chapman and co-workers investigated the local mechanism of negative thermal expansion (NTE) in Zn(CN)_2_ on heating from 100–400 K.^599^ PDF analysis indicated that an increase in transverse displacements of cyanide bridges between Zn centers allows for an overall contraction of non-nearest neighbors despite expansion of nearest neighbor bond length. Slight NTE was also observed in a variable-temperature PDF study of Zr-UiO-67.^369^

**Figure 32:**
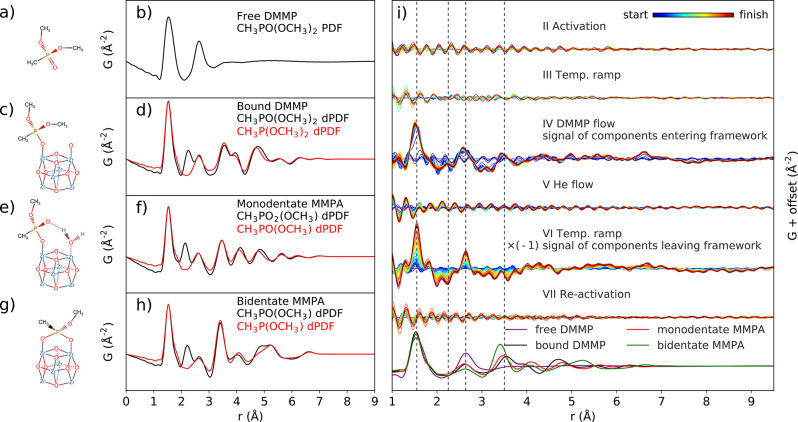
A demonstration of in situ, differential PDF (dPDF) to track the presence of DMMP gas and its binding and degradation products with respect to Zr-oxo clusters: schematic representations with corresponding simulated PDF signals for (a,b) a free DMMP molecule, (c,d) monodentate bound DMMP, (e,f) monodentate bound MMPA, and (g,h) bidentate bound MMPA. Black curves show the dPDF corresponding to DMMP–DMMP plus DMMP–cluster pair distances with the bridging oxygen considered as part of the DMMP molecule (red curves with bridging oxygen considered as part of the cluster). The experimental dPDFs are shown in (i) for the different activation, DMMP dosing, and reactivation steps, with respect to the structure at the beginning of each step and compared to the simulated dPDFs. The structural differences are negligible for most steps, except for the increase in gas signal with flowing DMMP, and the subsequent removal of DMMP during heating. The presence of a peak at ∼3.5 Å indicates the development of bound gas that is not removed during heating. Reproduced with permission from ref (369) under CC BY 4.0 license.

The importance of sorption and desorption behavior for applications of microporous materials has led to increasing use of gas flow-cell type setups. Structural transitions induced by dehydration were tracked for MOF Cu-SIP-3 by Allan et al.^320^ Powdered material was heated in a gas flow cell from 300–500 K under He. An assessment of changes in the local structure allowed a more detailed mechanism of a low-temperature to high-temperature transformation to be discerned in which sulfonate groups move to coordinate Cu sites in positions left open by the removed water. Incomplete transformation led to a more disordered state until enough water was removed for the structure to relax into the dehydrated crystal form. Subsequent gas loading experiments with NO showed that it could act in place of water molecules to reform a partially ordered hydrate structure.^320^ The decomposition mechanism of a nerve-agent simulant within the MOF Zr-UiO-67 was recently studied using a variable-temperature, gas-flow setup,^369^ for applications in the remediation of toxic chemical agents. Introduction of dimethyl methylphosphonate (DMMP), a simulant molecule for the nerve agent sarin, was controlled by dosing a He gas flow with DMMP vapor into the MOF. After filling the pores, the remaining DMMP was subsequently removed using a combination of ambient He flow and heating steps to reactivate the MOF. Lattice parameters and on-average pore densities could be correlated with local behavior tracked through dPDF analysis, Figure 32, which showed that DMMMP binds to Zr-oxo clusters and degrades into bidentate-bound methyl methylphosphonate (MMPA).

In situ studies can also be used to study nucleation processes from a solvent or other media. Most work in this area has focused on inorganic nanocrystals;^21^ however, a few studies have also applied these principals to molecular and low-*Z* materials. The crystallization of paracetamol from both 1-propanol and methanol was followed with coupled in situ X-ray total scattering and Raman spectroscopy.^197^ Droplets of solution were levitated in the beam path using an acoustic levitator, then continuously measured as the solvent evaporated and subsequently as the resulting amorphous paracetamol crystallized. The choice of solvent was suggested to have an effect on the local hydrogen bonding that formed during the evaporation process, which acts to template the resulting crystallization into either form I or form II. Nucleation of MOFs from precursor materials have also been investigated. Despite rapid nucleation and growth, a view to the early stage development of ZIF-8 could be achieved by measuring close to the point of mixing between streams of 2-methylimidazole (2-MeIm) in MeOH. A high concentration of Zn(2-MeIm)_4_-type clusters could be confirmed by PDF in conjunction with Zn(2-MeIm)_*x*_(NO_3_)_*y*_-type (*x* ≫ *y*) clusters observed by separate electrospray ionization mass spectrometry measurements.^342^ Xu et al.^343^ observed the formation of hexanuclear zirconium clusters in the metal salt precursor solution already at room temperature, and the formation of a disorder intermediate with structural correlations up to 23 Å prior to the formation of the MOF UiO-66 during solvothermal synthesis.

Other in situ experiments are also possible but challenging. One might consider other external stimuli, for example, the study of light-induced structural modifications associated with photoisomerization of ruthenium sulfur dioxide complexes.^600^ Many PDF studies on milling or compaction effects on the structures of pharmaceuticals have been performed ex situ.^177,183,200^ While, in situ ball milling procedures have become possible in high energy X-ray setups, no in situ PDF studies of milling or other mechanochemical reactions have been performed to our knowledge. This may primarily result from the complexity of the milling apparatuses and difficulty in separating the large and random scattering contributions from balls. However, this is a challenge that we expect will soon be overcome, as PDFs should provide significant value for studying the structural details of nucleating clusters and amorphous intermediates during the complicated reaction pathways induced by milling, including but not limited to small molecules,^601^ COFs,^602^ and MOFs.^603,604^

## 6 Modeling Strategies

So far, we have covered various physical observables that can be detected by PDF methods and the ways in which structural features are manifest in the PDF. We now turn to the problem of how to construct and refine structural models to gain deeper insights. Various schools of thought in approaching these problems exist, often classified as being “small box” and “big box” modeling, and recent discussions on these topics are available.^11,28^

### 6.1 Real-Space Crystal Structure Refinement

Small-box approaches use the minimal number of parameters to describe the structure. This approach generally starts from predetermined candidate models from conventional structure analyses, databases, or sometimes even hand-built models. The models are usually crystallographic models, but they do not need to be (e.g., single molecules, small particles, or other simple models may be used to index structural features in the data). Because the PDF is sensitive to deviations from the crystallographic structure, the local structure can then be explored by comparing different candidate models, or allowing distortions or displacements of atoms or atomic motifs to test whether they improve the agreement between simulated and the experimental PDF. The PDF is calculated after expanding the atoms of the unit cell using periodic boundary conditions;
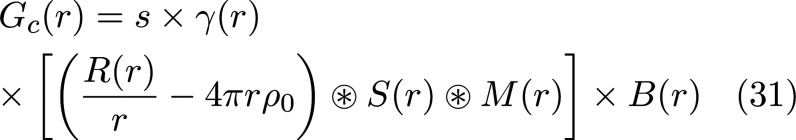
with a scaling factor *s* and characteristic function γ(*r*) describing the crystallite shape and size. Here the circled asterisk symbol indicates a convolution, and *S*(*r*) and *M*(*r*) correspond to effects due to reciprocal-space modifications to *F*(*Q*). The effect of a finite truncation of the data at *Q*_max_ results in *S*(*r*) =  sin(*Q*_max_*r*)/(*Q*_max_*r*). This produces spurious oscillations in the PDF, which come from the data termination and are enhanced by poor signal-to-noise at *Q*_max_. *Q*-dependent modification functions, such as the Lorch and modified Lorch functions, are sometimes applied to suppress the truncation effect.^605−608^ The modification function is multiplied by *F*(*Q*) prior to transformation, or equivalently, its Fourier transform is convoluted with the unmodified PDF. This must also be accounted for in the model (i.e., convoluting *M*(*r*) with the simulated PDF) to properly describe any additional broadening of structural peaks at low-*r*, which can reduce the effective real-space resolution. Although they can aid in visual inspection, the modifications are mostly cosmetic, as the error in the data is not reduced.^609^ It is often better to use only *S*(*r*) to account for termination effects, as long as the data are measured with good statistics. When carrying out difference analysis between measured PDFs, many systematic errors will generally cancel out anyway. Finally, *B*(*r*) accounts for instrumental contributions, including effects of the *Q*-resolution and peak profile, see section 7.7.

The radial distribution is calculated by
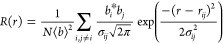
32where the δ functions of eq 1 are replaced by Gaussian peaks due to thermal (and other nonspecific disorder) effects. The broadening factor σ_*ij*_ is defined by the root mean squared displacement coming from the ADP tensors of the atom-pairs and can include functions of *r* to define short-range correlated motion of atoms.^473^ The broadening terms can further include *r*-dependent broadening related to *Q*-dependence of the instrumental resolution (section 7.7).^475,610^

Parameters of the model are refined in a similar manner to Rietveld refinement,^611^ including for instance the lattice parameters of the unit cell *a*, *b*, *c*, α, β, γ, fractional coordinates of the atoms, isotropic or anisotropic ADPs, correlated motion parameters, occupancies, damping functions, etc., to achieve the best agreement with eq 17. Such unit-cell models can also be set up in lower symmetries, for instance by populating all the atoms of the unit cell in *P*1 symmetry, and/or set up as supercells, to access different local structure modifications, for example, different relative orientations of neighboring molecules or modulated structures.

When refining molecular positions or conformations, it is particularly important to utilize rigid body implementations or a corresponding set of constraints and restraints on the atom-pair distances, angles, and torsions to ensure that the structural integrity of the molecule is maintained.^176,177^ Rigid body implementations such as the *z*-matrix format available in TOPAS^612^ or through the pyobjcryst package in Diffpy-CMI^480,613^ make the parametrization of specific conformational distortions and coherent orientational modifications much easier.

A variety of programs are available for accomplishing such refinements. One of the most common and easy to use is PDFgui.^475^ This program works best for quick comparisons, but it is not ideal for molecular materials. It includes a constant peak sharpening parameter **sratio** with a cutoff distance **rcut** to approximate the local σ_*ij*_ behavior for intramolecular correlations, but it cannot explicitly separate intramolecular and intermolecular pair contributions. More accurate fits can be achieved with programs that can accomplish this including DISCUS,^478,479,610^ Diffpy-CMI,^175,480^ or TOPAS v6 and up.^483^ A graphical use interface for Diffpy-CMI for fitting molecular materials is also available.^614^ DISCUS and Diffpy-CMI both provide options to also generate PDFs through the use of the Debye scattering equation, eq 6,^34^ and then propagate to *F*(*Q*) to be Fourier transformed. This is particularly useful for nonperiodic models such as discrete nanoparticles or single molecules. This also allows direct utilization of the *Q*-dependence of the scattering form factors for X-rays and the possibility to implement additional experimental effects such as instrument-specific peak profiles. TOPAS provides a different algorithm for constructing PDFs in real space that results in a significant improvement in calculation speed, especially for models with many atoms, for example, MOFs or large supercell models. This is also useful for running refinements over longer *r*-ranges and/or refining many data sets from, for example, in situ experiments.

### 6.2 Reverse Monte Carlo Modeling

Reverse Monte Carlo (RMC) modeling^615−617^ is an alternative approach to obtain a model that can describe the experimentally observed data. This is a “big-box” approach that starts with a much larger ensemble of atoms (typically thousands) that can be built for instance as a large supercell of a crystallographic model, or obtained from molecular dynamics simulations. An agreement factor is defined by
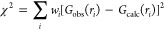
33with *w*_*i*_ as in eq 17. The structure is evolved by randomly selecting and moving an atom and then recalculating eq 33. The updates are based on the Metropolis algorithm^618^ where the agreement between the model and the data takes the place of the energy in the Monte Carlo expression. The move is accepted if χ_1_^2^ < χ_0_^2^, and if χ_1_^2^ < χ_0_^2^, the move can still be accepted with a probability of
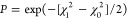
34

Because RMC seeks to maximize the disorder of the given model, it is a good tool for determining a range of atomic configurations that can describe the observed data. However, for the same reason, it has difficulty finding coherent movements of collections of atoms, such as might be expected for large molecules or layers.^509^ Although in principle the Metropolis algorithm is a global optimizer (given sufficient time it will find the global minimum from any starting point), in practice convergence is way too slow for this to succeed, and it is used more as a local minimizer. A good starting model is therefore rather critical in most circumstances. Additionally, it is usually necessary to add additional constraints such as distance constraints to maintain molecular topology or to use rigid bodies for particular molecular motifs.^619^ The choice and testing of different types of constraints can then be used to aid in the determination of factors responsible for features in the experimental data. In sufficiently simple cases, structure determination of locally ordered nanoporous materials has also been demonstrated using RMC.^328,620^

### 6.3 Empirical Potential Structure Refinement

Partial PDFs encode the potential of mean force between the respective atomic species; specifically,
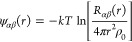
35This allows the development of refinement programs that straddle big box and small box philosophies such as the empirical potential structure refinement (EPSR) method.^224,621,622^ The approach is as follows: (1) parametrize an empirical potential using a small number of refinable parameters; (2) build large boxes of (thousands) of atoms and minimize the energy of the system with respect to the given potential by moving atoms around; (3) compare the resulting PDF to the measured PDF; (4) update the potential parameters; (5) continually repeat the process in such a way as to get the minimum energy structure to give an optimal agreement to the measured PDF. Although there are a large number of atoms, the number of degrees of freedom of the model (the potential parameters) is small, and so there is little danger of overfitting the data and obtaining nonunique results. In more detail, a starting model is constructed for a known molecular species by filling a large box to satisfy the appropriate density of the system. Starting potential functions are guessed for the intramolecular and intermolecular potentials. In most cases, simple harmonic potentials are used for the intramolecular potentials Δ*U*_intra_ to simulate zero-point fluctutations within the molecular structure, while Lennard-Jones plus Coulombic terms are used to describe the intermolecular potentials Δ*U*_inter_. Once the initial system is equilibrated to the reference potentials, the difference between *R*_obs_ and *R*_calc_ is used to generate an empirical potential Δ*U*_emp_, which is then used to perturb the starting potential toward agreement with the experimental data. The energy minimization step also uses a Metropolis approach for which the acceptance criterion for atomic moves is based on the total potential energy of the system. Moves are accepted if they reduced the total potential energy and otherwise accepted with a probability of
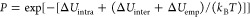
36This approach has been particularly effective for modeling disordered systems such as molecular liquids and glasses.^623−626^ For disordered systems such as this, the information content in the PDF is small, and the success of the technique depends on having more than one differential PDF obtained from neutron scattering of samples that are chemically identical but have a different isotopic composition (section 7.1.2).

### 6.4 Theoretical Methods

Classical molecular dynamics (MD) force fields and quantum mechanical methods such as DFT can be used separately to produce models for comparison against experimental PDF data. Compared to the previous methods, the resulting structures are not biased against the measured data, and this can therefore be used to test the effectiveness of certain force fields or DFT methods for reproducing features of the data such as bond lengths, coordination numbers, or further intermediate-range environments. This approach has been recently successful in testing the idea of polymorphous networks for describing the nature of materials consisting of locally low-symmetry related states that lead to on-average, high-symmetry macroscopic structures, for example in halide perovskites,^627^ and iron superconductors.^628^ Care should be taken as such approaches can be biased by the starting configurations fed into the relaxation tool of choice, and therefore may require a variety of different starting states when attempting to determine best candidate models, especially for a less-well-defined system.^137,369,424^ Another possibility to mitigate poor starting models from either bad guesses or insufficient experimental data is to use an iterative combination of structure refinement to experimental data and molecular relaxation by theoretical methods.^629,630^ For complex, hierarchical simulations, the multitude of contributions to the simulated real-space signal may make it difficult to discern for which structural properties the simulation succeeds and for which it fails. Recent publications by Keffer and co-workers have suggested an approach to decomposing various atomic- and mesoscale structural properties of composite models from large-scale MD simulations.^631,632^

### 6.5 Complex Modeling

Complex modeling encompasses the idea of combining information from separate experimental probes into a global minimization routine. This should optimally leverage the use of more physical/chemical information on the sample available to constrain the model and additionally reduce the effect of uncertainties or systematic errors from any one measurement. This idea is most commonly observed in many RMC and EPSR studies where the experimental reciprocal-space data are often used as a coconstraint in the structural evolution.^223,317,488,625,633^ This mitigates the tendency of the Metropolis algorithm to disorder the structure in the absence of the distinctly separated Bragg peak information that enforces long-range order on the solution. A combination of real-space and reciprocal-space simulated annealing and/or Rietveld corefinement, for example, can be easily implemented using TOPAS v6 and higher.^483,612,634^ Reciprocal-space corefinement is of course only the tip of the iceberg in complex modeling. Other examples include the combination of TEM or SAS for domain size/shape/distribution,^447,450^ and NMR or EXAFS for additional information on local chemical environments.^74,635−639^ A challenge with complex modeling is the optimal weighting of the different data contributions, especially if they have overlapping information content (such as PDF and EXAFS) but different uncontrolled for systematic errors. Significant conclusions should be checked that they are somewhat robust to these factors.

## 7 Experimental Considerations

### 7.1 Scattering Probes

**Figure 33:**
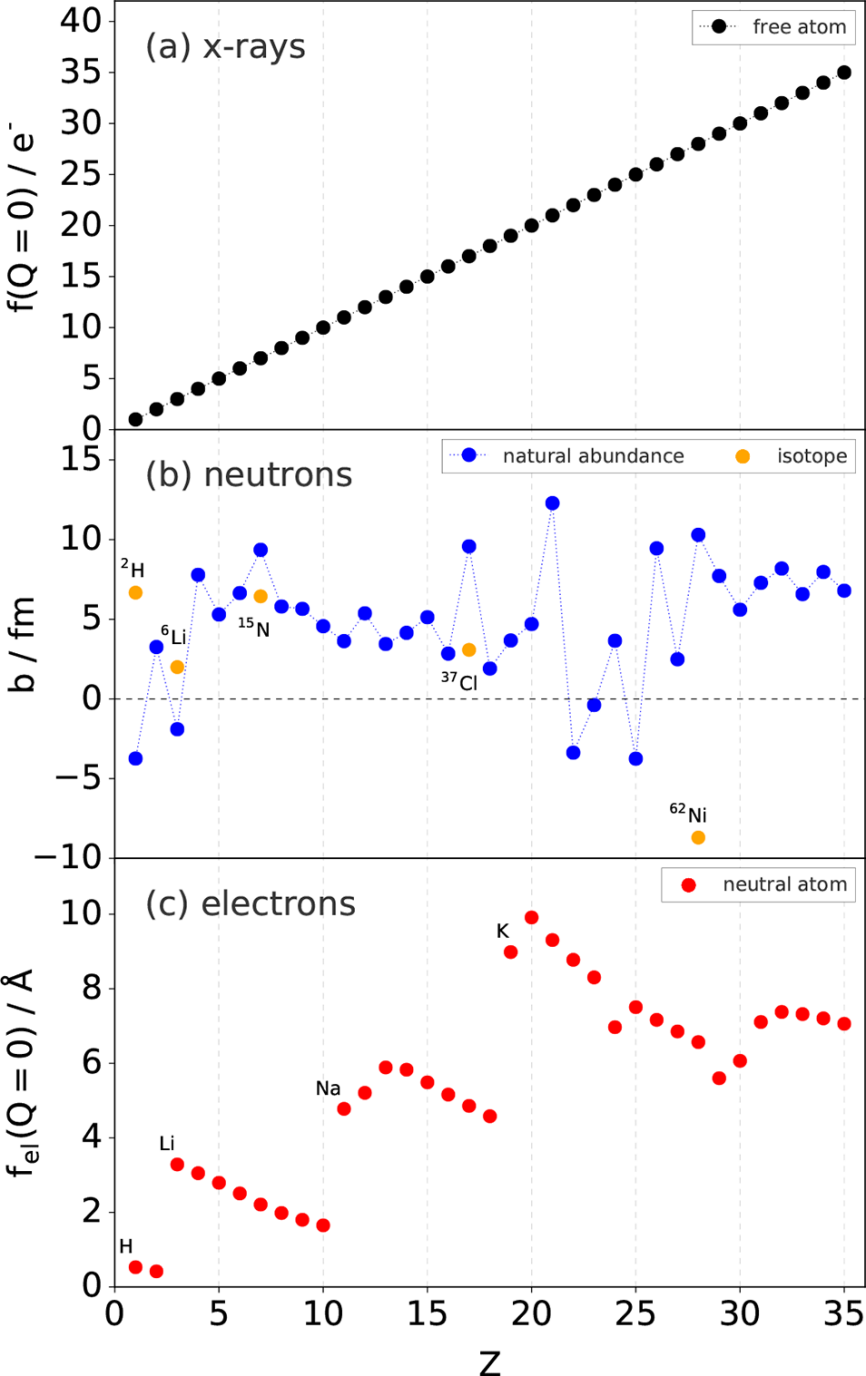
Relative scattering strength and contrast: (a) atomic X-ray form factors *f*(*Q* = 0), (b) coherent neutron scattering lengths for natural abundance elements plus selected isotopes, and (c) atomic form factors for electrons of neutral atoms *f*_el_(*Q* = 0).

In this section, some practical aspects of using different types of radiation are discussed. In addition to varying advantages and disadvantages between the use of X-rays, neutrons, or electrons, one of the primary benefits of using these different probes is in their differing ability to “see” different elements. This is encoded in the weights that contribute to the relative strengths of peaks from different atom-pairs in the PDF, as shown in Figure 33. The *Q*-dependence of form factors must be additionally considered for X-rays and electrons.

#### 7.1.1 X-rays

**Figure 34:**
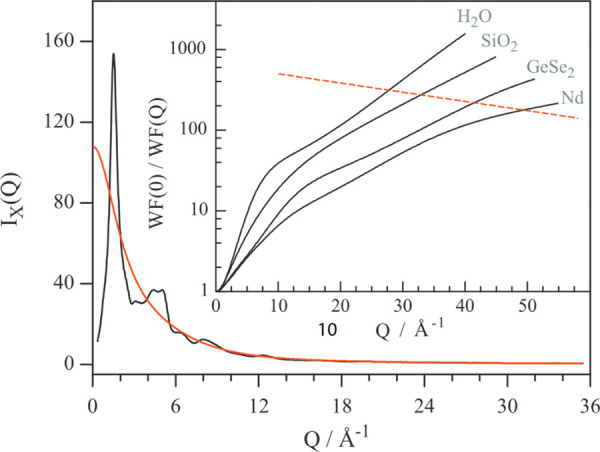
Effect of X-ray form factor: corrected elastic scattering *I*_*x*_(*Q*) from SiO_2_ glass (black) and its X-ray form factor (red). The inset shows the weighting function [WF(*Q*)] due to the division of *N*⟨*f*⟩^2^ in eq 7. The red dashed line represents the *Q*-value of equal accuracy raw measurements with errors in *I*_*x*_(*Q*) in the range of 0.05–0.5%, considering the increasing magnitude of the correction factors. This demonstrates that it is about as difficult to obtain *S*(*Q*) of water out to ∼25 Å as it is to measure for GeSe_2_ to ∼40 Å. Reproduced with permission from ref (640). Copyright 2012 Elsevier.

X-rays are the most widely available and probably the most versatile probe for carrying out total scattering measurements on molecular materials. The scattering power depends on the electron density of the sample (i.e., the species of atoms and their spatial distribution within the sample), roughly proportional to *Z*^2^. The scattering from low-*Z* materials, especially organics and many inorganic molecular materials such as water, produces a much weaker coherent scattering signal than, for example, many typical metals and ceramics. The form factors are also highly *Q*-dependent, damping with increasing *Q*, and more significantly for low-*Z* elements due to the lesser number of core electrons. This results in a relatively weaker signal at higher *Q*. During the data reduction, the division by smaller ⟨*f*(*Q*)⟩^2^ values amplifies the uncertainties in the resulting *S*(*Q*), as shown in Figure 34. Measurement times of just a few seconds are often quoted for in situ measurements of even inorganic nanoparticles,^641^ but low-*Z* materials require longer measurement times to collect data of a commensurate quality. Uncertainties in extracting the high-*Q* signal are further exacerbated by the need to account for other additive and multiplicative abberations to the scattering signal such as subtracting the Compton scattering contribution and instrumental distortions to the measured image or pattern.^640^

An issue with the data reduction for X-rays is that the removal of the *Q*-dependence is only approximate for multielement materials (this issue is relevant to electrons as well). The typical normalization of the scattering using the Morningstar-Krutter-Warren approximation^642^ assumes that the *Q*-dependence of the individual contributions to the total scattering pattern, *f*_*i*_(*Q*)*f*_*j*_(*Q*), can all be suitably described by the square of the atomic concentration weighted average ⟨*f*(*Q*)⟩^2^. However, this is not exactly true, leading to some remaining *Q*-dependent components that get Fourier transformed into the real-space PDF signal. This should be considered during the modeling stages. An exact expression of the PDF accounting for these effects has been derived by separating and modifying the partial contributions from different atomic species to the total PDF.^643^ A more practical approach may be to calculate the scattering intensity of the model from the Debye equation, eq 6, and then propagate to real space in the same procedure as the experimental data. This Debye model approach is already implemented in several modeling softwares (section 6.1),^480,610^ and further allows for other instrument or sample related effects to be applied in reciprocal space.^610^

The validity of the form factors under the independent atom model (IAM) is another consideration when dealing with molecular (primarily low-*Z*) materials, because the formation of a molecule involves redistribution of the electron density. Although the majority of the coherent scattering predominates from the core electrons of the atoms,^644^ the larger proportion of valence electrons for first or second row elements can lead to a more significant impact on the shape of *f*(*Q*). These effects were detailed for instance in an early series of papers by Roy McWeeny,^645−648^ and powerful methods for the analysis of electron charge-densities have since been developed.^649,650^ The effects are most significant for molecules involving hydrogen, and the effects of IAM versus molecular form factors have been studied, for example, for water.^608,651,652^ The deviation between IAM versus molecular form factors measured from the gas is relatively smaller for even second-row molecules^653,654^ and the impact on the intermolecular correlations may be further reduced through orientational averaging.^651^ Thus, the IAM can still be suitable in cases where extreme accuracy in the intramolecular signal is not required, for instance in studying MRO structuring. Good fits with structure models can still be achieved,^32,175−177,376,535^ and the systematic modification to intramolecular signals should cancel when directly comparing experimental data sets. However, with increasingly high quality measurements and more accurate methods for data reduction, these effects may be important for certain use cases. Advancing methods in the field of quantum crystallography for determining the electronic structure^655−657^ may be further considered for implementation in real-space analyses.

The high contrast between light and heavy elements to X-rays can be beneficial, for instance in discerning signals from metal species in organic or aqueous host systems via the difference methods discussed, or for example, to discern structuring of inorganic secondary building units in MOFs. One can also take advantage of the fact that the scattering power of different elements also changes significantly as a function of X-ray energy around the absorption edge of a given element. This is called anomalous differential X-ray scattering.^18,658,659^ In practice, two separate measurements of the same sample are collected, varying the energy of the incident beam in the vicinity of the absorption edge of a particular element in the sample. The results are subtracted, giving the changing signal from the specific element, which can then be used to obtain the dPDF. However, this is less widely used than it might be because of the technical challenges of successfully doing the corrections, and due to the limited *Q*-range relevant to elements in the top half of the periodic table.

In terms of data quality, synchrotron facilities produce optimally high energy, high flux X-ray beams for PDF measurements. The photon flux can be many orders of magnitude higher than what laboratory sources can produce, dramatically shortening the time needed to collect a full data set. The higher energy X-rays penetrate samples better, minimizing absorption in samples with high *Z* elements or with larger diameters, and making it possible to penetrate large or complex sample environments. This allows more flexibility for in situ or in operando measurements. Most beamlines suited for total scattering measurements operate using the rapid acquisition PDF (RAPDF) mode (see section 7.2.1).^437^

Current beamlines with capabilities to collect high quality total scattering measurements for PDF analysis include beamlines 6-ID-D and 11-ID-B at the Advanced Photon Source (Chicago, Illinois, USA),^660,661^ P02.1, P07, and P21.1 at DESY (Hamburg, Germany),^662−664^ I15-1 at Diamond (Didcot, UK),^665^ ID11, ID15A, and ID31 at the ESRF (Grenoble, France),^666−668^ 28-ID-1 (PDF) and 28-ID-2 (XPD) at NSLS-II (Upton, New York, USA),^669^ BL04B2 and BL08W at SPring-8 (Sayo, Hyogo, Japan),^670^ XDS-W09A at LNSL (Campinas, Brazil),^671^ ID1A3 at CHESS (Ithaca, New York, USA), DanMAX at MAX IV (Lund, Sweden), and BL04-MSPD at ALBA (Barcelona, Spain), though this list changes with time.

X-ray total scattering data can also be obtained from laboratory powder diffractometers. Measurement time can be much longer, often 15–30 h for one data set compared to seconds or minutes at a synchrotron. The beam energy is not tunable, but various energies are available through the choice of anode material. A silver anode can produce a *Q*_max_ comparable to synchrotron sources (Ag Kα_1_, λ = 0.5594 Å, *Q*_max_(2θ = 160°) = 22.12 Å^–1^). However, for weakly scattering materials, the statistics of the signals measured at high momentum transfer are often not sufficient to include in the analysis, and so it can be beneficial to trade higher energy for improved statistics using a molybdenum anode (Mo Kα_1_, λ = 0.7093 Å, *Q*_max_(2θ = 160°) = 17.45 Å^–1^). Typical powder diffractometers with a copper anode (Cu Kα_1_, λ = 1.5406 Å, *Q*_max_(2θ = 160°) = 8.03 Å^–1^) are not generally recommended for PDF analysis. The shorter *Q*-range can make data reduction more difficult, because less data dominated by the X-ray form factor at higher *Q*-values is available to constrain the normalization procedure. Furthermore, the nonstructural oscillations in real space (see section 7.4) coming from the truncation of *F*(*Q*) at lower values of *Q* become larger and more difficult to distinguish from the structural signal.^169^ Nevertheless, when carefully measured with very good statistics and to high angles, it is possible to use PDFs from Cu radiation for certain applications such as qualitative phase analysis or determining the extent of structural coherence in amorphous or nanocrystalline samples. There can even be benefits in the increased *Q*-resolution when using lower energy X-rays, as discussed in section 5.8. Overall, monochromatic laboratory diffraction systems and software designed with PDF measurements in mind are becoming increasingly common among many major diffraction system manufacturers.

#### 7.1.2 Neutrons

Neutrons can be highly complementary to X-rays as a probe of the material structure. Their use requires specialized facilities, for example, a nuclear reactor or a spallation source. Spallation sources operated using the time-of-flight method are generally preferred for PDF work due to the high flux of short-wavelength epithermal neutrons. Neutrons scatter off the atomic nuclei, and the scattering power depends on the neutron scattering length, *b*, of the element, as shown in Figure 33. This depends on the isotopic makeup of the nucleus as well as its spin state, and varies across the periodic table and among atomic isotopes in a way that appears somewhat random in contrast to the monotonic increase in the X-ray atomic form factors with *Z*. Neutrons can thus be very useful for gaining better contrast between atoms that are next to each other in the periodic table, or for obtaining increased sensitivity to the lighter elements in samples containing a combination of light and heavy elements. Because *b* is constant in *Q* (unlike the X-ray form factors *f*(*Q*), which decrease with *Q*), data may be collected to larger *Q* values, and approximations needed for removing the *Q*-dependence as with X-rays or electrons become exact. However, even at the highest power modern spallation neutron sources, the neutron flux is orders of magnitude lower than the X-ray flux at synchrotron sources, and neutron scattering measurements therefore take much longer, and generally require larger amounts of sample than X-ray measurements. When preparing for neutron measurements, special considerations must further be made for certain isotopes; for example, hydrogen generates a significant amount of undesirable incoherent scattering. This can make PDF analysis more challenging due to the very large background signal, though there are methods for dealing with this.^451,672^ While not further discussed here, it is worth mentioning that since neutrons also interact with magnetic moments, information about the magnetic structure in a material can be obtained, and the magnetic PDF technique is increasingly used to study magnetic local structures.^673^

The ability to perform isotopic substitutions for certain elements is one of the most important advantages for neutron scattering as it gives a way to experimentally access the partial structure functions *S*_*ij*_(*Q*), and therefore the partial PDFs between different elemental species *i* and *j* in the sample. It has been a critical factor in the characterization of many liquids, as discussed in sections 3.3.2 and 3.3.3, and it is important for the use of the EPSR methods discussed in section 6.3. A sample containing *n* different chemical species has contributions from *m* = *n*(*n* + 1)/2 independent of structure functions, and so *m* samples of differing isotopic composition are required,^674^ along with some detailed algebraic manipulation of the resulting data sets to determine all partial contributions.^17,675^ This of course depends on the stable isotopes available, and sometimes prohibitively, the cost. Arguably the most important isotopic substitution used widely for characterization of molecular systems is deuterium/hydrogen (^2^H/^1^H) substitution.^622,676^ A so-called first order difference can be achieved by subtracting the structure functions from measurements with varying concentrations of deuterium substitution to extract the partial PDF. Since ^1^H and ^2^H have coherent scattering lengths *b* = −3.7406 and 6.671, respectively, a null scattering effect can be achieved by having a ratio of approximately 2:1, for instance by mixing 2 parts ^1^H_2_O and 1 part ^2^H_2_O,^677,678^ to obtain only the O–O partial, and isotopes of other elements can also be used for null scattering.^679^ Double difference or second order difference methods^17^ can be used to further isolate different partial contributions for larger numbers of species *n* by measuring different combinations of isotopic substitution for the same or multiple elements. These methods are important for exploiting the contributions from different molecular species in solutions as well.^256,265^ It is worth noting that even without the use of deuteration, samples with large hydrogen content can still be studied, with proper data corrections, as demonstrated for amorphous pharmaceuticals.^173^

Instruments designed for neutron total scattering measurements include SANDALS, GEM, POLARIS, and NIMROD at ISIS (Didcot, UK),^680−682^ D4 at ILL (Grenoble, France),^683^ and NOMAD and POWGEN at SNS (Oak Ridge, Tennessee, USA).^684,685^

#### 7.1.3 Electrons

Electron diffraction can also be used for PDF analysis. Stemming from pioneering work on the use of electrons for studying the structure of gas molecules by Herman Mark and Raimund Wierl,^686^ Lawrence Brockway and Linus Pauling carried out extensive work in the 1930s using PDFs from electron diffraction to study the structure of inorganic and organic gas molecules.^143−146,687−689^ Transmission electron microscopes (TEMs) have long since been used for PDF analysis of a wide variety of materials, as discussed in recent reviews,^690,691^ and recent developments in this area have helped to improve the quantitative accuracy of the data for more reliable PDF analysis.^692−694^ The coherent scattering signal in this case results from the scattering from the electrostic potential field within the material, so the atomic form factors are different than for X-rays, but also *Q*-dependent.

In contrast to the special facilities required for neutrons or synchrotron X-rays, the electron beams generated from typical in-house TEMs are suitable for obtaining PDF quality scattering data. One of the benefits of electrons is that they interact much more strongly with the sample than X-rays or neutrons, resulting in a stronger scattering signal. This means that significantly less sample is needed to obtain a suitable measurement, and the electron beam can be tuned to a very small size for spatially resolved measurements.^695,696^ For nonamorphous materials, this is increasingly difficult, because smaller sample volumes make it harder to obtain a sufficient orientational average of the coherent scattering domains. Also, strong inelastic and multiple scattering effects present a significant challenge, making both the experimental and sample conditions as well as the corrections during data reduction increasingly important. Damage to the sample from the electron beam can also be significant. The use of electron diffraction PDF has been less common for condensed molecular and/or organic materials, though some examples include organic pigments,^697^ [Mg(DME)_3_]^2+^ molecular clusters,^698^ and glassy water.^247^ Beam damage can be minimized even for molecular systems by reducing the incident beam energy and current, but should be monitored carefully.^697^

### 7.2 Experimental Setups

#### 7.2.1 Rapid Acquisition

**Figure 35:**
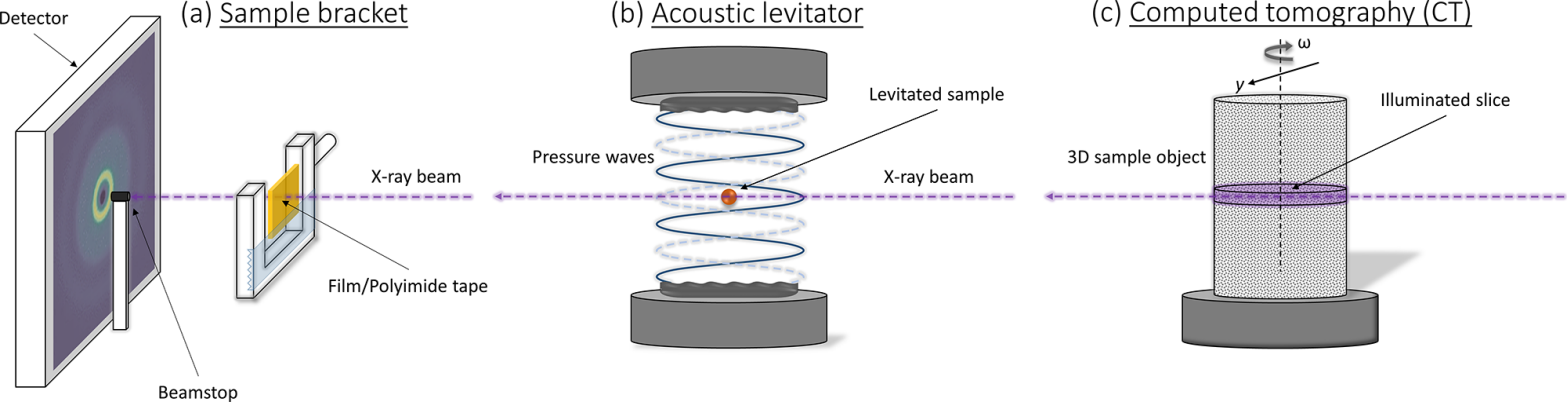
Examples of nonstandard sample geometries coupled with a rapid acquisition setup: (a) film transmission geometry, (b) acoustic levitation as used for small droplets or beads of material, and (c) computed tomography (CT) setup for spatially resolved data with sample rotation angle ω and translation position *y*.

**Figure 36:**
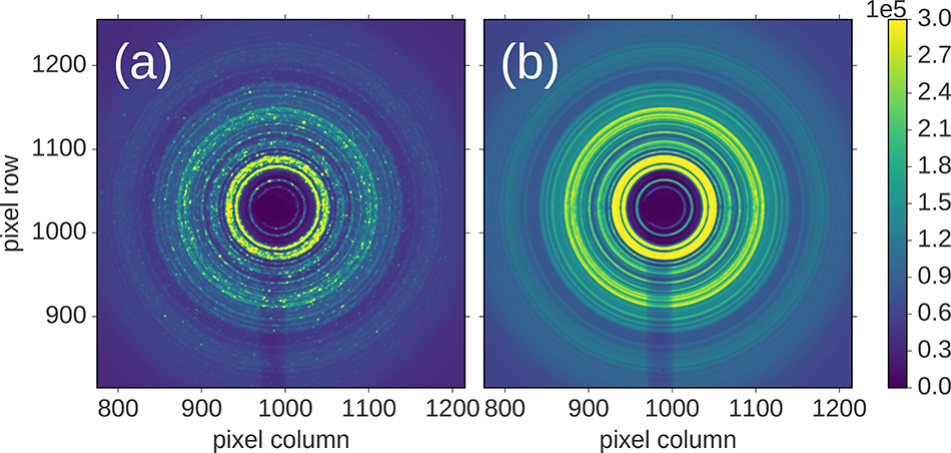
Effect of spinning the capillary on the azimuthal intensities in a 2D diffraction image: (a) without spinning and (b) with spinning to obtain a better powder average, for a small inorganic molecular species.^700^

Standard X-ray PDF experiments are generally performed using the RAPDF setup.^437^ A large area 2D detector is used to simultaneously collect a large region of reciprocal space, resulting in typical measurement times of a few minutes or less. In combination with the high energies (e.g., 50–100 keV or higher), the detector is placed close to the sample (e.g., 150–300 mm) so that a large *Q*_max_ can be measured in a single shot. The RAPDF method generally results in scattering data that has relatively low *Q*-resolution. Therefore, measurements using a second detector, or by moving the primary detector back, can be measured for complementary reciprocal-space analyses. Schematics of the RAPDF setup with different sample geometries are shown in Figure 35. Samples are typically loaded into capillary tubes made of weakly scattering material such as polyimide or borosilicate with thicker diameters than for high-resolution measurements, for example, 1–2 mm, to increase statistics. Most beamlines are standardized for measuring capillary samples, and so anything that can be loaded into a capillary should be—polycrystalline or amorphous solid powder, gels, creams, waxes, etc. Even liquids can be loaded into capillaries using a syringe. The use of liquid suspensions may also be relevant for materials that are stable in solution but not air. For polycrystalline powders, it is especially important to collect a good powder average, meaning the orientational average over all directions of the crystallites or scattering domains. This typically requires grinding the sample, although this may not be possible if the material is mechanically unstable, and further consideration of preferred orientation may also be necessary if the particles are highly anisotropic. Detailed discussion on sample preparation for powders is available.^699^ Spinning the capillary during the measurement can help further decrease the effects of coarse powders, as seen in Figure 36.

#### 7.2.2 Thin Films

Thin films have been measured using a simple transmission geometry.^526,702,703^ The high flux at synchrotron light sources allows sufficient scattering statistics to be collected on both film and substrate, such that the substrate scattering can be directly subtracted. It is preferable to maximize the ratio of thin film to substrate signals by using a weakly scattering substrate material when possible, such as disordered polymer or glass, and by making the substrate as thin as feasibly possible. Amorphous substrates are favored for simplifying the background subtraction. However, it is possible to subtract the signal of a polycrystalline substrate as long as there is a consistent powder average of the substrate material for both sample and background measurements. It is even possible to subtract the background for a single crystal substrate if the crystallographic orientation of the substrate is consistent. For oriented crystals, strongly focused Bragg diffraction spots and streaks can have intensities that are many orders of magnitude higher, which can cause severe detector saturation. This must be avoided by attenuating the incident beam intensity, but this will then make longer measurement times necessary to collect sufficient scattering statistics from the sample film. If possible, background scattering should be measured from the same substrate as the deposited sample to decrease the likelihood of any deviation in substrate thickness, texture, structure, and so on. Significantly longer data collection times may be needed for all types of samples due to the very small amount of material scattering a signal, and this is increasingly difficult for low-*Z* materials. The application of this geometry has been demonstrated for a 130 μm thick layer of organic material for a 10 min exposure time,^704^ and it is thus expected that success for thinner organic films can still be achieved with longer measurement times.

A grazing incidence geometry has also been developed for total scattering measurements of thin and ultrathin films.^664,705^ This is a more specialized setup than the transmission measurement, but it allows for information specific to the surface structure to be obtained.

Preferred orientation of the crystallites or scattering domains can be more difficult to deal with in thin film geometries. Films typically cannot be further processed as powders, without completely changing the nature of the material. Thus, the techniques as used for typical PDF analysis are more suited toward amorphous and nanoscale polycrystalline films. In cases where the films have much larger domains, or are subject to severe preferred orientation, different methods for data reduction and analysis will be necessary, and new methods are being developed for handling this.^610,706,707^

#### 7.2.3 Spatially Resolved Measurements

In addition to the grazing incidence geometry to access information specific to the surface of the sample, other spatially resolved measurements can be performed. This can be very simple, for instance by scanning along different spots of a capillary to test the bulk homogeneity of a powder sample.^177^ One- and two-dimensional scans can be carried out on films or arrays of samples, and high spatial resolution can be obtained by using thinner, micrometer-sized X-ray beams,^668^ or by using electrons.^695^ Even three-dimensional information for larger objects can be determined using computed tomography,^708^Figure 35c. The use of smaller beams of course results in poorer statistics in the data collection, which is already a limiting feature for low-*Z* and especially organic materials. This will also significantly limit the total scan area or volume that can be sufficiently probed. However, with suffient measurement times and increasingly powerful X-ray sources, we expect that feasibility will increase. The spatial mapping of the local structure in an amorphous, organic films has, in fact, been demonstrated using scanning transmission electron diffraction techniques.^696^

### 7.3 Sample Environments

Various sample environments and apparatuses are available at different beamlines for the study of materials under diverse and highly specialized conditions, for in situ and in operando studies. Heating and cooling devices are standard at most total scattering beamlines. For instance, cryocooling systems are common with temperatures in ranges from 77–500 K using flowing nitrogen gas from a liquid nitrogen source, and liquid helium cryocoolers that can theoretically reach as low as 4 K. Improved thermal stability at ultracold temperatures can be obtained using vacuum pumps to suck out the air, for example in a cryostat device, and other heating devices, and X-ray penetrable furnaces are commonly available to reach much higher temperatures. Various reactor systems have also been developed for studying reactions including gas and liquid flow cells,^342,709,710^ sputtering chambers,^664^ pressure cells,^711,712^ electrochemical cells,^713,714^ and microwave reactors.^715^ Superconducting magnet environments are even available at the 28-ID-1 beamline at NSLS-II and P21.1 at DESY, which could open the door to studies of magnetic field effects on the structure of single-molecule magnets,^716,717^ magnetic polymer composites,^718^ or secondary building units with magnetic elements in MOFs.^719^

Specialized setups have been developed for combining multiple experimental probes with total scattering measurements including acoustic levitation and Raman scattering,^197^ FT-IR,^668,720^ and a laser heated diamond anvil cell with micro-Raman spectroscopy.^721^ Many other potential setups for different environmental conditions, reaction types, or combined methods exist. To get details of what sample environments are available, it is generally a good idea to check the web-page of the beamline in question, and reach out to the instrument scientists with questions.

### 7.4 Measurement Range

In addition to the physical limits of the detector due to its finite size and position, the end-measurement data quality (e.g., the level of errors and measurement statistics at high *Q*) will determine the effective *Q*-range that should be included in the Fourier transformation. The real-space resolution is given by the Nyquist-Shannon sampling theorem, where the smallest meaningful interval is Δ*r* = π/*Q*_max_.^722^ It is theoretically desirable to maximize the experimental *Q*_max_ to get the best possible resolution and minimize truncation effects, but this it not always practical or useful. For weakly scattering materials, the relatively higher amount of incoherent scattering background to coherent scattering signal at higher *Q* values and the division by smaller valued weighting functions (discussed in section 7.1.1) make it very difficult to accurately measure the higher-*Q* signal (e.g., >20–25 Å^–1^) with suitable statistics. Although, this is becoming less of a problem with the ultrahigh flux available at modern synchrotron sources. To obtain good quality PDFs, the *Q*_max_ should be adjusted to minimize the amount of *Q*-space included that is measured with poor signal-to-noise. The combination of noisy signal and data truncation result in unphysical, high frequency oscillations in the resulting PDF (the truncation effect is discussed in section 6.1), which can obscure small features in real space. In the worst case, these errors may be misinterpreted as structural features.^701^ This can also lead to slight variations in the relative peak amplitudes that may affect the accurate refinement of certain parameters such as occupancy values, if refined. Large thermal displacements in many molecular materials can reduce the amount of coherent signal at high *Q*, and so in some cases there is limited benefit in measuring to higher *Q* anyways.^608^ Evaluation has shown that with increasing thermal displacements, lower *Q*_max_ values can be used with minimal truncation errors in the resulting PDF.^609^ Generally, if one knows in advance the real-space resolution required for the scientific goal, or the effective *Q*_max_ that can be measured with suitable signal-to-noise (e.g., from previous measurements), then the experimental setup can be optimized to just measure over this range.

In some cases, the choice of the minimum value, *Q*_min_, may also be important. This is generally the case when SAS from small particle size or other morphological features overlaps into the diffraction features from the atomic structure.^33^ The observation of low-frequency oscillations extending to the high-*r* region of the PDF, which are especially observable in amorphous or liquid signals that are otherwise flat in this region, may signify the existence of a scattering signal in the low-*Q* region of the measured diffraction pattern. A hint that your data are subject to this issue is that the wavelength of the oscillatory feature is ∼2π/*Q*_min_, and it varies with *Q*_min_, something that is rather easy to check. This implies that some low-angle feature in the correlations is missing from the data due to the *Q*_min_ cutoff, but the Fourier transform does not know where in *Q*-space that feature actually resides (because its signal is lost). If there is no missing low-*Q* signal then features in the PDF will not depend on the value of *Q*_min_. It is very important to carry out this *Q*_min_ test before drawing strong conclusions from the low-frequency ripples in the high-*r* region of the PDF. Such assessments have been important in characterizing the extent of atomic order associated with noncrystallographic pore structures,^341^ poorly ordered trimeric macrocycles,^463^ and recently in ascertaining quasiperiodic extended-range order in a MOF liquid.^336^ It is important to note that for many large-pore frameworks materials, e.g., MOFs and COFs, diffraction peaks from the structure often occur at considerably low values in the range of 0.1–0.5 Å^–1^, and the detector and beamstop positions must be adjusted to capture these features in the measurement.^324,341,376^

### 7.5 Data Quality and Background

**Figure 37:**
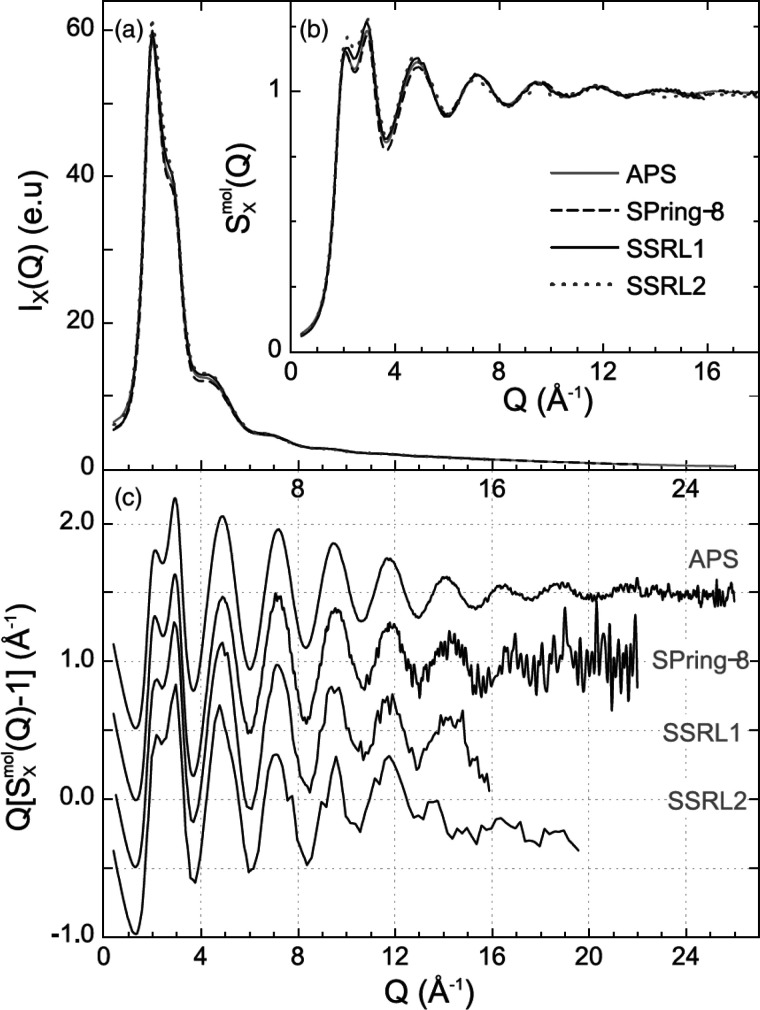
Comparison of four different synchrotron X-ray diffraction measurements of liquid water at ambient temperatures: (a) the total scattering intensity normalized to electron units, (b) the structure factor obtained by normalization to the X-ray molecular form factor for water, and (c) the reduced structure function, showing more clearly the varying data quality of both the signal-to-noise at high *Q* and the quality of the corrections applied in the data reduction. The data are from refs (701 and 723−725) and the figure is reproduced with permission from ref (608). Copyright 2013 AIP Publishing.

An important consideration for data collection is the scattering strength of the sample (constituent elements and atomic density) for the given radiation, and subsequently the total summed time of the sample exposure, to ensure the collection of sufficient scattering statistics. The estimated standard deviations of the scattered intensities measured with *N* counts is governed by . The propagation of errors from the measurement to the PDF is covered in detail,^441^ though it can become increasingly complicated, for example, when considering different types of detector technologies.^523^ We typically do not know a priori how much scattered radiation we will collect from our sample, because this also depends on the radiation energy, beam flux, sample-to-detector distance, sample composition, sample volume in the beam, etc. From a more practical point-of-view, it is rather useful to track the statistics as the data are collected, for instance by summing individual exposures. Since the structural signal at high *Q* is generally difficult to observe in the diffraction pattern, as shown in Figure 37a, it is better to carry out the data reduction to track the changes in *F*(*Q*) and *G*(*r*) with increasing summed exposure time. The experimenter may measure until the high-*Q* signal in *F*(*Q*) is recorded with good signal-to-noise and when the contribution of noise to the nonstructural, high frequency ripples in *G*(*r*) cannot be further reduced for a given *Q*_max_. The *F*(*Q*) functions for different quality measurements of water are shown for comparison in Figure 37b.

It is additionally important that contributions to the background scattering are minimized, such as scattering from air, the sample container, and any other external equipment.^179,640^ This is especially important when the scattering signal from the sample is broad and/or containing important features at low-*Q* values for which the background intensities are highest.^568^ Containers are typically made of thin walled, weakly scattering, amorphous materials, but air scattering is often the dominant contribution to the background, and this can be reduced by good shielding and collimation of the beam up to the sample, shorter sample-to-detector distances, and in extreme cases, the use of sample chambers or flight tubes filled with He or under vacuum. At high energies, and particularly for weakly scattering materials and most molecular materials, the attenuation of the beam due to the sample and background are small, and a direct subtraction is often sufficient, aided for instance by the background subtraction and correction methods implemented in, for example, PDFGetX3^726,727^ (see section 7.6). The background should be collected with good statistics so as to minimize the statistical uncertainties added to the sample measurement. Because background signals are often smoothly varying in *Q*, they are sometimes smoothed to reduce the added statistical errors, but a simple rule of thumb is to measure for longer than the sample measurement. In cases for which sample attenuation is not negligible, angular dependence of the background contribution can arise, and there are approaches to correcting for this, involving the measurement of all the separate contributions to the background individually.^640^

### 7.6 Detector and Scattering Corrections

Corrections to the collected scattering intensities depend on many details including both experimental (e.g,. radiation source, energy, geometry, background contribution, detection hardware etc.) and sample-dependent factors (e.g., sample packing fraction and volume, absorption, multiple scattering, Compton scattering, fluorescence, etc.), the effects of which are well documented.^2,18,728^ The significance of capillary sample absorption and possible fluorescence effects for a given experiment may be conveniently checked online.^729^ The additional scattering contributions can strongly depend on the type of radiation used and the material of interest, for instance strong inelastic^730^ and incoherent^672^ neutron scattering effects for certain materials, or strong multiple scattering for larger crystallites by electrons.^692,731^

In many cases, the exposure during the measurement must be carefully controlled, for example, for amorphous silicon detectors, where the pixel response can be oversaturated by strongly scattering materials. In these cases, smaller sample volume, attenuation of the beam flux, or shorter exposure times can be utilized. Measured intensities should generally be kept well below the saturation levels, as trapped excited states can remain after long periods of high exposure, leading to unwanted features in the pixel response that must be removed.^640^ Other considerations may be necessary for other detection technologies such as Si and CdTe hybrid photon counting detectors.^732,733^ Diffraction images must be calibrated and corrected for polarization, spatial distortions,^734,735^ unequal pixel response and parasitic scattering effects.^640^ It is further important to apply proper masking of the images to remove anomalously dark or bright pixels, dead pixels, discrete diffraction spots caused by a poor powder average, or other effects such as shadows from any equipment located in the cone of diffracted radiation. Masking can be performed using a statistical analysis of the azimuthal ring intensities to determine an acceptance criterion within a specified range of standard deviations relative to the mean or other value calculated for the azimuthal intensities, as implemented within xpdtools.^736,737^ The corrections and azimuthal integration of 2D diffraction images can be performed using various software packages include Fit2D,^738^ pyFAI,^739^ DAWN,^740^ and srXplanar.^727^ For neutron experiments, measurements generally involve many separate detector banks, and thus merging processes are more complicated and handled on-site.

Different methodologies for determining the PDF have been developed, varying primarily in the radiation type, the level of assumptions and approximations made, and ease of use. After experimental abberations to the data have been taken into account and corrected, the remaining, unwanted scattering contributions must be removed, and the data must be normalized and Fourier transformed. The incoherent Compton scattering dominates the scattering at higher momentum transfer, especially for low-*Z* materials.^18,741^ This contribution can be removed by energy discrimination of the scattered radiation,^742,743^ though in most cases is removed later during the data reduction. Subtraction of the incoherent signal, in addition to division by the form factors, and further multiplication by *Q* can all exacerbate errors or poor counting statistics of the coherent structural signal at high *Q*, particularly for X-rays and electrons. This increases the need to minimize systematic error and obtain very good counting statistics in the measurement. Other effects combine to form additional additive and multiplicative modifications to the measured signal. Various data reduction packages are available. Data reduction packages include:

- X-rays: RAD,^744^ PDFGetX2,^745^ GudrunX,^728^ PDFGetX3,^726,727^ GSAS-II,^746^ TOPAS v7.^634^
- Neutrons: PDFGetN,^747^ GudrunN,^728^ PDFgetN,^747^ PDFGetN3.^748^
- Electrons: eRDF Analyzer,^749^ SUePDF,^750^ ePDF tools,^751^ or commercial software ePDFsuite.^752^

The choice of data reduction software may depend on the goal of the analysis. For most cases involving data-to-data comparisons or small-box modeling approaches, the reduction method provided by PDFGetX3 can be recommended,^726^ for generating the PDF from integrated total scattering patterns. PDFGetX3 performs these corrections in an ad hoc way by parametrizing the corrections using a low-order polynomial to correct for slowly varying deviations to the known asymptotic behavior of the *S*(*Q*) and *G*(*r*) functions.^436,753^ The only inputs needed are the experimental sample and background scattering intensities and the chemical composition of the sample. However, certain analysis goals may necessitate use of a program that implements explicit corrections for experimental aberrations. For example, large-box models, such as analysis using RMC, EPSR, or similar approaches, will be best applied to data for which systematic errors have been formally mitigated. This motivates the use of, for example, PDFGetX2 or the Gudrun family of software. These packages attempt to correct the data by applying formal corrections, to best approximations, for many of the effects listed above. As this also gives the best chance for achieving PDFs on an absolute scale, this is also probably the best option for those who wish to extract coordination numbers (eq 2) from the peaks without an explicit structure model. PDFgetX3 results in accurate PDFs, but the scale factor of the data is not guaranteed; this is not a problem for small-box modeling approaches that include a scale factor as a refinable parameter, and for bulk materials it often does produce good scaling results. Further considerations, such as the direct removal of instrument profile effects, may further inform the choice of the data reduction method, as discussed below.

### 7.7 Instrumental Profile

Different aspects of the experimental setup affect both the resolution of the diffraction pattern and the shape of the peak profile, and these further affect the structural signals observed in the resulting PDF. Different considerations must be made depending on the geometry of the measurement, the kind of detector used, and the optics controlling the shape and size of the incident beam. These effects can be quite complicated. For an overview, good discussions of instrumental contributions to the peak profile are available,^754,755^ with discussion more particular to PDF data reduction,^18^ and in general different considerations are necessary for laboratory^756,757^ versus synchrotron diffractometers.^758^

The most significant effect of decreasing resolution in reciprocal space is to damp the real-space signal with increasing distance. Fortunately, for typical RAPDF measurements at synchrotrons,^437^ it is often sufficient to consider the instrument resolution function to be Gaussian. Therefore, the Fourier transform is another Gaussian function that can be defined as
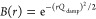
37which is multiplied by the theoretical PDF without any modification due to the instrument (i.e., the *B*(*r*) function mentioned in section 6.1). The *Q*_damp_ parameter is determined by measurement of a crystalline calibration standard such as Ni, CeO_2_, Si, or LaB_6_ and then refining a structure model to the PDF. The resulting value is then fixed for refinement of further samples measured using the same experimental setup. The lower is the resolution in *Q*-space, the more damped is the PDF signal, which effectively reduces the total distance over which the structure can be analyzed and greatly affects the ability to estimate domain sizes (section 4.1) when the structural damping is larger than the instrumental damping. This has been discussed systematically,^436,438,439^ and for practical use-cases.^32,139,177^ Improved descriptions of the damping effects are being developed for cases where the peak profile cannot be considered Gaussian.^759^

**Figure 38:**
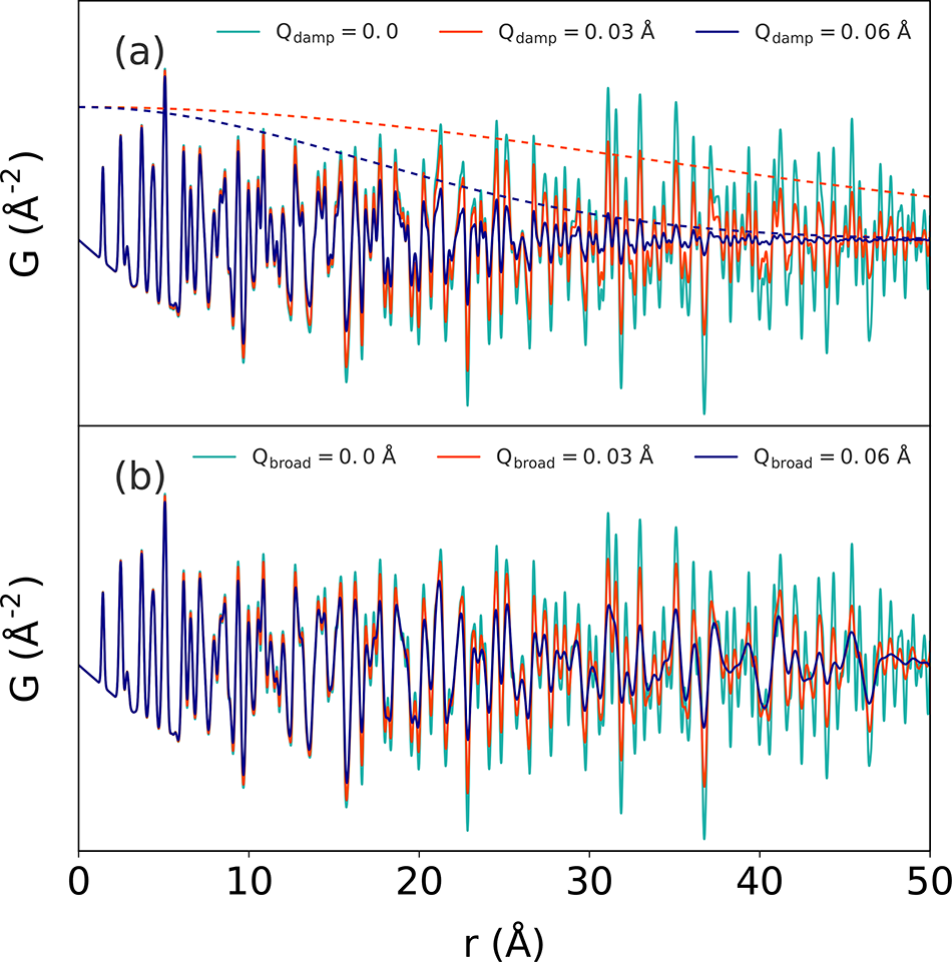
Modification of a simulated PDF due to the parameters used to describe different reciprocal-space instrumental resolution effects: (a) The effect of decreased reciprocal-space resolution is modeled by increasing *Q*_damp_ to damp the real-space signal at higher distances. (b) The effect of nonconstant reciprocal-space resolution is modeled by *Q*_broad_ to broaden the real-space signal at longer distances.

Another consideration is that the resolution of the detector is often not constant with respect to *Q*, rather a function of *Q*. This can be due to different effects, such as beam divergence,^760^ or due to the nonconstant sample-to-detector distance with increasing angle for flat detectors. Examples of the resolution functions for different total scattering setups are available.^663,705,761−764^ The primary effect is to introduce an *r*-dependence to the peak broadening in real space. This has been accounted for by including an extra *r*-dependent term, separate from the atomic displacement parameters, in the description of the peak widths,^438,609,610^ for example, called *Q*_broad_ in Diffpy-CMI and DISCUS. The basic effects of *Q*_damp_ and *Q*_broad_ are demonstrated in Figure 38.

Many effects due to the instrumental profile have been shown to be very small in the short-*r* range of the PDFs, below ∼20 Å.^439^ This can be beneficial when the scientific goal only requires investigation of very short-range structures, because this range is more robust to inaccuracies in the parameters used to model the reciprocal-space profile effects. More accurate descriptions of the resolution function may be more important for generating quantitatively accurate PDFs from laboratory measurements, and increasingly important for investigating the real-space structure over longer distances (see section 5.8). This is also important for neutron experiments for which total scattering data from different detector banks may be combined.^439^ In light of this, new methods for handling the incorporation of more complicated instrumental profiles in addition to other effects such as zero-point offset correction have been discussed for DISCUS^610^ and RMCProfile,^765,766^ and a new method for obtaining PDFs for scattering data by directly correcting the data for instrumental and emission profile effects is now available in TOPAS v7.^634^ Simultaneously, improved measurement protocols can also help to reduce these effects.^764^

## 8 Outlook

The analysis of pair distribution functions is a long-standing and important method used to further the understanding and technological application of disordered materials, including many organic and inorganic molecular materials, and network or composite materials with molecular components, as discussed herein. The techniques have become increasingly accessible to general users through the continued development of specialized facilities, software, training workshops, and through the dedication and innovation within a longstanding, international community of scientists. It should be considered, in any case, a highly complementary tool to the characterization of diffraction data: neither a rival to single-crystal and Rietveld refinement techniques, nor a separate technique to be considered in isolation from good reciprocal-space diffraction data analysis! Used in concert with an all-data-assessment protocol, motivated by the material problem and form of the diffraction pattern, we expect it to continue contributing to major advances in understanding real materials, blemished, defects, imperfections, and all.

We expect continued advances in combining large-scale hierarchical and ab initio molecular dynamics and density functional theory results with local structure characterization to understand and predict properties of polymorphous and network-forming structures beyond the unit cell. A more thorough picture of how local- and mesoscale defects and correlated behavior should continue to develop for hybrid organic–inorganic structures. In conjunction with developments in cryoEM techniques for local scale structure solution, we expect more robust model construction algorithms and regression protocols to deliver more bulk-representative structure solutions of nanostructured materials including complex macromolecules, organic pigments, and pharmaceutically relevant compounds. Finally, increasing beamline infrastructure will enable increasingly complex sample environments, faster time resolutions, and on-demand, real-time structure characterization and feedback for data-driven chemistry.
